# Evolution and Domestication of a Novel Biosynthetic Gene Cluster Contributing to the Flavonoid Metabolism and High‐Altitude Adaptability of Plants in the *Fagopyrum* Genus

**DOI:** 10.1002/advs.202403603

**Published:** 2024-09-23

**Authors:** Xu Huang, Yuqi He, Kaixuan Zhang, Yaliang Shi, Hui Zhao, Dili Lai, Hao Lin, Xiangru Wang, Zhimin Yang, Yawen Xiao, Wei Li, Yinan Ouyang, Sun Hee Woo, Muriel Quinet, Milen I. Georgiev, Alisdair R. Fernie, Xu Liu, Meiliang Zhou

**Affiliations:** ^1^ National Key Facility for Crop Gene Resources and Genetic Improvement Institute of Crop Sciences Chinese Academy of Agricultural Sciences Beijing 100081 China; ^2^ Department of Agronomy Chungbuk National University Cheongju 28644 South Korea; ^3^ Groupe de Recherche en Physiologie Végétale (GRPV) Earth and Life Institute‐Agronomy (ELI‐A) Université Catholique de Louvain Croix du Sud 45, boîte L7.07.13 Louvain‐la‐Neuve B‐1348 Belgium; ^4^ Laboratory of Metabolomics Institute of Microbiology Bulgarian Academy of Sciences Plovdiv 4000 Bulgaria; ^5^ Center of Plant Systems Biology and Biotechnology Plovdiv 4000 Bulgaria; ^6^ Department of Molecular Physiology Max‐Planck‐Institute of Molecular Plant Physiology Potsdam 14476 Germany

**Keywords:** buckwheat, evolution, flavonoid metabolism, novel gene cluster, UV resistance

## Abstract

The diversity of secondary metabolites is an important means for plants to cope with the complex and ever‐changing terrestrial environment. Plant biosynthetic gene clusters (BGCs) are crucial for the biosynthesis of secondary metabolites. The domestication and evolution of BGCs and how they affect plant secondary metabolites biosynthesis and environmental adaptation are still not fully understood. Buckwheat exhibits strong resistance and abundant secondary metabolites, especially flavonoids, allowing it to thrive in harsh environments. A non‐canonical BGC named *UFGT3* cluster is identified, which comprises a phosphorylase kinase (*PAK*), two transcription factors (*MADS1/2*), and a glycosyltransferase (*UFGT3*), forming a complete molecular regulatory module involved in flavonoid biosynthesis. This cluster is selected during Tartary buckwheat domestication and is widely present in species of the *Fagopyrum* genus. In wild relatives of cultivated buckwheat, a gene encoding anthocyanin glycosyltransferase (AGT), which glycosylates pelargonidin into pelargonidin‐3*‐O‐*glucoside, is found inserted into this cluster. The pelargonidin‐3*‐O‐*glucoside can help plants resist UV stress, endowing wild relatives with stronger high‐altitude adaptability. This study provides a new research paradigm for the evolutionary dynamics of plant BGCs, and offers new perspectives for exploring the mechanism of plant ecological adaptability driven by environmental stress through the synthesis of secondary metabolites.

## Graphical abstract

In this study, a non‐canonical molecular module type plant biosynthetic gene cluster is identified with the dominant haplotype of this cluster being selected during the domestication of Tartary bbuckwheat, in wild relatives of this crop, an additional insertion of an anthocyanin glycosyltransferase gene *AGT* into the cluster confers stronger high‐altitude adaptability to wild relatives of buckwheat.

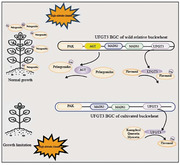

## 1 Introduction

Since plants first colonized terrestrial environments about 500 million years ago,^[^ ^1^ ^]^ they have had to face enormous challenges from the environment. In order to cope with these environmental conditions, plants have evolved multiple strategies for adaptation, such as morphological adaptation, establishing symbiotic relationships with microorganisms, sensitivity to temperature and water changes, complex transcriptional regulation, evoking signal transduction pathways, and the synthesis of secondary metabolites. Flavonoids, belonging to polyphenolic compounds, are important plant secondary metabolites participating in plant‐environment interactions.^[^ ^2^, ^3^, ^4^, ^5^, ^6^ ^]^ Therefore, understanding the biosynthetic mechanism of flavonoids is crucial for exploring evolutionary and domestication mechanisms of plants' adaptation to environmental stress.

So far, over 9000 flavonoids have been identified in plants.^[^ ^7^, ^8^, ^9^ ^]^ Besides being diverse, flavonoid biosynthesis is extremely complex, with multiple intermediates and derivatives interconverted.^[^ ^10^ ^]^ Additionally, it is highly susceptible to environmental influence, meaning that the levels of flavonoids are highly variable and relatively unstable.^[^ ^11^ ^]^ Glycosyltransferases, such as UDP‐glucose‐flavonoid‐glycosyltransferase (UFGT) and anthocyanin glycosyltransferase (AGT), can alter the solubility and bioavailability of flavonoids by glycosylation, thus imparting different functional properties to flavonoids in various cellular components.^[^ ^12^ ^]^ In addition, the biosynthesis and metabolism of flavonoids were regulated by various transcription factors.^[^ ^13^, ^14^, ^15^ ^]^ For example, in *Eriobotrya japonica*, MADS‐box transcription factor EjCAL directly binds to the promoter of the glycosyltransferase gene *EjUF3GaT1* and activates gene expression, regulating the biosynthesis of hyperoside.^[^ ^16^ ^]^ The MADS family transcription factor MdJa2 can directly bind to the promoter of downstream target genes, inhibiting the synthesis of anthocyanins and proanthocyanidins in red‐fleshed apple.^[^ ^17^ ^]^ In buckwheat, MYB and ERF transcription factors have been identified to directly bind to promoters of genes encoding flavonoid biosynthetic enzymes or transporters thus regulating gene expression.^[^ ^18^, ^19^, ^20^ ^]^ These diverse enzymes and transcription factors lead to plants possessing different types and abundances of flavonoids.

The synthesis of metabolites typically involves the coordinated action of multiple genes, which may exist in the form of gene clusters, known asBGCs. Gene clusters (operons) were originally discovered in prokaryotes, where ≈50% of the genes were clustered into gene clusters.^[^ ^21^, ^22^ ^]^ Besides being well‐characterized in bacteria, there has been extensive research on gene clusters in fungi.^[^ ^23^, ^24^, ^25^ ^]^ In plants, BGCs usually consist of a set of genes encoding enzymes in the biosynthesis or modification pathways.^[^ ^26^, ^27^, ^28^ ^]^ For instance, *UDP‐glycosyltransferases UGT85B1* is one of the four core members of the cyanogenic glycoside dhurrin gene cluster in *Sorghum bicolor*.^[^ ^29^ ^]^ UGT85B1 can interact with the proteins encoded by two other members of this gene cluster, CYP79A1, and CYP71E1, forming a channeling complex that facilitates the rapid flow of metabolic intermediates during dhurrin biosynthesis.^[^ ^30^ ^]^ However, in plants, BGCs containing specific transcription factors within gene clusters have not been reported.^[^ ^31^ ^]^ In conclusion, despite the fact that BGCs are unique and important for plant biosynthesis, their regulatory patterns, as well as their formation and evolution, remain incompletely understood.

Metabolomics is a powerful tool for studying the modification of flavonoid compounds.^[^ ^32^ ^]^ In previous research, through GWAS analysis of metabolites contents in Tartary buckwheat germplasm resources, a gene encoding FtUFGT3 was identified as associated with flavonoid contents.^[^ ^33^ ^]^ In this study, enzymatic studies confirmed that FtUFGT3 is a key enzyme in flavonoid biosynthesis, and could catalyze the glycosylation of kaempferol, quercetin, and myricetin. Further analysis revealed that *FtUFGT3*, along with three adjacent genes (two genes encoding MADS‐box transcription factors FtMADS1 and FtMADS2, as well as one gene encoding a protein kinase FtPAK) formed a molecular module type BGC that co‐regulates flavonoid biosynthesis in Tartary buckwheat. This BGC is located within the region that underwent selection during Tartary buckwheat domestication and is widely present in buckwheat plants. In wild relatives of buckwheat, a *UDP‐glucose transferase* (*AGT*) was found inserted into this gene cluster. This AGT could glycosylate pelargonidin into pelargonidin‐3*‐O‐*glucoside, which could help plants alleviate the UV damage thus enhancing the adaptability to high‐altitude environments. This study provides new insights into the molecular mechanisms of flavonoid biosynthesis and ecological adaptation in buckwheat. It reveals the impact of genomic evolution on plant ecological adaptability, contributing to the development and utilization of plant resources.

## 2 Results

### 2.1 FtUFGT3 Is a Crucial Enzyme Catalyzing the Glycosylation of Various Flavonoid Substrates

**Figure 1:**
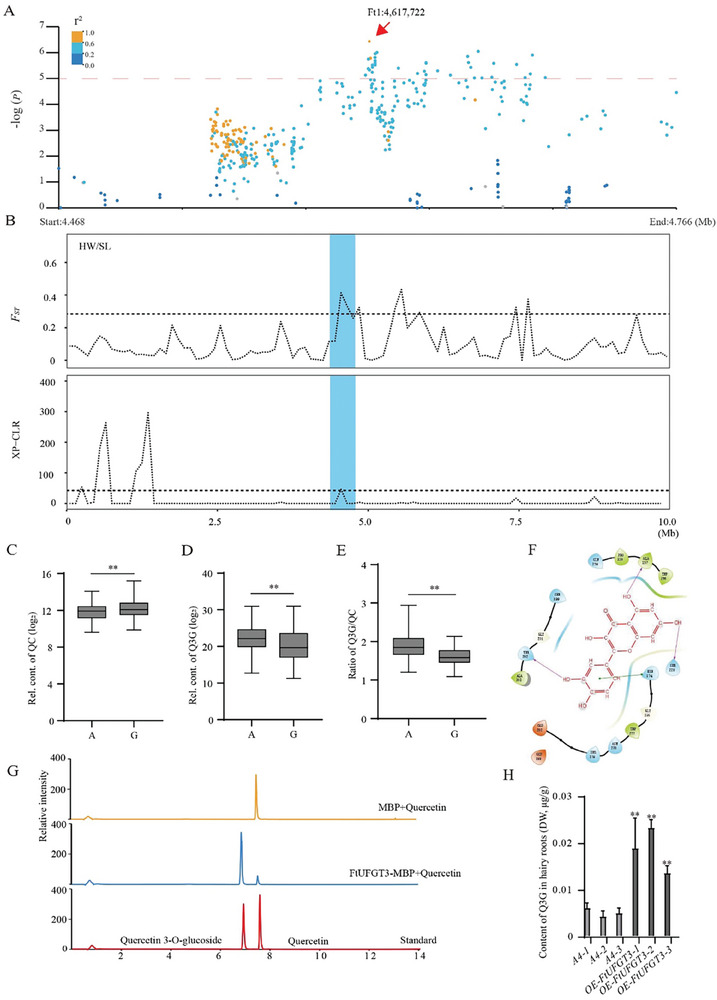
The multi‐catalytic function of FtUFGT3.A) Ft1:4617722 (*FtUFGT3*) was identified through GWAS on quercetin‐3*‐O‐*glucoside in Tartary buckwheat germplasm resources. The dashed line indicates the threshold −log*P* = 5. The red arrow indicates the lead SNP. B) The upper part illustrates population differentiation (*F_st_*) with selective sweeps in Tartary buckwheat. The lower part is the XP‐CLR plot of FtUFGT3. *F_st_* and XP‐CLR are plotted using 10 Mb sliding window. The black horizontal dashed line represents the genome‐wide cutoff with the highest being 5%. C) Box plots show the relative content of glycosylated quercetin in A and G genotypes. QC, quercetin. The content was log2 transformed, **P < 0.01, Student's t‐test. D) Box plots show the relative content of glycosylated quercetin‐3*‐O‐*glucosidie in A and G genotypes. Q3G, quercetin‐3*‐O‐*glucosidie. The content was log2 transformed, **P < 0.01, Student's t‐test. E) Box plots show the ratio between glycosylated quercetin and quercetin‐3*‐O‐*glucosidie in A and G genotypes. The content was log2 transformed, **P < 0.01, Student's *t*‐test. F) A diagram illustrating the simulation docking of FtUFGT3 with quercetin. G) Enzymatic assay for quercetin of FtUFGT3 in vitro. H) Content of quercetin‐3*‐O‐*glucoside in three *FtUFGT3‐OE* hairy roots lines. DW, Dry Weight. Data show the arithmetic mean ± SD from 3 biological replicates. **P < 0.01, Student's *t*‐test.

In our previous research, GWAS analysis identified a significant SNP (Ft1: 4617722), located in the promoter of *FtUFGT3*, associated with the content of quercetin‐3*‐O‐*glucoside in Tartary buckwheat germplasm resources.^[^ ^33^, ^34^ ^]^ (**Figure** **1**A; Figure S1, Supporting Information). Similarly, through GWAS, the contents of substances such as kaempferol‐3‐*O*‐glucoside‐7‐*O*‐rhamnoside and kaempferol‐3‐*O*‐flavonoid glucoside, were found closely associated with this SNP (Figures S2–S4, Supporting Information), suggesting that FtUFGT3 may influence the synthesis of multiple flavonoid compounds. Further analysis revealed that this locus underwent selection during Tartary buckwheat domestication based on cross‐population composite likelihood ratio (XP‐CLR) and *F*_st_ analysis (Figure 1B). Previous studies have shown that FtUFGT3 could also glycosylate cyanidin and kaempferol.^[^ ^33^, ^35^ ^]^.To investigate whether FtUFGT3 could glycosylate other flavonoids, we next analyzed the metabolite content in accessions with different genotype based on this SNP.^[^ ^31^ ^]^ The results showed that accessions harboring the G‐genotype exhibited a higher ratio of quercetin‐3‐*O*‐glucoside to quercetin compared to those harboring A‐genotype (Figure 1C–E). A similar result was also found in the ratio of myricetin‐3‐*O*‐glucoside to myricetin (Figure S5, Supporting Information). As previous research illustrated the expression of *FtUFGT3* was significantly higher in A‐genotype than in the G‐haplotype.^[^ ^33^ ^]^ we speculated that FtUFGT3 might also participate in the glycosylation of quercetin and myricetin.

To verify the substrates of FtUFGT3, molecular docking simulations were performed with these candidate substrates. The results showed that quercetin (Figure 1F; Figure S6, Supporting Information), kaempferol (Figures S7 and S8, Supporting Information), myricetin (Figures S9 and S10, Supporting Information), and UDP‐glucose (Figure S11, Supporting Information) exhibited relatively high binding efficiency. Therefore, we hypothesize that FtUFGT3 can catalyze the glycosylation of not only kaempferol but also quercetin and myricetin. To verify this hypothesis, FtUFGT3‐MBP protein was obtained through prokaryotic expression (Figure S12, Supporting Information). Using quercetin, kaempferol, and myricetin as substrates, the in vitro enzymatic activity of FtUFGT3 was measured. The results showed that FtUFGT3 could catalyze the glycosylation of quercetin (Figure 1G), kaempferol (Figure S13 Supporting Information), and myricetin (Figure S14, Supporting Information). The kinetic parameters indicate that FtUFGT3 has the highest catalytic activity toward kaempferol (Figure S15, Supporting Information). To verify the function of FtUFGT3 in vivo, FtUFGT3 overexpressing Tartary buckwheat hairy roots were constructed (Figure S16, Supporting Information). The results showed that *FtUFGT3* overexpression significantly increased the content of quercetin‐3‐*O*‐glucoside and kaempferol‐3‐*O*‐glucoside in Tartary buckwheat hairy roots (Figure 1H; Figure S17, Supporting Information), indicating that FtUFGT3 plays a significant role in the glycosylation of quercetin and kaempferol in Tartary buckwheat. These results demonstrated that FtUFGT3 exhibits catalytic activity toward multiple substrates in flavonoid biosynthesis, highlighting its indispensable role in flavonoid metabolism.

### 2.2 The FtMADS1 Transcription Factor Directly Binds to the Promoter of *FtUFGT3* and Suppresses Its Expression

**Figure 2:**
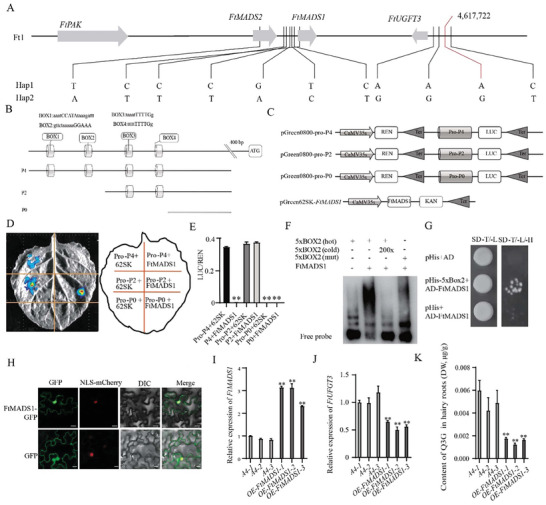
FtMADS1 inhibits *FtUFGT3* expression through directly binding to the promoter of *FtUFGT3*. A) The haplotype of gene cluster *FtPAK‐FtMADS1/2‐FtUFGT3*. B) A diagram showing the distribution of MADS‐box elements on the upstream 2000 bp region of the *FtUFGT3* promoter and the promoter fragments used in this study. C) Schematic diagram of the recombinant vector in the transcription activation experiment. D) The results of dual bioluminescence assay. The fusion proteins were expressed in *N‐benthamiana* using agroinfiltration. Chemiluminescence images were captured 36 h after infiltration using 3 mg mL^−1^ luciferin. Similar results were obtained in 3 biological replicates. E) The ratio of the luciferase (LUC) activity and the recombinant enzyme activity of the reference gene (renilla, REN) of D). Data show the arithmetic mean ± SD from 3 biological replicates. **P < 0.01, Student's *t*‐test. F) Results of the EMSA for the interaction between FtMADS1 and 5×BOX2. G) Y2H results of FtMADS1 and FtMADS2. SD‐L/‐T, the SD basic culture medium lacked Leu and Trp. SD‐L/‐T/‐H/‐A, SD basal medium lacked Leu, Trp, His, and Ade, and added 10 mm3‐AT. H)Subcellular localization of FtMADS1. FtMADS1‐GFP, pCAMBIA1300‐FtMADS1 recombination plasmid; NLS‐mCherry, nuclear marker; DIC, differential interference contrast ; Merge, merge channel. Bar = 25 µm. I) The relative expression level of *FtMADS1* in three *OE‐FtMADS1* hairy root lines. Data show the arithmetic mean ± SD from 3 biological replicates. **P < 0.01, Student's t‐test. J) The relative expression level of *FtUFGT3* in three *OE‐FtMADS1* hairy root lines. Data show the arithmetic mean ± SD from 3 biological replicates. **P < 0.01, Student's t‐test. K) Content of quercetin‐3*‐O‐*glucoside in three *OE‐FtMADS1* hairy roots lines. Data show the arithmetic mean ± SD from 3 biological replicates. **P < 0.01, Student's *t*‐test.

Given that the SNP Ft1: 4617722, shows a significant correlation with the differences in *FtUFGT3* expression and kaempferol‐3‐*O*‐glucoside content^[^ ^33^ ^]^ ，we further analyzed the SNPs of adjacent genes linked to this SNP to investigate whether there are other genes in the nearby region that can affect FtUFGT3 expression. Sequence analysis illustrated that three other SNPs located in *FtUFGT3*, six SNPs located in *FtMADS1* promoter, and one SNP located in *FtMADS2* promoter， all of which were linked to the lead SNP (Ft1: 4617722; **Figure** **2**A). Further analysis revealed that the upstream ‐2000 bp sequence of the *FtUFGT3* promoter contains four MADS boxes, namely BOX1, BOX2, BOX3, and BOX4 (Figure 2B), and these two genes also exhibited relatively high expression during seed development, similar to *FtUFGT3*.^[^ ^33^ ^]^ In addition, it has been reported that MADS‐box transcription factors could be phosphorylated^[^ ^36^, ^37^ ^]^, and a gene encoding protein kinase, FtPAK, was found located upstream of *FtMADS2*. Therefore, we hypothesized that there might be regulatory relationships between *FtPAK*, *FtMADS1/2*, and *FtUFGT3*.

To investigate whether FtMADS1 and FtMADS2 could bind to *FtUFGT3* promoter, three fragments of *FtUFGT3* promoter were obtained and constructed into 62SK vector (Figure 2B). Fragment P4 (−2000–−200 bp) contained four MADS‐boxes, fragment P2(−1400–−200 bp) contained two MADS‐boxes closer to the starting codon, while fragment P0 (−600–−200 bp) contained no MADS‐boxes. Dual bioluminescence assay was further conducted in tobacco leaves. The results showed that 62SK+P4 and 62SK+P2 have luciferase activity, while 62SK+P0 could not, indicating that P4 and P2 alone have transcriptional activity (Figure 2C–E). Furthermore, compared to 62SK+P4, FtMADS1+P4 did not produce any luciferase activity, indicating that FtMADS1 can inhibit the transcriptional activity of P4. In comparison to 62SK+P2, FtMADS1+P2 still has luciferase activity, indicating that FtMADS1 does not affect the transcriptional activity of P2. Similarly, compared to 62SK+P0, FtMADS1+P0 still did not show luciferase activity, indicating that FtMADS1 does not affect the transcriptional activity of P0. These results indicated that FtMADS1 could inhibit the transcriptional activity of the *FtUFGT3* promoter within BOX1 or BOX2 (Figure 2C–E). Furthermore, electrophoretic mobility shift assay (EMSA) demonstrated that FtMADS1 could directly bind to the DNA fragment of 5 × BOX2 (Figure 2F) but not 5 × BOX1 (Figure S18, Supporting Information). When the core bases of BOX2 were mutated, the binding of FtMADS1 to DNA was reduced. In addition, EMSA results also revealed that FtMADS1 exhibited no binding ability to 5 × BOX1 in vitro, indicating that FtMADS1 could directly bind to BOX2 in the promoter of *FtUFGT3*. Yeast one‐hybrid assay revealed that yeast cells co‐transformed with AD‐FtMADS1 and 5 × BOX2‐pHis were able to grow normally on SD‐T/‐L‐/H medium, whereas the control groups could not, confirming that FtMADS1 could bind to BOX2 in vivo (Figure 2G). Subcellular localization revealed that FtMADS1 was located both in the nucleus and the cytoplasm, with its primary location being in the nucleus (Figure 2H). In order to investigate the effect of FtMADS1 on *FtUFGT3* expression in vivo, *FtMADS1* overexpressing Tartary buckwheat hairy roots were constructed (Figure 2I). The expression level of *FtUFGT3* was significantly down‐regulated in all three lines of *FtMADS1* overexpressing hairy roots (Figure 2J). Moreover, the content of glycosylated quercetin was significantly reduced (Figure 2K), which was in accordance with the suppression of transcriptional activity of FtMADS1 on *FtUFGT3* and the function of FtUFGT3 in catalyzing quercetin glycosylation. These results indicated that FtMADS1 could inhibit the expression of *FtUFGT3* through directly binding to the MADS‐BOX2 in the promoter of *FtUFGT3*, and thereby suppress quercetin glycosylation in Tartary buckwheat.

### 2.3 The Interaction Between FtMADS2 and FtMADS1 Alleviates the Inhibitory Effect of FtMADS1 on *FtUFGT3* Expression

**Figure 3:**
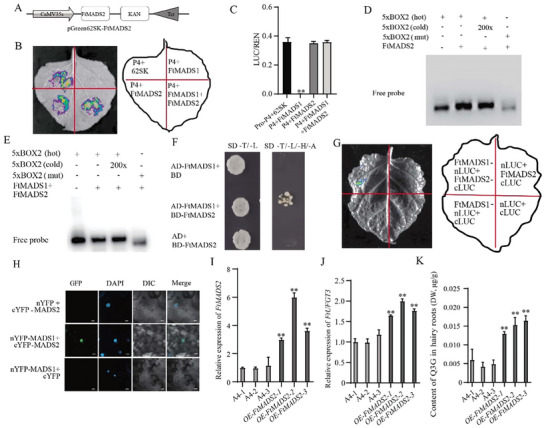
The interaction between FtMADS2 and FtMADS1 alleviates the inhibitory effect of FtMADS1 on *FtUFGT3* expression.A) Schematic diagram of the recombinant vector in the dual bioluminescence assay. B) The results of dual bioluminescence assay. The fusion proteins were expressed in *N‐benthamiana* using agroinfiltration. Chemiluminescence images were captured 36 h after infiltration using 3 mg mL^−1^ luciferin. Similar results were obtained in 3 biological replicates. C) The ratio of the luciferase (LUC) activity and the recombinant enzyme activity of the reference gene renilla (REN) of B. Data show the arithmetic mean ± SD from 3 biological replicates. **P < 0.01, Student's *t*‐test. D) Results of the EMSA for the interaction between FtMADS2 and 5×BOX2. E) Results of the EMSA for the interaction between FtMADS1+FtMADS2 and 5×BOX2. F) The yeast two‐hybrid (Y2H) results of the interaction of FtMADS1 and FtMADS2. SD‐L/‐T, SD basic medium lacked the Leu and Trp; SD‐L/‐T/‐H/‐A, SD basal medium lacked Leu, Trp,His and Ade, and contained 50 mM 3‐AT. G) The interaction between FtMADS1 and FtMADS2 was confirmed using LCA. The N‐ or C‐terminal fragment of LUC (nLUC or cLUC) was fused with their respective proteins. The experiment was performed according to the grouping shown in the figure. The constructed fusion proteins were co‐expressed in *N.benthamiana* using agroinfiltration. Images of chemiluminescence were recorded by applying 3 mg mL^−1^ luciferin 36 h after infiltration. Similar results were obtained in 3 biological replicates. H) The BiFC assay demonstrates interactions between FtMADS1 and FtMADS2 in the leaf epidermal cells of *N. benthamiana*. The N‐ or C‐terminal fragment of YFP (nYFP or cYFP) was fused with their respective proteins. The experiment was performed according to the grouping shown in the figure. The constructed fusion proteins were co‐expressed in *N.benthamiana* using agroinfiltration. 4′,6‐diamidino‐2‐phenylindole (DAPI) serves as a reliable marker for cellular nuclei. The results were observed through confocal microscopy after 48 h. The scale bar represents 25 µm. I) The relative expression level of *FtMADS2* in three *OE‐FtMADS2* hairy root lines. Data show the arithmetic mean ± SD from 3 biological replicates. **P < 0.01, Student's t test. J) The relative expression level of *FtUFGT3* in three *OE‐FtMADS2* hairy root lines. Data show the arithmetic mean ± SD from 3 biological replicates. **P < 0.01, Student's t test. K) Content of quercetin‐3*‐O‐*glucoside in three *OE‐FtMADS2* hairy roots lines. Data show the arithmetic mean ± SD from 3 biological replicates. **P < 0.01, Student's *t*‐test.

To investigate whether FtMADS2 could regulate the expression of *FtUFGT3* by directly binding to the *FtUFGT3* promoter, similar to FtMADS1, dual bioluminescence assay was further performed. The results showed that similar to 62SK+P4, FtMADS2+P4 still has luciferase activity, indicating that FtMADS2 could not affect the transcriptional activity of P4 (**Figure** **3**B). Additionally, compared to FtMADS1+P4, FtMADS2+FtMADS1+P4 showed luciferase activity, indicating that FtMADS2 alleviated the transcriptional suppression effect of FtMADS1 on FtUFGT3 (Figure 3A–C). Furthermore, EMSA results indicated that FtMADS2 alone cannot bind to BOX2 on the promoter of *FtUFGT3* (Figure 3D). However, in the presence of FtMADS2, FtMADS1 no longer binds to the *FtUFGT3* promoter (Figure 3E). These results collectively suggest that although FtMADS2 cannot directly regulate the activity of *FtUFGT3* promoter, it can relieve the inhibition effect of FtMADS1 on *FtUFGT3* expression.

To explore the mechanism by which FtMADS2 relieves the inhibitory effect of FtMADS1 on *FtUFGT3* expression, we next investigated whether FtMADS2 could interact with FtMADS1. Y2HGold revealed that the yeast strain co‐transformed with FtMADS1 and FtMAD2 was able to grow normally on SD‐T/‐L‐/H/‐A medium, while the control group could not (Figure 3F). Subsequently, luciferase complementation assay revealed strong luciferase activity on the co‐transformation of FtMADS1‐nLUC with FtMAD2‐cLUC, but no bioluminescence was observed in the negative controls (Figure 3G,H). Bimolecular fluorescent complimentary (BiFC) assay also showed strong fluorescence in the nucleus but no fluorescence was observed in the negative controls (Figure 3I). These results collectively indicated that FtMADS2 alleviated the transcription suppression effect of FtMADS1 via its interaction with FtMADS1. In order to investigate the function of FtMADS2 in vivo, *FtMADS2* overexpressing Tartary buckwheat hairy roots were constructed (Figure 3J). The expression of *FtUFGT3* was significantly upregulated (Figure 3K), which was in contrast with the function of *FtMADS1*, indicating that *FtMADS2* can positively regulate the expression of *FtUFGT3* in Tartary buckwheat. Accordingly, the content of quercetin‐3*‐O‐*glucoside was significantly upregulated compared to the empty vector controls (Figure 3L). In summary, these results demonstrated that the interaction between FtMADS2 and FtMADS1 alleviates the transcription inhibition effect of FtMADS1 on *FtUFGT3* expression.

### 2.4 The Interaction Between FtPAK and FtMADS2 Enhances the Inhibitory Effect of FtMADS1 on *UFGT3* Expression

**Figure 4:**
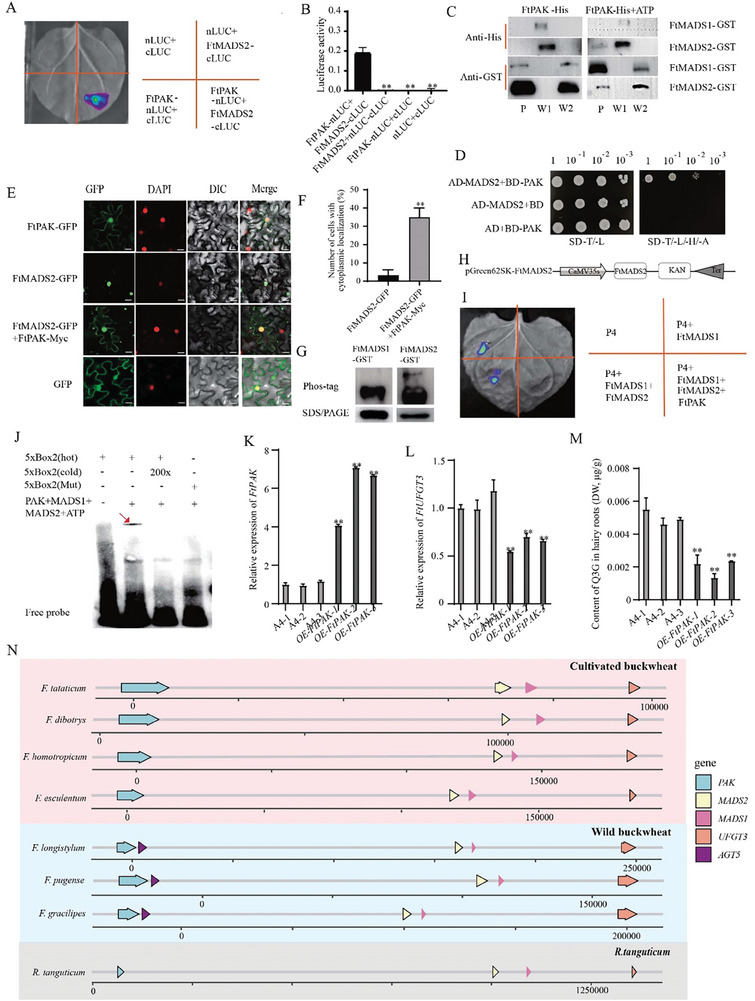
The interaction between FtPAK and FtMADS2 enhances the inhibitory effect of FtMADS1 on UFGT3. A) The interaction between FtPAK and FtMADS2 was confirmed using LCA. The N‐ or C‐terminal fragment of LUC (nLUC or cLUC) was fused with their respective proteins. The experiment was performed according to the grouping shown in the figure. The constructed fusion proteins were co‐expressed in *N‐benthamiana* using agroinfiltration. Images of chemiluminescence were recorded by applying 3 mg mL^−1^ luciferin 36 h after infiltration. Similar results were obtained in 3 biological replicates. B) The results of the pull‐down assay to detect the interaction between FtPAK and FtMADS1 and FtMADS2. In the experiment, recombinant proteins FtPAK‐His, FtMADS1‐GST, and FtMADS2‐GST were obtained through prokaryotic expression. As shown in Figure, FtPAK‐His was co‐incubated with FtMADS1‐GST or FtMADS2‐GST. GST‐tagged beads were used for the pull‐down assay and anti‐His was used for western blot detection. P represents Beads protein elution solution, W1 represents the first beads wash solution, W2 represents the final beads wash solution, and Anti‐GST and Anti‐His represent the antibodies used for western blot. C) Y2H results of FtMADS1 and FtMADS2. SD‐L/‐T, the SD basic culture medium lacked Leu and Trp. SD‐L/‐T/‐H/‐A, SD basal medium lacked Leu, Trp, His and Ade, and added 10 mm3‐AT. D) Subcellular localization of FtMADS2 and FtAPK. FtMADS2‐GFP, pCAMBIA1300‐FtMADS2 recombination plasmid. FtPAK‐GFP, pCAMBIA1300‐FtPAK recombination plasmid; FtPAK‐Myc, pCAMBIA1307‐FtPAK recombination plasmid. NLS‐mCherry, nuclear marker; Bar = 25 µm. E) The percentage of cells with cytoplasmic localization out of the total number of cells. 20 cells were counted in each measurement, and the experiment was independently repeated three times. Data are mean ± SD. **P < 0.01, Student's t test. F) The Phostag experiment validates the phosphorylation of FtMADS1/2 by FtPAK. G) Schematic diagram of the recombinant vector in the dual bioluminescence assay. H) The results of dual bioluminescence assay. The fusion proteins were expressed in *N. benthamiana* using agroinfiltration. Chemiluminescence images were captured 36 h after infiltration using 3 mg mL^−1^ luciferin. Similar results were obtained in 3 biological replicates I) FtPAK can phosphorylate FtMADS2, but cannot phosphorylate FtMADS1. In the presence of ATP, FtPAK is co‐incubated with FtMADS1 or FtMADS2, and analyzed by Phos‐tag mobility shift assays. SDS/PAGE is used as a control. J) Results of the EMSA for the interaction between FtMADS1+FtMADS2+FtPAK and 5×BOX2. FtMADS1+FtMADS2+FtPAK serves as the target protein. ATP is added and co‐incubated with the protein. The red arrows indicate the binding bands. K) The relative expression level of FtPAK in three *OE‐FtPAK* hairy root lines. Data show the arithmetic mean ± SD from 3 biological replicates. **P < 0.01, Student's *t*‐test. L) The relative expression level of *FtUFGT3* in three *OE‐FtPAK* hairy root lines. Data show the arithmetic mean ± SD from 3 biological replicates. **P < 0.01, Student's *t*‐test. M) Content of quercetin‐3*‐O‐*glucoside in three *OE‐FtPAK* hairy roots lines. Data show the arithmetic mean ± SD from 3 biological replicates. **P < 0.01, Student's *t* test. N) Distribution of the *UFGT3* gene cluster in *Fagopyrum* plants and *R. tanguticum*. The pink area represents cultivated buckwheat, the blue area represents wild buckwheat, and the gray area represents *R. tanguticum*. The line segments and numbers indicate the genome length.

Previous research illustrated that phosphorylation of MADS transcription factors is crucial for their function, and this phosphorylation process is dependent on the interaction with protein kinases.^[^ ^36^, ^37^ ^]^ We found that within the 100 kb up‐ and down‐stream regions of the lead SNP (Ft1: 4617722), a gene encoding protein kinase (FtPAK) was also highly expressed during Tartary buckwheat seed development.^[^ ^33^ ^]^ which was in accoradance with *FtUFGT3*, *FtMADS1* and *FtMADS2*. To examine whether this protein kinase can interact with FtMADSs, a luciferase complementation assay was further conducted. Live imaging showed a distinct luciferase activity when FtPAK‐nLUC and FtMAD2‐cLUC were co‐present (**Figure** **4**A,B). Subsequently, recombinant proteins of FtPAK‐His, FtMADS1‐GST, and FtMADS2‐GST were obtained in order to verify their interactions via pull‐down analyses. The results indicated that FtPAK could interact with FtMADS2 but not FtMADS1, and this interaction requires the presence of ATP (Figure 4C). Results of Y2H analyses indicated that the yeast strain co‐transformed with FtPAK and FtMADS2 was able to grow on SD‐T/‐L‐/H/‐A medium, while the negative controls could not (Figure 4D). Subcellular localization experiments demonstrated that FtPAK is localized in the nucleus and cytoplasm, while FtMADS2 is solely localized in the nucleus (Figure 4E). However, when tobacco cells co‐transformed with FtMADS2 and FtPAK, the subcellular location of FtMADS2 changed. The number of cells with FtMADS2 cytoplasm‐located was increased in FtMADS2 and FtPAK co‐transformed, compared to that transformed with FtMADS2 alone (Figure 4E,F). To investigate whether this interaction could result in FtMADS2 phosphorylation, Phostag experiments were further performed and showed that FtMADS2 could be phosphorylated by FtPAK (Figure 4G), indicating that FtPAK can phosphorylate FtMADS2 and induce FtMADS2 translocation from the nucleus to the cytoplasm.

To investigate whether the interaction between FtPAK and FtMADS2 could alter the function of FtMADS2, a dual bioluminescence assay was further conducted. Results revealed that FtPAK alleviated the inhibition of FtMADS2 on FtMADS1 transcription suppression activity (Figure 4H,I). Furthermore, EMSA results demonstrated that in the presence of both FtPAK and FtMADS2, FtMADS1 could bind to BOX2 (Figure 4J), indicating that the interaction between FtPAK and FtMADS2 could enhance the transcription inhibition effect of FtMADS1 on *FtUFGT3*. To investigate the in vivo function of FtPAK, *FtPAK* overexpressing Tartary buckwheat hairy roots were further constructed (Figure 4K). The expression of Ft*UFGT3* and the content of quercetin‐3*‐O‐*glucoside were significantly reduced in overexpressing hairy roots (Figure 4L,M), further confirming that this non‐homologous gene cluster synergistically regulates the glycosylation of quercetin. After investigating the interplay among the four genes *FtPAK*, *FtMADS1*, *FtMADS2*, and *FtUFGT3*, we have tentatively ascertained that in Tartary buckwheat, these genes are not randomly organized but rather form an integrated molecular regulatory module that collectively regulates flavonoid biosynthesis.

### 2.5 The Biosynthetic Function and Molecular Regulatory Pattern of the *UFGT3* Gene Cluster are Conserved within the *Fagopyrum* Genus

To investigate whether this gene cluster was also present in other species of the *Fagopyrum* genus, we analyzed the sequences of the genomes of *F. dibotrys*,^[^ ^38^ ^]^ *F. esculentum*,^[^ ^39^ ^]^ *F. homotropicum*,^[^ ^40^ ^]^ *F. longistylum*, *F. pugense*, *F. gracilipes*, and *R. tanguticum*.^[^ ^41^ ^]^ It was found that although the gene spacing varies between different species, this gene cluster exists in all of the above‐mentioned species (Figure 4N), implying that the function of *UFGT3* cluster was conserved. To verify this hypothesis, the following experiments were subsequently performed. The molecular docking simulation results showed that within each of the seven species, UFGT3 has similar catalytic activity for cyanidin, quercetin, and myricetin (Figures S20–S34, Supporting Information), indicating that the function of UFGT3 is highly conserved. Dual bioluminescence assays further revealed that in seven species, the expression of *UFGT3* is inhibited by MADS1 (Figures S35–S37, Supporting Information). Yeast two‐hybrid assay further showed that PAK can interact with MADS2 (Figures S38–S40, Supporting Information) MADS1 in *Fagopyrum* genus (Figure S41, Supporting Information). These results indicate that the function and the molecular regulatory pattern of *UFGT3* gene cluster are highly conserved within the *Fagopyrum* genus.

### 2.6 The *UFGT3* BGC Has Undergone Adaptive Evolution Between Wild Relatives and Cultivated Buckwheat

**Figure 5:**
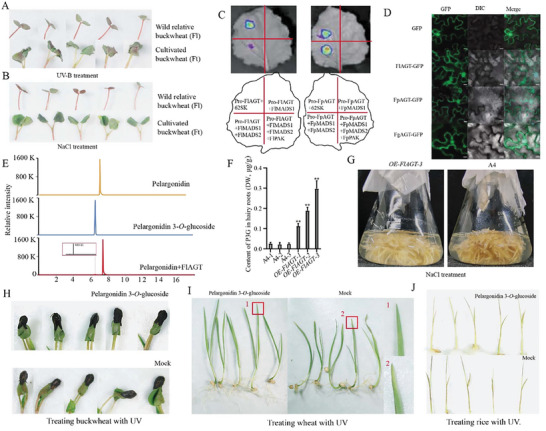
AGT contributes to the high‐altitude adaptability of wild buckwheat. A) Wild buckwheat is more resistant to UV stress than cultivated buckwheat. Fl represents *F. longistylum*, Ft represents *F. tataticum*. B) Wild buckwheat is more resistant to NaCl stress than cultivated buckwheat. C) The results of dual‐luciferase assay. The fusion proteins were expressed in *N. benthamiana* using agroinfiltration. Chemiluminescence images were captured 36 h after infiltration using 3 mg mL^−1^ luciferin. Similar results were obtained in three biological replicates. D) Subcellular localization of FlAGT, FpAGT, and FgAGT. FlAGT‐GFP, pCAMBIA1300‐*FlAGT* recombinant plasmid. FpAGT‐GFP, pCAMBIA1300‐*FpAGT* recombinant plasmid; FgAGT‐GFP, pCAMBIA1300‐*FgAGT* recombinant plasmid. Bar = 10 µm. E) Enzymatic assay for pelargonidin of FtAGT in vitro. The red boxes represent the enlarged areas. F) Detection of pelargonidin 3*‐O‐*glucoside expression in the overexpressed *FlAGT* hairy roots; P3G represents pelargonidin 3*‐O‐*glucoside. G) Phenotype of the overexpressed *FlAGT* hairy roots treated with 100 mM NaCl. H) Spraying pelargonidin 3*‐O‐*glucoside on the Tartary buckwheat seedlings, water was sprayed in the mock group. UV‐B irradiation caused rust spots on the leaves. I) Spraying pelargonidin 3*‐O‐*glucoside on the leaves of wheat, water was sprayed in the mock group. UV‐B irradiation caused rust spots on the leaves, and the red boxes with numbers represent the enlarged areas corresponding to the numbers on the right. J) Spraying pelargonidin 3*‐O‐*glucoside on the rice seedlings, water was sprayed in the mock group. UV‐B irradiation caused rust spots on the seedlings.

It is worth noting that, compared to cultivated buckwheat (*F. dibotrys*, *F. esculentum*, *F. homotropicum*) and *R. tanguticum* of the *Polygonaceae*, a gene encoding anthocyanin glucosyltransferase (AGT) is inserted between *PAK* and *MADS2* in wild relatives of buckwheat (*F. longistylum*, *F. pugnse*, *F. gracilipes*) (Figure 4N). Compared to cultivated buckwheat, wild relatives of Tartary buckwheat exhibit stronger high‐altitude adaptability. The results of the UV‐B and salt resistance tests also support this point. As shown in **Figure** **5**A,B, after UV‐B and NaCl treatment, compared to *F. longistylum*, the leaves of Tartary buckwheat displayed curling and wilting, indicating that wild relatives have stronger resistance to UV and NaCl. Therefore, we hypothesize that the *AGT* gene inserted in the UFGT3 BGC gives the wild relatives stronger high‐altitude adaptability. We further investigate whether this gene was also regulated by UFGT3 cluster. The dual‐luciferase reporter assay further showed that MADS1 can inhibit *AGT* expression (Figure 5C). Further research revealed that in the presence of both MADS2 and MADS1, the expression of *AGT* is no longer inhibited (Figure 5C), indicating that the interaction between MADS2 and MADS1 can relieve the inhibition effect of MADS1 on *AGT*. Furthermore, when PAK and MADS1/2 are simultaneously present, the inhibition effect of MADS1 on *AGT* expression is reinstated (Figure 5C). This result indicates that PAK phosphorylated MADS2 disrupts the interaction between MADS1 and MADS2, allowing MADS1 to inhibit *AGT* expression.

The function of AGT was further analyzed. Subcellular localization showed that FlAGT and FpAGT are located in both the cytoplasm and nucleus (Figure 5D). As AGT is an anthocyanin glucosyltransferase, we tested the molecular docking of FlAGT with several major anthocyanins (cyanidin, petunidol, pelargonidin, and delphinidin). The results showed that FlAGT exhibited varying degrees of catalytic activity toward them (Figures S42 and S45, Supporting Information). In vitro enzyme activity experiments revealed that FlAGT could only glycosylate pelargonidin into pelargonidin‐3*‐O‐*glucoside (Figure 5E), but could not glycosylate the other three substances. Overexpression of *FlAGT* in hairy roots significantly increased the content of pelargonidin 3*‐O‐*glucoside (Figure S46, Supporting Information; Figure 5F), further confirming the function of *FlAGT*. It is worth noting that after UV‐B, there was a significant upregulation of pelargonidin‐3‐*O*‐glucoside content in the *F. longistylum*, while there was no significant difference compared to untreated buckwheat (Figure S47, Supporting Information).

We investigated the protective effects of pelargonidin‐3‐*O*‐glucoside on buckwheat and other crops against UV‐B damage. Measurement of pelargonidin‐3‐*O*‐glucoside levels revealed a significant increase in treated buckwheat, indicating its effective entry into the plant through the surface (Figure S48, Supporting Information). UV‐B treatment combined with diaminobenzidine (DAB) staining visualized damage in plants treated with pelargonidin‐3‐*O*‐glucoside, showing a notable reduction in rust disease on the young leaves of Tartary buckwheat compared to controls (Figure 5H; Figures S52B, Supporting Information). Additionally, symptoms of rust were diminished on young wheat leaves, and root growth was healthy (Figure 5I; Figure S51A, Supporting Information). In rice, surface damage on seedlings was also reduced compared to controls (Figure 5J). Similar protective effects were observed in barley and *F. homotropicum*, as shown in Figures S49 and S50 (Supporting Information), with visual results in Figure S51B,C (Supporting Information). In the field experiment conducted at an altitude of 3500 m, three widely cultivated varieties of Tartary buckwheat (ZK3, CQ1, CQ2) were subjected to foliar spraying with pelargonidin 3*‐O‐*glucoside. The results revealed that the treated varieties exhibited an increase in both plant height and grain number (Figures S53 and S54, Supporting Information). These findings suggest that pelargonidin 3*‐O‐*glucoside universally enhances plant resistance to ultraviolet stress and holds significant developmental and utilitarian value.

**Figure 6:**
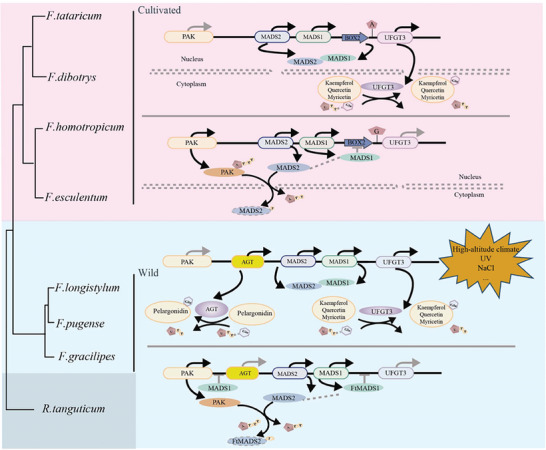
Molecular pattern schematics of *UFGT3* BGC in cultivated and wild relatives of buckwheat. In cultivated buckwheat, when the expression level of *PAK* is low, MADS2 can interact with MADS1, alleviating the transcriptional inhibitory effect of MADS1 on the expression of *UFGT3*, thus promoting the glycosylation activity of *UFGT3* on its substrates. However, when the expression level of *PAK* is high, PAK can phosphorylate MADS2, leading to the translocation of MADS2 from the nucleus to the cytoplasm. This means that MADS2 can no longer interact with MADS1, causing the MADS1 transcription factor to bind to the MADS‐BOX2 element on the *UFGT3* promoter, thereby inhibiting *UFGT3* expression and weakening its glycosylation activity on its substrates. Similarly, the complex molecular regulatory mechanism of the *UFGT3* gene cluster in cultivated buckwheat also presents the same conservation in wild relatives. The difference is that in the wild relatives, an *AGT* gene is inserted between PAK and MADS2. AGT can glycosylate pelargonidin and is regulated in the same way as PAK, MADS1/2, similar to the regulation pattern of *UFGT3*. The additional *AGT* enables the *UFGT3* gene cluster to have a more diversified synthesis and regulatory capability of flavonoids, endowing wild buckwheat relatives with a stronger adaptability to high‐altitude climates.

In conclusion, a model can be formulated to describe the regulatory patterns of the *UFGT3* BGC in cultivated buckwheat and wild relatives (**Figure** **6**). In cultivated buckwheat, when the expression level of *PAK* is low, MADS2 can interact with MADS1, alleviating the transcriptional inhibitory effect of FtMADS1 on the expression of *FtUFGT3*, thus promoting the glycosylation of its substrates. However, when the expression level of *PAK* is high, PAK can interact with phosphate MADS2, leading to the translocation of MADS2 from the nucleus to the cytoplasm. This means that MADS2 can no longer interact with MADS1, causing the MADS1 transcription factor to bind to the MADS‐BOX2 element on the *UFGT3* promoter, thereby inhibiting *UFGT3* expression and weakening its glycosylation activity on its substrates. Although the complex molecular regulatory mechanism of the *UFGT3* BGC in cultivated buckwheat is mainly conserved in wild relatives, an *AGT* gene is inserted between *PAK* and MADS2. AGT can glycosylate pelargonidin and is regulated in the same way as PAK, MADS1/2, similar to the regulation pattern of *UFGT3*. The addition of *AGT* enables the *UFGT3* gene cluster to have a more diversified synthesis and regulatory capability of flavonoids, endowing wild buckwheat relatives with a stronger adaptability to high‐altitude climates.

## 3 Discussion

Genes relevant to plant secondary metabolism often exist in the form of BGCs in the genome. Studying BGCs can deepen our understanding of the biosynthetic mechanisms of metabolites. Their formation, domestication, and differentiation can significantly impact the stress resistance and ecological adaptability of plants. However, despite the increasing revelation of plant BGCs, the lack of genomic data has resulted in insufficient evolutionary dynamics analysis of BGCs within and between species.^[^ ^31^ ^]^ In our study, we identified a glycosyltransferase gene, UFGT3, through GWAS and metabolomics data. By examining SNPs linked to nearby genes, we discovered and characterized a molecular module‐type biosynthetic gene cluster (BGC), the UFGT3 gene cluster, in buckwheat. Comparative genomic analysis of seven Fagopyrum species (including both cultivated and wild relatives) revealed that this gene cluster is not only widely present within the genus but also influenced by domestication (Figures 1B and 2A) and evolved under environmental pressures (Figure 4N).

Intraspecifically, SNPs within BGCs often have significant impacts on the biosynthesis of metabolites. SNPs and genome‐associated insertion/deletion events have been linked to the maintenance and diversification of BGCs in fungi.^[^ ^42^ ^]^ In *Arabidopsis thaliana*, SNPs and small insertion/deletions are the most common sequence polymorphisms in the tryptophanol BGC.^[^ ^43^ ^]^ A survey of haplotypes in the rice japonica and indica subtypes, along with *Oryza rufipogon Griff*., showed that the intact rice diterpenoid gene cluster on chromosome 7 (*DGC7*), which encodes the entire biosynthetic pathway to 5,10‐diketo‐casbene, is enriched in japonica varieties compared to indica ones, suggesting that *DGC7* has undergone selection during domestication.^[^ ^44^ ^]^ In our study, *UFGT3* BGC was uncovered to have been domesticated and selected during the transition from wild to cultivated Tartary buckwheat (Figures 1B and 2A).

BGCs may either be conservative or have undergone convergent evolution or divergent differentiation between species. The monoterpene indole alkaloid (MIA) BGC in *Ophiorrhiza pumila* shows homology with the regions identified in *Gelsemium sempervirens* and *Catharanthus roseus*. In contrast, *Coffea canephora* lacks strictosidine synthase in this region, which may lead to the absence of MIAs in this species.^[^ ^45^ ^]^ Our study demonstrates that the relative position and co‐regulation among members of the *UFGT3* BGC within the *Fagopyrum* genus are conservative. Unlike cultivated Tartary buckwheat, this gene cluster has an anthocyanin glucosyltransferase *AGT* inserted between *PAK* and *MADS2* in wild relatives. AGT enzyme could glycosylate pelargonidin (Figure 4N), and its expression is also regulated by PAK, MADS2, and MADS1 as UFGT3 (Figure 5C). The formation of BGCs is closely related to environmental adaptability. The greater the specific environmental pressure (e.g., UV radiation, pest infestations, drought), the more likely plants are to form certain gene clusters to adapt to such pressures. This is because members within gene clusters form tight linkages, providing plants with more effective genetic strategies.^[^ ^46^ ^]^ For example, the β‐diketones, which are components of leaf surface wax and are polyketides encoded by a gene cluster, protect against pathogens and pests.^[^ ^47^ ^]^ We have demonstrated that glycosylated pelargonidin helps plants resist UV and high salt stress (Figure 6H–J; Figures S41–S44, Supporting Information). This appears to suggest that the expansion of this cluster in close wild relatives of Tartary buckwheat is beneficial for the plant's adaptation to high‐altitude environments, thus serving as a typical example of gene cluster structural change driving plant differentiation.

Gene clusters are common in bacteria and fungi and usually include genes encoding pathway‐specific transcription factors for the regulation of cluster biosynthesis‐specific genes.^[^ ^48^ ^]^ Loss‐of‐function mutations in these transcription factors lead to the loss of pathway gene expression, while overexpression can lead to coordinated upregulation and enhanced metabolite production.^[^ ^49^ ^]^ Interestingly, the plant BGCs reported thus far do not contain genes encoding pathway‐specific transcription factors.^[^ ^31^ ^]^ In our study, the *UFGT3* cluster, similar to some fungal gene clusters, contains not only a glycosyltransferase gene *UFGT3*, but also two transcription factors *MADS1* and *MADS2*. Furthermore, unlike other clusters, a protein kinase *PAK* that phosphorylates MADS2 is also present in the UFGT3 cluster. To our knowledge, a case of an intact BGC containing a complete molecular regulatory module such as *UFGT3* within a cluster has not been reported before. One possible reason is that current gene cluster studies tend to seek a few synthetic or modifying enzymes composed gene clusters, lacking related concepts and exploration methods for molecular module‐type gene clusters. Thus, understanding the basic structure of molecular module‐type gene clusters and developing corresponding identification strategies and tools will be the focus of future research given the increase in the publication of more genomes. Whilst not yet reported we believe that molecular module‐type gene clusters in plant species will ultimately prove not to be exceptions. Research on such molecular module‐type gene clusters will therefore likely greatly deepen our understanding of their formation and evolution and as such contribute to a more comprehensive understanding of genome structure and evolution.

## 4 Experimental Section

### Genome‐Wide Association Analysis

The resequencing data of 320 Tartary buckwheat accessions were obtained from published work. GWAS was conducted using the method previously illustrated by Zhang et al.^[^ ^33^ ^]^

### Subcellular Localization Assays

The full‐length cDNAs of *FtMADS1*, *FtMADS2*, and *FtPAK* were amplified and cloned into the pCAMBIA1300‐GFP vector. A marker for the nucleus, p2300‐35s‐H2B‐mCherry, was employed to label nuclei. The plasmid was introduced into *Nicotiana benthamiana* (*N. benthamiana*) leaves using transient infiltration mediated by *Agrobacterium tumefaciens* strain GV3101. After 48 h, subcellular localization was observed using a laser scanning confocal microscope (Zeiss LSM900) with excitation/emission wavelengths at 488/500–530 nm for GFP and 561/590‐640 nm for mCherry. The primer sequences are listed in Data Set 2 (Supporting Information).

### BiFC Analysis

The full‐length cDNAs of FtMADS1 and FtMADS2 were amplified and cloned into the pSPYNE‐35S and pSPYCE‐35S vectors, respectively. DAPI was used to label the cell nucleus. Constructs were introduced into *N. benthamiana* leaves through an infiltration mediated by *Agrobacterium* strain GV3101. After 48 h, fluorescent signals were observed using a laser scanning confocal microscope (Zeiss LSM900). Primer sequences are available in Data Set 2 (Supporting Information).

### Enzymatic Assay

The *FtUGT3* and *FlAGT* CDS were inserted into the pMAL‐C2X MBP fusion expression vector and transformed into *Escherichia coli* (*E. coli*) BL21. According to previous descriptions (see ref. [33]), the MBP fusion proteins were extracted and immobilized onto amylose beads (New England Biolabs) with protein extraction buffer, including 20 mM Tris‐HCl (pH 7.4), 0.2 M NaCl, and 1 mM EDTA. Protein was eluted using 20 mM Tris‐HCl (pH 7.4), 0.2 M NaCl, 1 mM EDTA, and 10 mM maltose. The reaction mixture (pH 8.0, 100 mM Tris‐HCl, 14 mM β‐mercaptoethanol, 9 mM UDP‐glucose, and 100 µM kaempferol, quercetin, myricetin, or pelargonidin was added to 5 µg purified protein and incubated at 37 °C for 30 min. The reaction was terminated by the freeze‐dryer at 40 °C. The dried reaction products were re‐dissolved in 80% methanol, and 5 µL of the solution was analyzed by HPLC (Agilent G6500 Series HPLC‐QTOF) to determine the product using the corresponding standards (Sigma–Aldrich, USA).

### Determination of Kinetic Parameters

The reaction system was set up as described in the enzyme activity assay. The substrate concentration ranges were as follows: 17.45–174.5 µM for kaempferol, 16.56–165.6 µM for quercetin, and 15.7–157 µM for myricetin. The amount of FtUFGT3 protein used was 5 µg, and the reactions were conducted at 30 °C for 30 min. After the reaction, 5 µL of the solution was analyzed by HPLC (Agilent G6500 Series HPLC‐QTOF) to determine the product using standards. The experimental results were processed using GraphPad software, and non‐linear regression was applied to fit the Michaelis–Menten equation to determine the kinetic parameters K_m_ and V_max_.

### Transgenic Hairy Roots

The CDS of *FtUGT3*, *FtPAK*, *FtMADS1 FtMADS2*, and *FlAGT* was inserted into the pCAMBIA‐1300 vector and transformed into Agrobacterium A4 to generate transgenic hairy roots following previous methods.^[^ ^18^ ^]^ Two weeks old sterile seedlings of Tartary buckwheat were cut and used as the explant for the infection with the Agrobacterium for 10 min. After the co‐culture on MS solid medium in the dark for 48 h at 25 °C, explants were washed by MS liquid medium containing 300 mg mL^−1^ cefotaxime and sterile water, and then cultured on MS solid medium containing 300 mg mL^−1^ cefotaxime in the growth chamber for the hairy root induction. The induced single hairyroot lines were removed from explants after 2 weeks and put on MS solid medium with 100 mg mL^−1^ cefotaxime for detoxification and growth. The positively transgenic lines were tested by PCR and moved to MS liquid medium with 100 mg mL^−1^ cefotaxime shaking for 2 weeks in the dark at 22 °C, 160 r min^−1^. For salt tolerance testing, *OE‐FlAGT*‐1, 2, and 3 lines with good growth on MS solid medium were transferred to liquid MS medium containing 100 mM NaCl. They were shaken as per the above method, and the phenotype was observed after two weeks. Hairy roots induced by A4 without the introduction of any vector were used as a control.

### UV‐B Treatment of Plants

Seed germination and seedling growth of all crops were conducted hydroponically. *Fagopyrum longistylum*, Tartary buckwheat (ZK3), wheat (ZM578), barley (HTX), and *F. homotropicum* were grown under a 16 h light/8 h dark cycle with 75% humidity. Rice (Japonica) seeds were germinated at 37 °C until sprouting and then transferred to the same cultivation conditions as the other crops. For UV light intervention experiments crops were exposed to 311 nm UV light (10 cm from the plants, 1.76 mw cm^−2^) until UV stress symptoms appeared in seedlings. The UV‐B irradiation experiments were conducted in a dark artificial climate chamber.

### Diaminobenzidine (DAB) Staining

DAB (1 mg mL^−1^) was dissolved in PBS buffer (pH 3.8) and mixed thoroughly. After the samples were rinsed with phosphate buffer solution (PBS), they were subsequently immersed in the DAB staining solution prior to being shaken horizontally at 40 rpm for 6 h. The degree of staining was observed following decolorization with 95% alcohol.

### Treatment of Plants with Flavonoids—Laboratory Experiment

All plant seeds were germinated and grown to the appropriate stages as described in the UV‐B treatment of plants. One hour prior to UV‐B treatment, a solution of 1 mg L^−1^ flavonoids (pelargonidin‐3‐*O*‐glucoside, kaempferol‐3‐*O*‐glucoside, quercetin‐3‐*O*‐glucoside) was thoroughly sprayed on the seedlings, followed by reapplication every 6 h. Deionized water served as the control.

### Treatment of Plants with Flavonoids—Field Experiment

Conducted in Liangshan Prefecture (3500 m), Sichuan Province, China, three varieties of Tartary buckwheat (ZK3, CQ1, CQ2) were sown on April 15. The first treatment began 15 days post‐sowing (after the emergence of two true leaves), with a 2 mg L^−1^ solution of pelargonidin‐3‐*O*‐glucoside sprayed on the seedlings, followed by reapplication every 15 days, with deionized water as the mock treatment. Upon maturity, the number of seeds per plant and plant height were measured. Each group consisted of 100 plants, with each treatment replicated three times.

### Measurement of Flavonoid Content

The determination of flavonoids in plant materials was conducted following protocol as described previously.^[^ ^37^ ^]^ The plant material was thoroughly washed with pure water, dried overnight at 65 °C, then ground and filtered through an 80‐mesh sieve. For the extraction of flavonoids, 0.1 g of the powder was used with 10 mL of 80% methanol (v/v). Ultrasonic extraction was performed for 30 min at 50 °C and 80 kHz. The crude extracts were filtered through a 0.22 µm organic microporous filter and then analyzed using UPLC‐QQQ/MS (Agilent UPLC 1290II‐G6400 QQQ MS, Agilent, Santa Clara, CA, USA). The mobile phases consisted of solvent A: pure water containing 0.1% formic acid, and solvent B: acetonitrile containing 0.1% formic acid. The sample determination was conducted using a gradient program: initial conditions were 98% A and 2% B, held for 2 min. From 2 to 4 min, a programmed linear gradient to 90% A and 10% B was applied, and then from 4 to 11 min, a linear gradient to 20% A and 80% B was applied. Subsequently, the composition was adjusted to 2% A and 98% B within 0.10 min and held for 1.9 min. Finally, the composition was adjusted back to 98% A and 2% B within 0.10 min and held for 1.9 min. The column oven temperature was set to 40 °C, with an injection volume of 2 µL. The eluate was alternately connected to an ESI‐triple quadrupole LIT (Q TRAP)‐MS. The flavonoid content was determined by comparing the peak areas to authentic standards (Sigma–Aldrich, USA).

### Yeast One‐Hybrid Assay

The pHis vector was used as a reporter, integrating the 5×BOX2 construct. As an effector, FtMADS1 was introduced into the pGADT7 vector, which contains a GAL4 transcriptional activation domain. The effector and reporter were, therefore introduced into the Y1H gold strain, with each strain containing the reporter gene. Transformants were cultured on a minimal synthetic defined (SD)‐glucose medium lacking both Leu (‐L) and Trp (‐T). The Y1H assay was performed following the instructions from the manufacturer (Matchmaker Y1H System; Clontech, USA). The use of all primers in the assay is documented in Data Set 2 (Supporting Information).

### Yeast Two‐Hybrid Assay

The pGADT7 vector facilitated individual integration of the coding sequence regions (CDS) of FtPAK, FtMADS1, and through homologous recombination. Similarly, the pGBKT7 vector was used for inserting the full‐length sequences of *FtMADS2*. Yeast transformation followed the provided guidelines from the manufacturer (Clontech, USA). The yeast strain Y2H was simultaneously introduced to the bait and prey vectors and cultivated together on a selective SD medium lacking Leu and Trp. After incubating at 30 °C for 3–4 days, yeast cells were diluted 10‐ 100‐ and 1000‐fold and applied onto selection plates supplemented with an SD medium lacking Leu, Trp, His, and Ade. The plates were incubated at 30 °C until colonies of yeast cells were visible. The use of all primers in the assay is documented in Data Set 2 (Supporting Information).

### GST Pull‐Down

The full‐length CDS sequences of FtPAK and FtMADS2 or FtMADS1 are separately recombined into the expression vectors pET‐28a and pGXT‐4T‐1. These recombined plasmids are transformed into *Escherichia coli* strain Arctic‐Express (DE3) and induced for expression. Then, the fusion proteins are purified using GST or Ni‐NTA agarose. The purified FtPAK‐His, FtMADS‐GST, and ATP are mixed and incubated. The fusion protein mixture is added to a column previously loaded with GST‐affinity resin, allowing for affinity adsorption. Subsequently, a washing step is performed to remove nonspecifically bound proteins. Finally, the GST‐labeled fusion proteins and their complexes are eluted. The eluted samples are subjected to SDS‐PAGE electrophoresis to evaluate purity and molecular size. FtPAK‐His and FtMADS proteins in the wash buffer and protein elution buffer are detected using Western blotting. The Anti His‐Tag Mouse Monoclonal Antibody (CW0286, Cwbio, China) and Anti GST‐Tag Mouse Monoclonal Antibody (CW0884, Cwbio, China) were employed for this purpose.

### Western Blot Analysis

All proteins purified through prokaryotic expression were detected using Western blot analysis (Figure S3, Supporting Information). The Western blot method was performed following the previous literature^50^ with minor modifications. In a nutshell, the samples were first subjected to 10% SDS/PAGE and subsequently, transferred to a poly‐vinylidene difluoride (PVDF) membrane. The membrane is then blocked with 5% semi‐skimmed milk powder at room temperature and probed with the respective primary antibodies. Antibody of His (CW0286, Cwbio, China), MBP (CW0299, Cwbio, China) and GST (CW0884, Cwbio, China) and anti‐mouse IgG (1:8000; CW0102, Cwbio, China) antibodies were used for immunoblotting.

### EMSA

Oligonucleotide probes (Data Set 2, Supporting Information) were synthesized and biotinylated at the 5′ end by Beijing Tsingke Biotech (Beijing, China). EMSA was conducted using the LightShift Chemiluminescent EMSA Kit (Thermo scientific, USA) in accordance with the instructions provided by the manufacturer.

### Dual Bioluminescence Assay

Target DNA sequences were inserted into the reporter vector pGreenII0800‐LUC, which also carries a *35Spro:REN* reporter as control. The CDS of the target protein was recombinantly inserted into pGreen‐62SK as effectors. Primers are listed in (Data Set 2, Supporting Information). The recombinant vector was transformed into Agrobacterium GV3101 and cultured at 28 °C. The *N. benthamiana* leaves were then injected with Agrobacterium culture, and incubated in the dark for 24 h, followed by a light cycle (23 °C/22 °C, 16 h day/8 h night) for 48 h. Under the condition of shading, the reaction mixture (3 mg mL^−1^ D‐fluorescent potassium salt and 10 µL of 10% TritonX‐100) was evenly spread on the infiltrated site of tobacco leaves, and placed in the dark until the reaction solution (≈15–20 min) could not be seen. For LUC reporter assays, luminescence signals from pavement cells were detected after applying 3 mg mL^−1^ luciferin by a charge‐coupled device (CCD) system (plant in vivo imaging system). For the dual‐bioluminescence assay, the luminescence from LUC and REN was detected using the Dual‐Luciferase Reporter assay kit^51^.

### Real‐Time Quantitative PCR (qRT‐PCR)

The total RNA was isolated from the plant material using a plant RNA extraction kit (DP452, Tiangen, China). The extracted RNA was reversely transcribed into the cDNA by HiScript III RT SuperMix 519 (+gDNA wiper; R323, v21.1, Vazyme, Nanjing, China). The quantitative reverse transcription polymerase chain reaction (qRT‐PCR) was performed using the Taq Pro Universal SYBR qPCR Master Mix (Q712, v20.1, Vazyme, China) in accordance with the manufacturer's instructions. Primers are depicted in Data Set 2 (Supporting Information).

### Luciferase Complementation Imaging Assay (LCA)

For LCA assays, the full length of the target protein was amplified using specific primers (Data Set 2, Supporting Information) and introduced into pCAMBIA1300‐cLUC or pCAMBIA1300‐nLUC. The recombinant vectors were transformed into GV3101 and then co‐transformed into the *N. benthamiana leaves*. Luminescence signals from pavement cells were detected after applying 3 mg mL^−1^ luciferin by a charge‐coupled device (CCD) system (plant in vivo imaging system). The luminescence from LUC was detected using a Mithras LB940 microplate reader^52^..

### Statistical Analysis

GraphPad Prism 8.0 and SPSS22 were employed for conducting the statistical analysis. The statistical significance of the observed differences was assessed using Student's t‐test (*P < 0.05; **P < 0.01). Run molecular docking calculations using AMDock, and visualize and analyze the results using PyMOL.

## Conflict of Interest

The authors declare no conflict of interest.

## Author Contributions

M.Z. designed and managed the project. M.Z., Y.H., and K.Z. organized the funding for this research. D.L. and W.L. provided the genetic materials. X.H., Y.H., K.Z., Y.S., H.L., and X.W. performed data analysis and figure design. X.H., H.Z., D.L., Z.Y., Y.X., and Y.O. performed most of the experiments. X.H., Y.H., K.Z., S.H.W., M.Q., M.I.G., and A.R.F. wrote the manuscript. All authors read and approved the manuscript.

## Supporting information

Supporting Information

Supplementary TableS1

## Data Availability

The data that support the findings of this study are available in the supplementary material of this article.
