# Correction to “Evolution and Domestication of a Novel Biosynthetic Gene Cluster Contributing to the Flavonoid Metabolism and High‐Altitude Adaptability of Plants in the *Fagopyrum* Genus”

**DOI:** 10.1002/advs.75882

**Published:** 2026-05-28

Xu Huang, Yuqi He, Kaixuan Zhang, Yaliang Shi, Hui Zhao, Dili Lai, Hao Lin, Xiangru Wang, Zhimin Yang, Yawen Xiao, Wei Li, Yinan Ouyang, Sun Hee Woo, Muriel Quinet, Milen I. Georgiev, Alisdair R. Fernie, Xu Liu, and Meiliang Zhou, “Evolution and Domestication of a Novel Biosynthetic Gene Cluster Contributing to the Flavonoid Metabolism and High‐Altitude Adaptability of Plants in the *Fagopyrum* genus,” *Advanced Science* 11, no. 43 (2024): e2403603, https://doi.org/10.1002/advs.202403603.

In the above article, the authors would like to correct Supplemental Figures 38, 39, and 41 with updated figures. In Supplemental Figure 39, a partial image was mistakenly taken from Supplemental Figure 38; the correct Supplemental Figures 38 and 39 are shown below. In Supplemental Figure 41, the band in the second lane was slightly shifted during figure preparation; the correct Supplemental Figure 41 is shown below.

These errors do not affect the results.


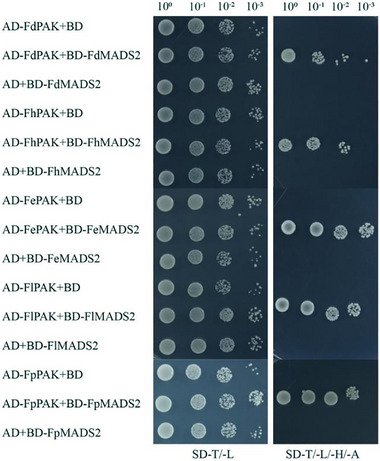


**Supplemental Figure 38** The yeast two‐hybrid (Y2H) results of the interaction between PAK and MADS2 in the *Fagopyrum* genus. SD‐L/‐T, SD basic medium lacking Leu and Trp; SD‐L/‐T/‐H/‐A, SD basal medium lacking Leu, Trp, His, and Ade.


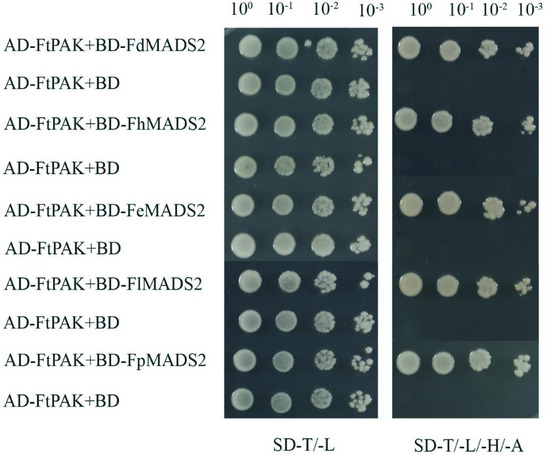


**Supplemental Figure 39** Y2H assays were used to detect the interaction between FtPAK and the MADS2 of other species in the *Fagopyrum* genus.


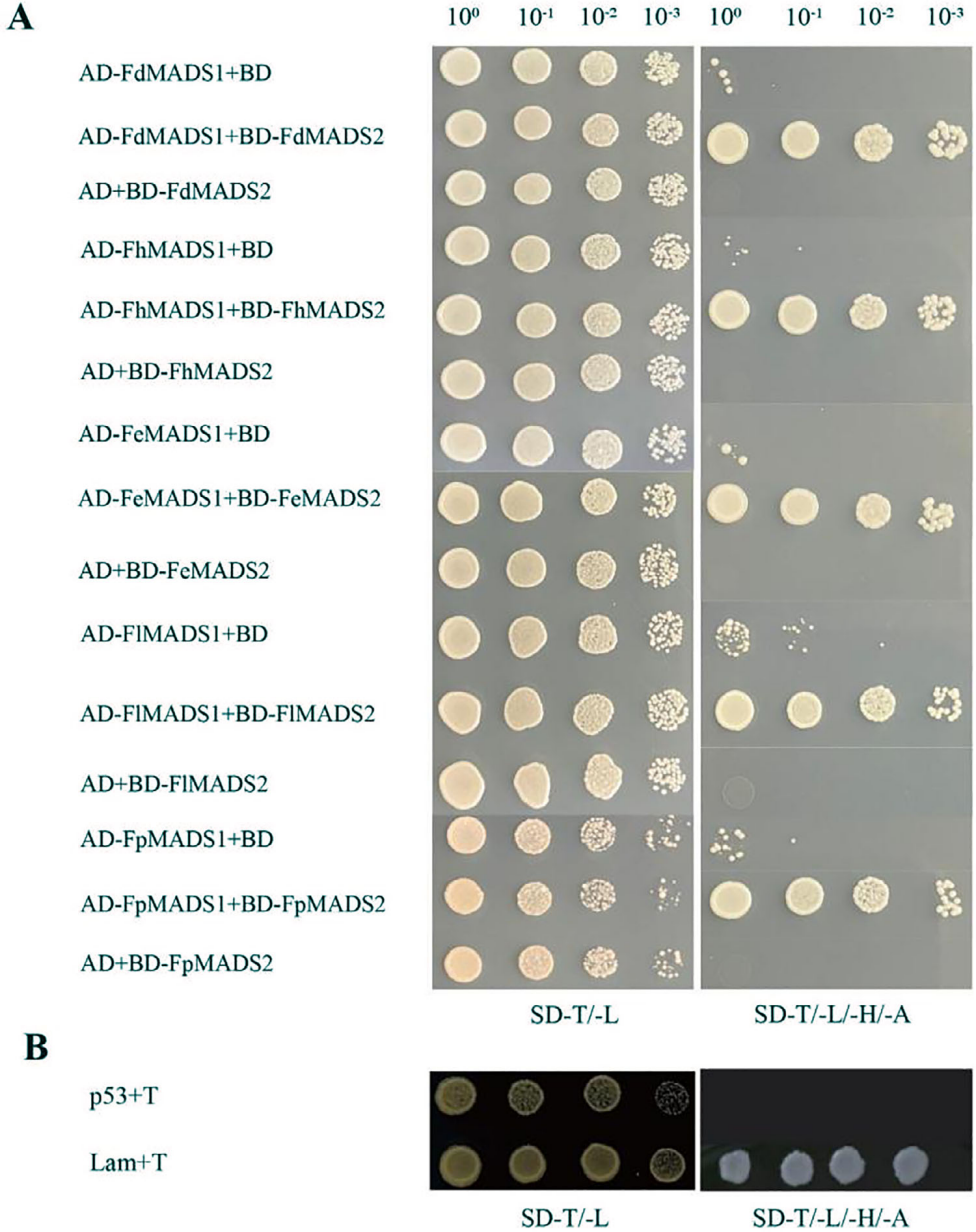


**Supplemental Figure 41** (A) The yeast two‐hybrid (Y2H) results of the interaction between MADS2 and MADS1 in the *Fagopyrum* genus. SD‐L/‐T, SD basic medium lacking Leu and Trp; SD‐L/‐T/‐H/‐A, SD basal medium lacking Leu, Trp, His, and Ade. **B**) P53+T denotes the positive control for the Y2H, while Lam+T denotes the negative control.

