# Supplementary material for: Evolution and Domestication of a Novel Biosynthetic Gene Cluster Contributing to the Flavonoid Metabolism and High‐Altitude Adaptability of Plants in the Fagopyrum Genus
Source: Adv Sci (Weinh). 2024 Sep 23;11(43):2403603. doi: 10.1002/advs.202403603 (PMC11578379; doi:10.1002/advs.202403603)
Supplement: Supplementary file 1 — Supporting Information [file ADVS-11-2403603-s002.pdf]

ADVANCED SCIENCE
Open Access

Supporting Information

for *Adv. Sci.*, DOI 10.1002/advs.202403603

Evolution and Domestication of a Novel Biosynthetic Gene Cluster Contributing to the Flavonoid Metabolism and High-Altitude Adaptability of Plants in the *Fagopyrum* Genus

*Xu Huang, Yuqi He, Kaixuan Zhang, Yaliang Shi, Hui Zhao, Dili Lai, Hao Lin, Xiangru Wang, Zhimin Yang, Yawen Xiao, Wei Li, Yinan Ouyang, Sun Hee Woo, Muriel Quinet, Milen I. Georgiev, Alisdair R. Fernie, Xu Liu and Meiliang Zhou**

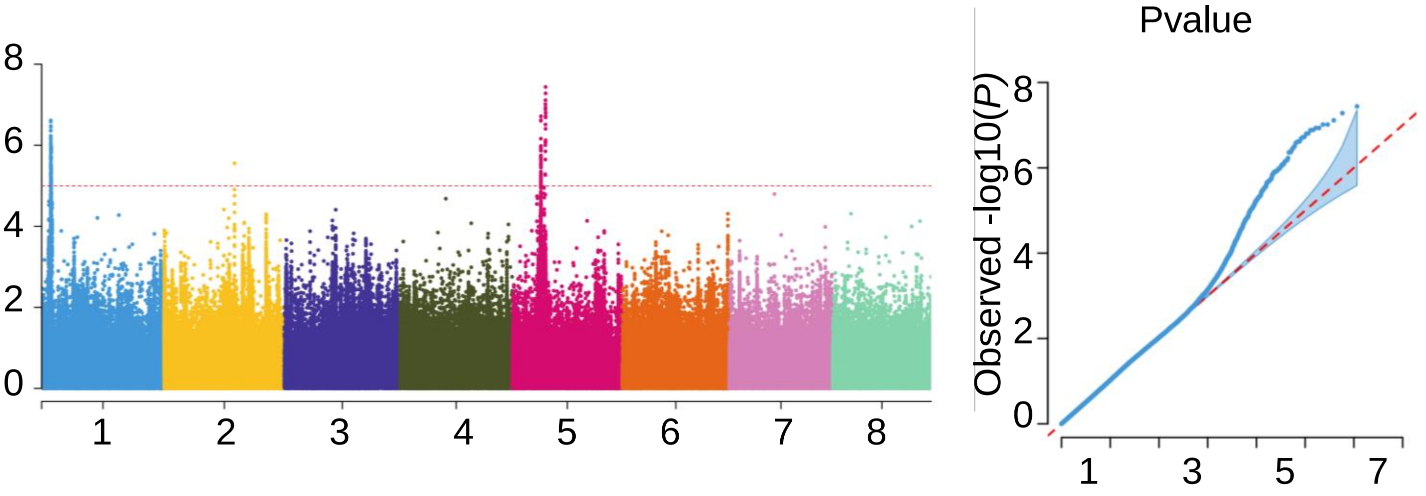

Supplemental Figure 1. A) Manhattan plot displays the mGWAS result of kaempferol-3-*O*-rutinoside content. The dashed line indicates the threshold $-\log_{10}P = 5$. B) QQ plot displays the mGWAS result of kaempferol-3-*O*-rutinoside content. The dashed line indicates the threshold $-\log_{10}P = 5$.

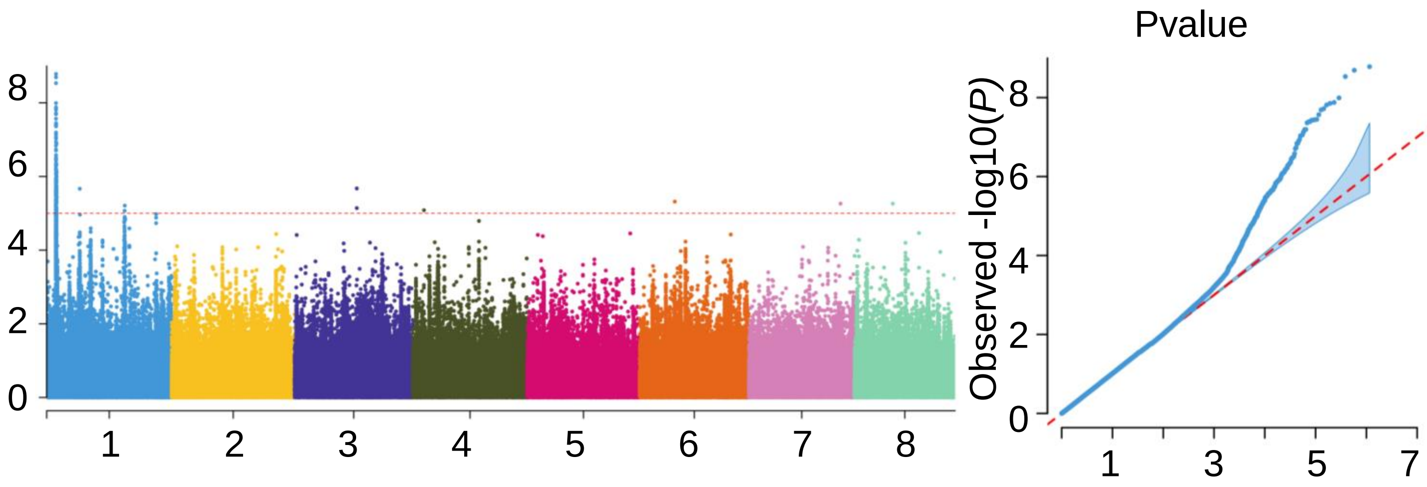

Supplemental Figure 2. A) Manhattan plot displays the mGWAS result of kaempferol-3-*O*-glucoside-7-*O*-rhamnoside content. The dashed line indicates the threshold -$\log_{10}P$ = 5. B) QQ plot displays the mGWAS result of kaempferol-3-*O*-glucoside-7-*O*-rhamnoside content. The dashed line indicates the threshold -$\log_{10}P$ = 5.

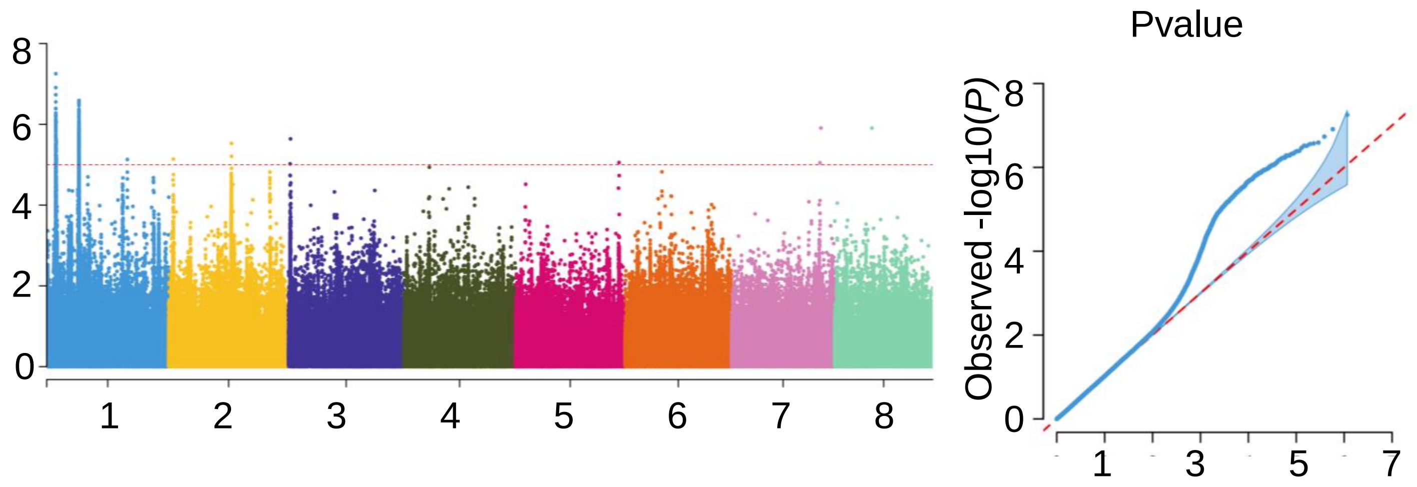

Supplemental Figure 3. A) Manhattan plot displays the GWAS result kaempferol-3-*O*-neohesperidoside content. The dashed line indicates the threshold -$\log_{10}P$ = 5. B) QQ plot displays the GWAS result of kaempferol-3-*O*-neohesperidoside content. The dashed line indicates the threshold -$\log_{10}P$ = 5.

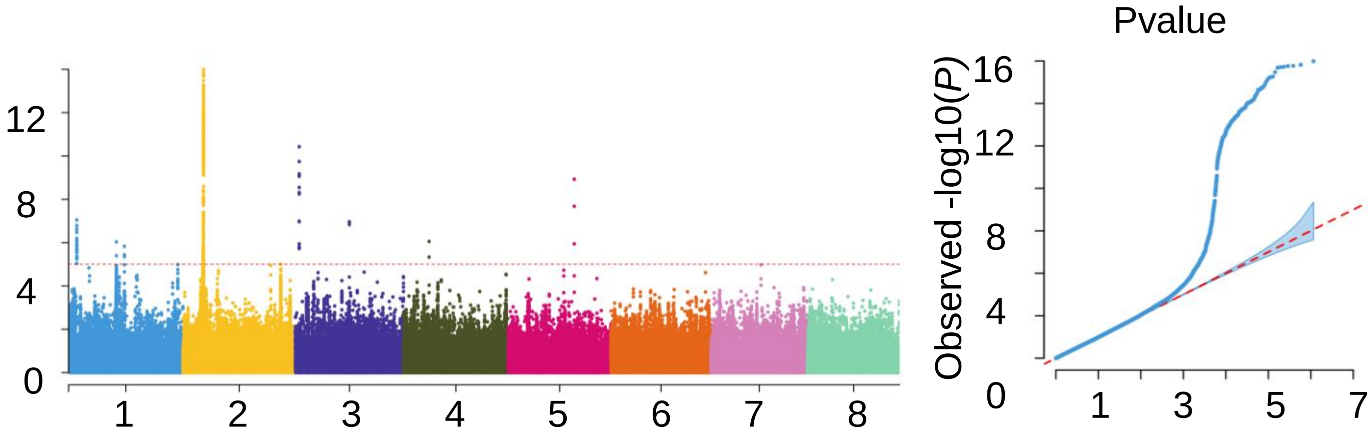

Supplemental Figure 4. A) Manhattan plot displays the GWAS result quercetin-3-*O*-glucoside content. The dashed line indicates the threshold -log$_{10}$*P* = 5. B) QQ plot displays the GWAS result of quercetin-3-*O*-glucoside content. The dashed line indicates the threshold -log$_{10}$*P* = 5.

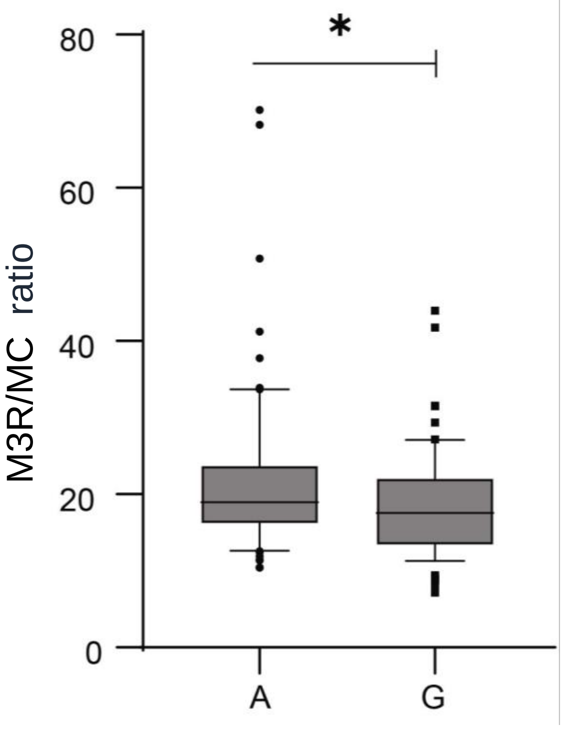

Supplemental Figure 5. Box plots show the ratio between glycosylated myricetin and myricetin-3-*O*-rutinoside within the buckwheat germplasm population, across genotype A and genotype G. M3R represents myricetin-3-*O*-rutinoside, and MC represents myricetin.*$P < 0.01$, Student's *t* test. The content was log2 transformed.

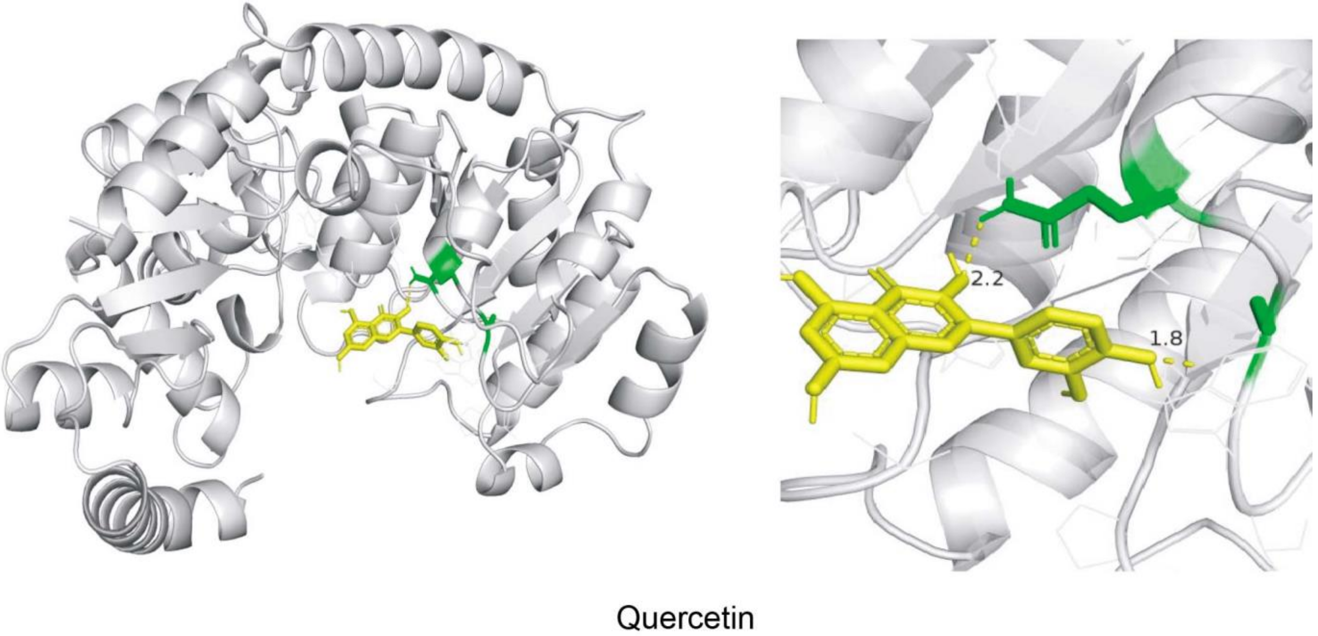

Supplemental Figure 6. Three-dimensional simulation diagram of molecular docking of FtUFGT3 with quercetin.

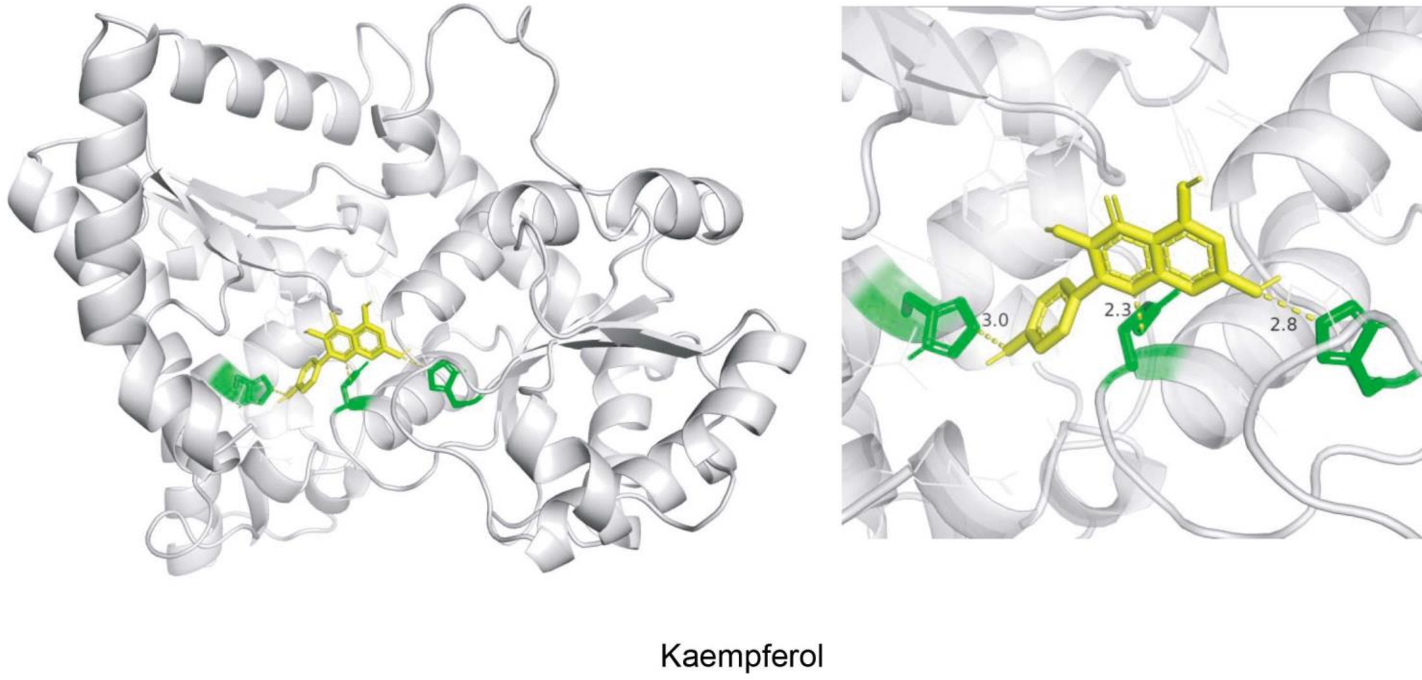

Supplemental Figure 7. Three-dimensional simulation diagram of molecular docking of FtUFGT3 with kaempferol.

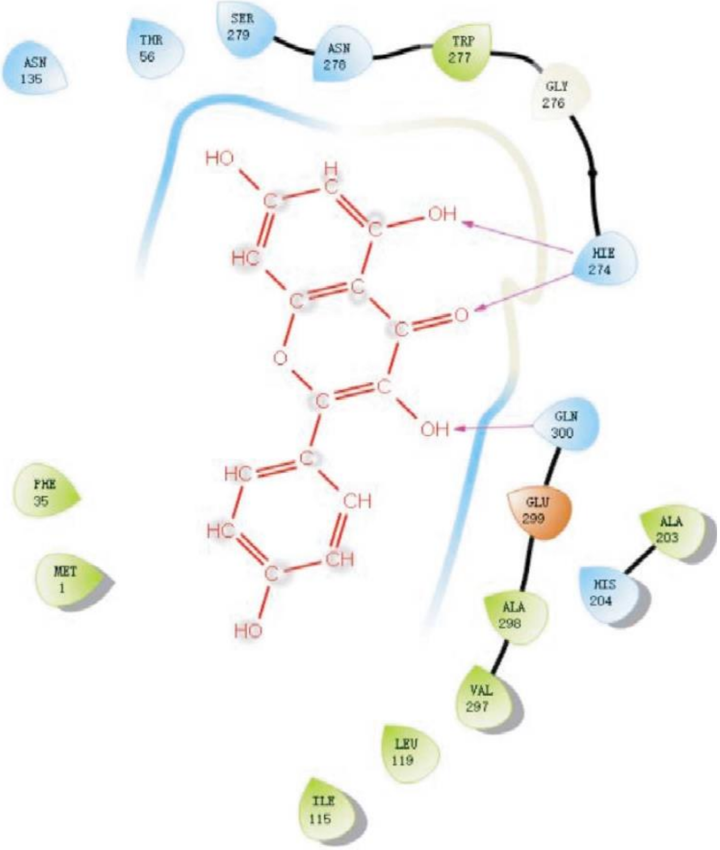

Kaempferol

Supplemental Figure 8. Two-dimensional simulation diagram of molecular docking of FtUFGT3 with kaempferol.

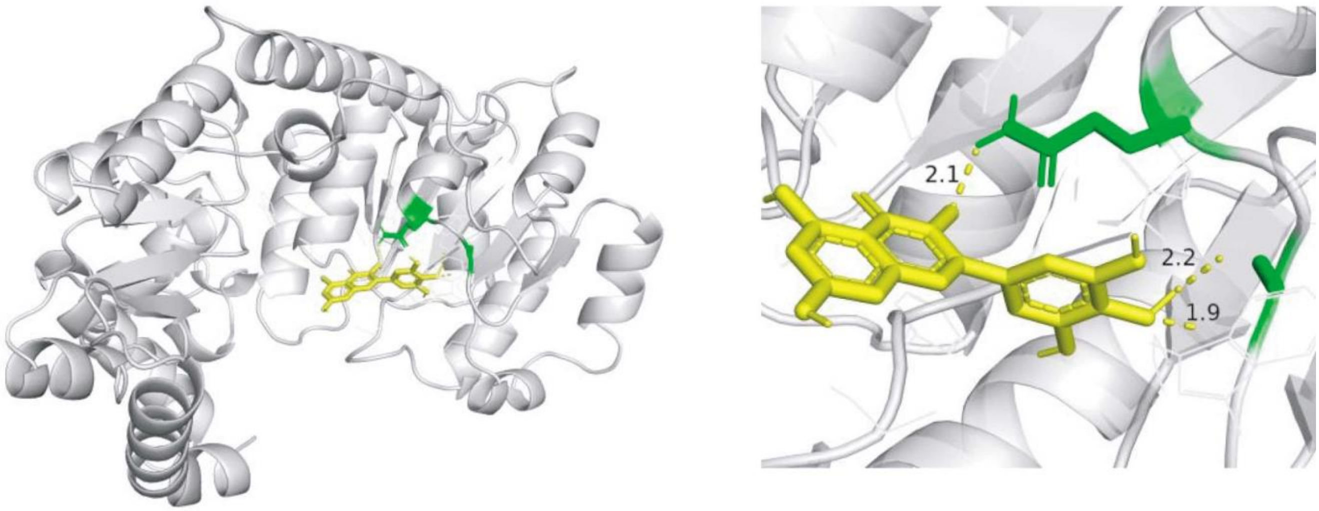

Myricetin

Supplemental Figure 9. Three-dimensional simulation diagram of molecular docking of FtUFGT3 with myricetin.

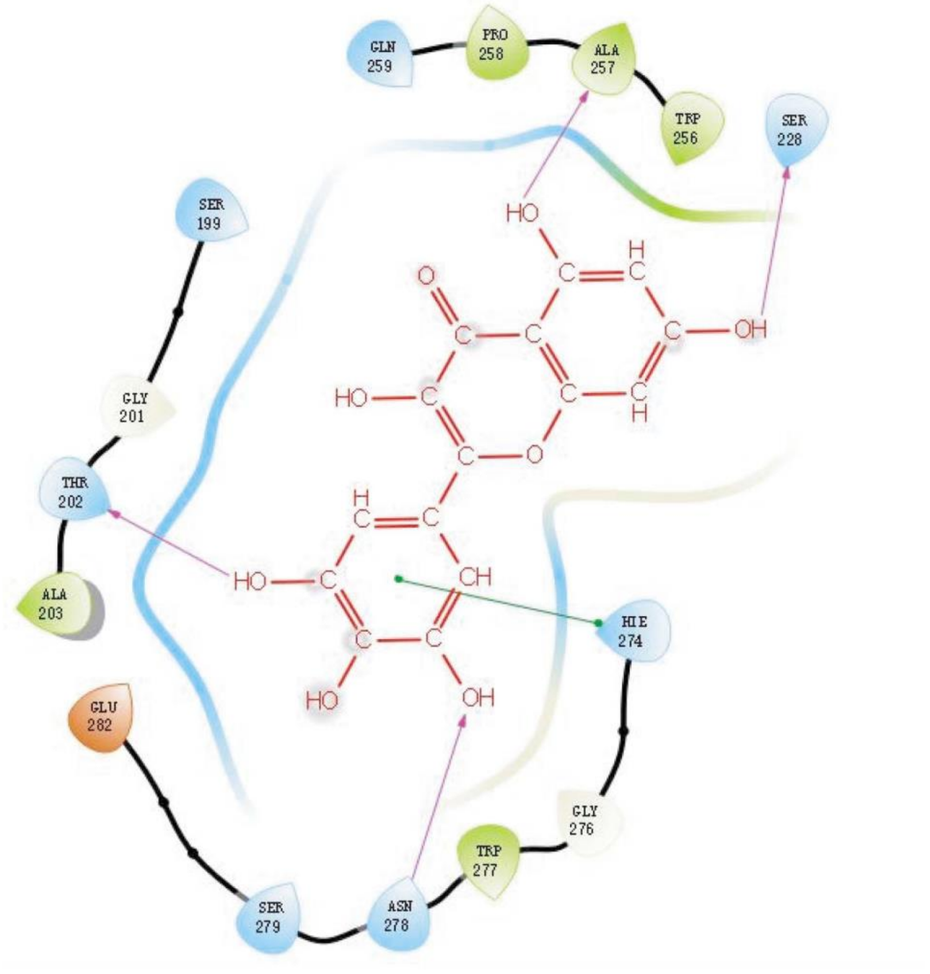

Myricetin

Supplemental Figure 10. Two-dimensional simulation diagram of molecular docking of FtUFGT3 with myricetin.

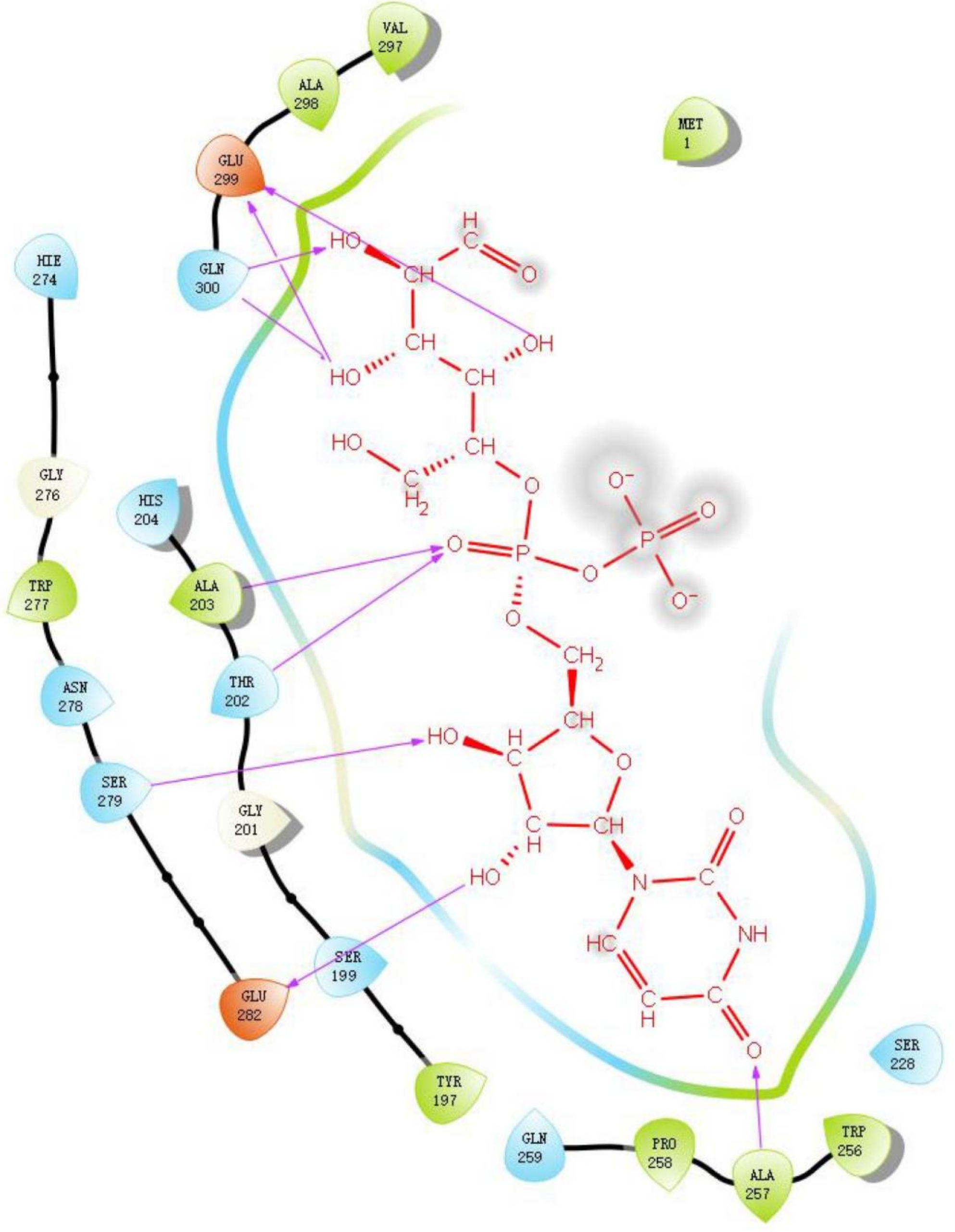

Supplemental Figure 11. Two-dimensional simulation diagram of molecular docking of FtUFGT3 with UDP-glucoside.

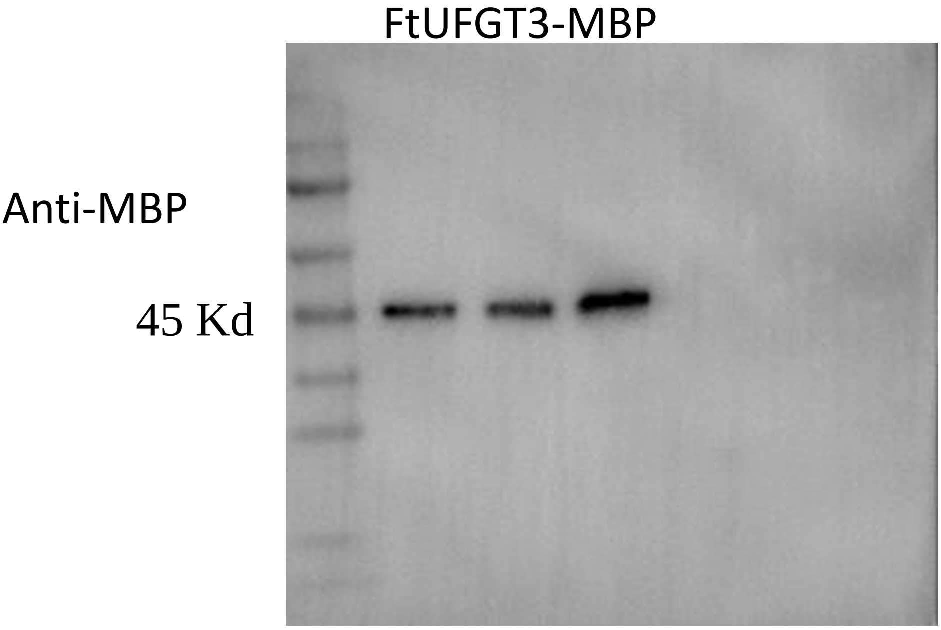

Supplemental Figure 12. The results of purified FtUFGT3-MBP recombinant protein were detected by Western blot.

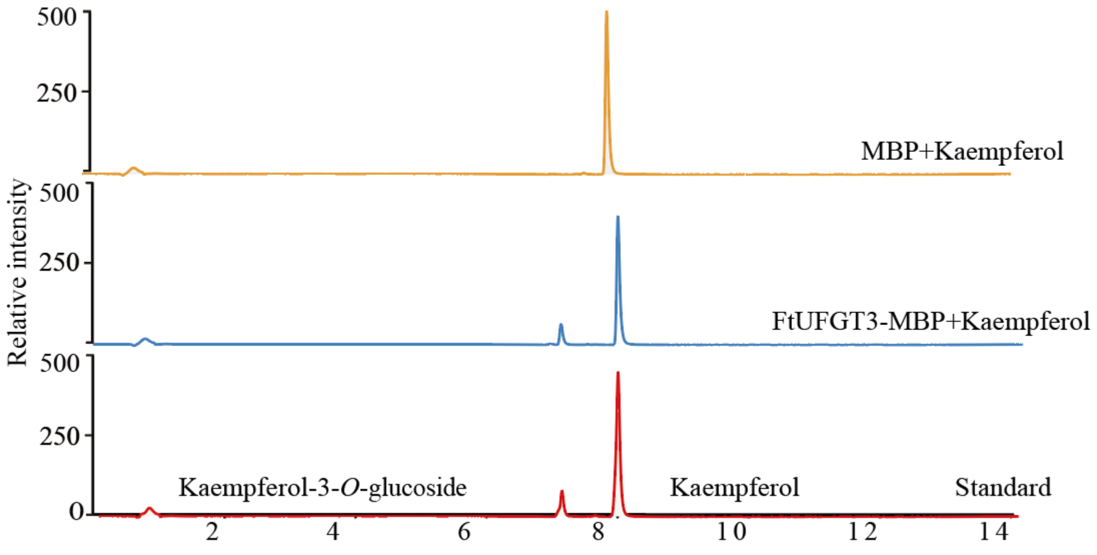

Supplemental Figure 13. Enzymatic assay for kaempferol of FtUFGT3 *in vitro*.

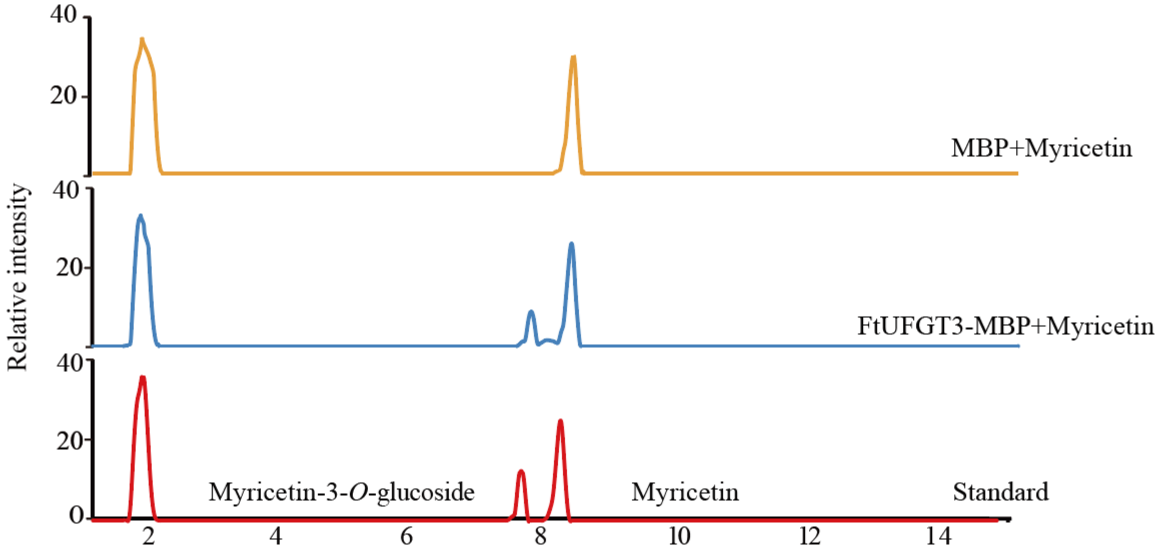

Supplemental Figure 14. Enzymatic assay for myricetin of FtUFGT3 *in vitro*.

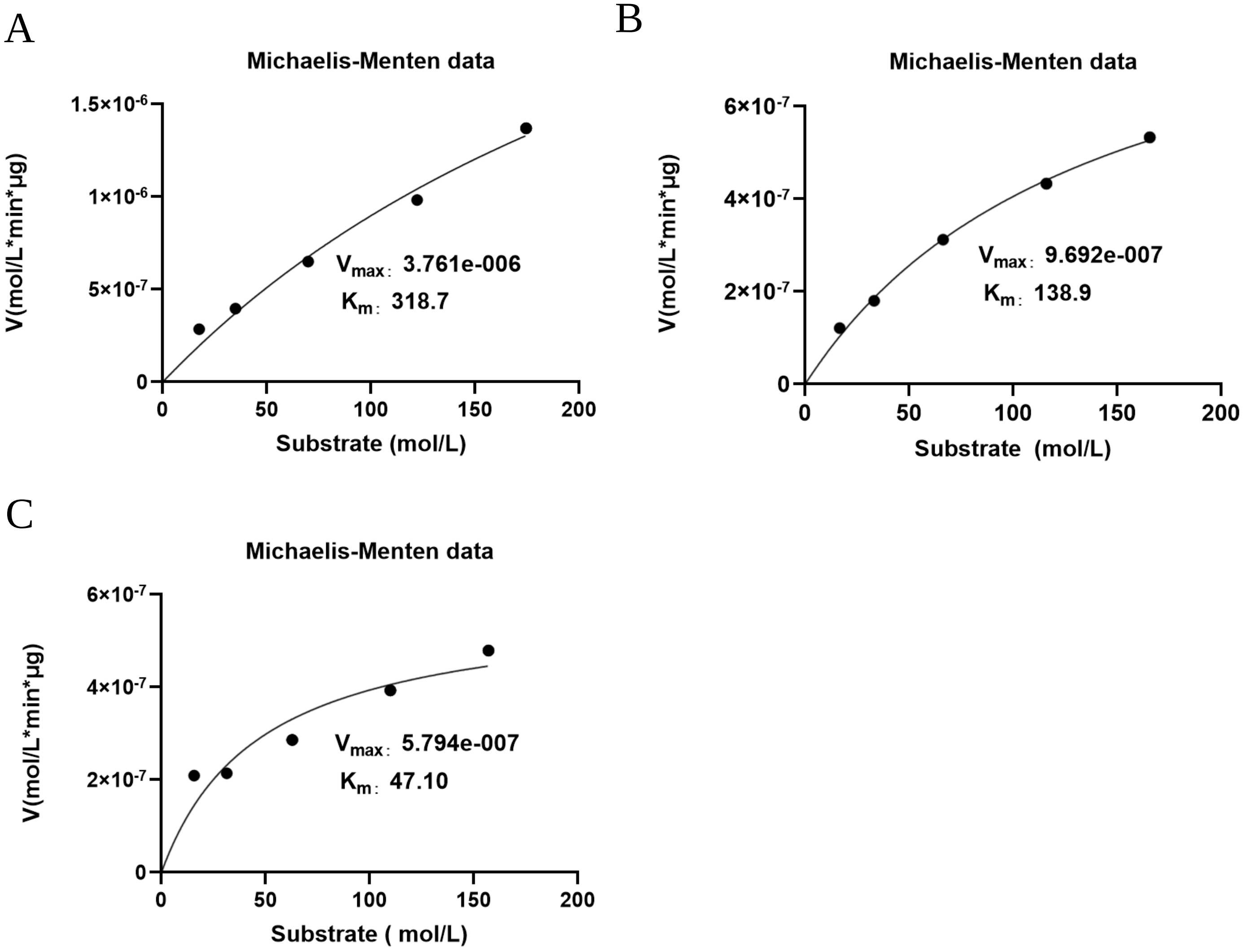

Supplemental Figure 15. Kinetic curves of FtUFGT3 catalyzing different substrates.
A) Kinetic curve of FtUFGT3 catalyzing kaempferol. Enzyme activity was measured under standard assay conditions with substrate concentrations ranging from 17.45 μM to 174.5 μM. The curve was fitted using non-linear regression to the Michaelis-Menten equation to determine the kinetic parameters K_m and V_{max}. The K_m value was 318.7 μM, and the V_{max} was 3.761e-006 μM/min.
B) Kinetic curve of FtUFGT3 catalyzing quercetin. Enzyme activity was measured under standard assay conditions with substrate concentrations ranging from 16.56 μM to 165.6 μM. The curve was fitted using non-linear regression to the Michaelis-Menten equation to determine the kinetic parameters K_m and V_{max}. The K_m value was 138.9 μM, and the V_{max} was 9.692e-007 μM/min.
C) Kinetic curve of FtUFGT3 catalyzing myricetin. Enzyme activity was measured under standard assay conditions with substrate concentrations ranging from 15.7 μM to 157 μM. The curve was fitted using non-linear regression to the Michaelis-Menten equation to determine the kinetic parameters K_m and V_{max}. The K_m value was 47.10 μM, and the V_{max} was 5.794e-007 μM/min.

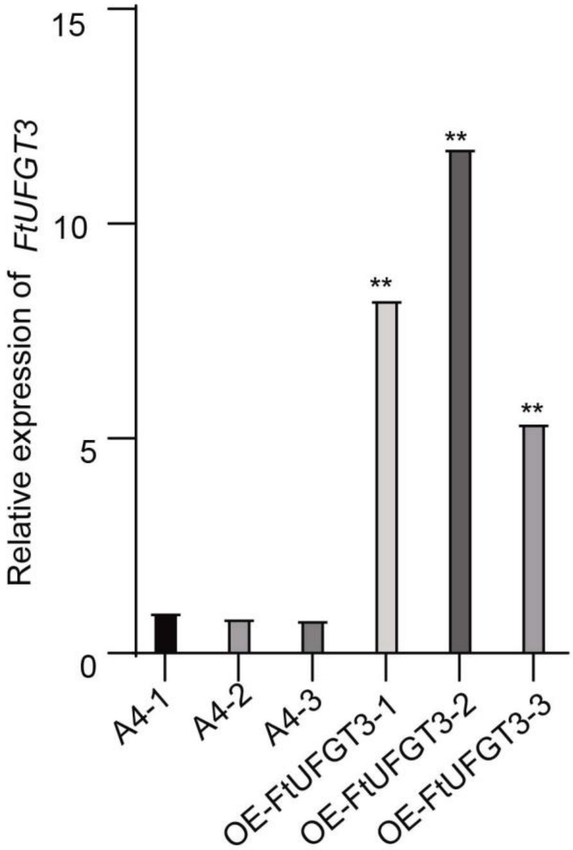

Supplemental Figure 16. The relative expression level of *FtUFGT3* in three *OE-FtUFGT3* hairy root lines. Data show the arithmetic mean ± SD from 3 biological replicates. ***P* < 0.01, Student's t test..

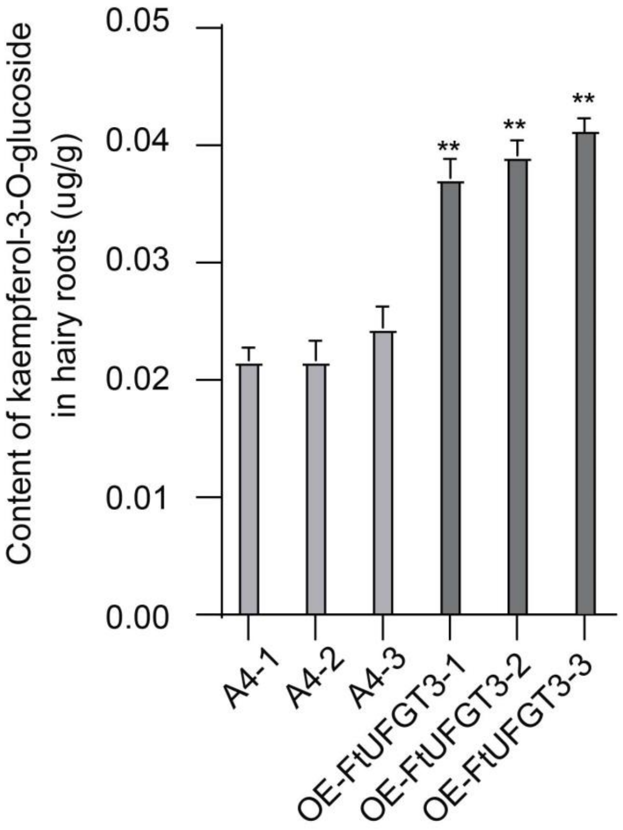

Supplemental Figure 17. Content of kaempferol-3-*O*-glucosidein three *OE-FtUFGT3* hairy root lines. Data show the arithmetic mean ± SD from 3 biological replicates. $**P < 0.01$, Student's t test.

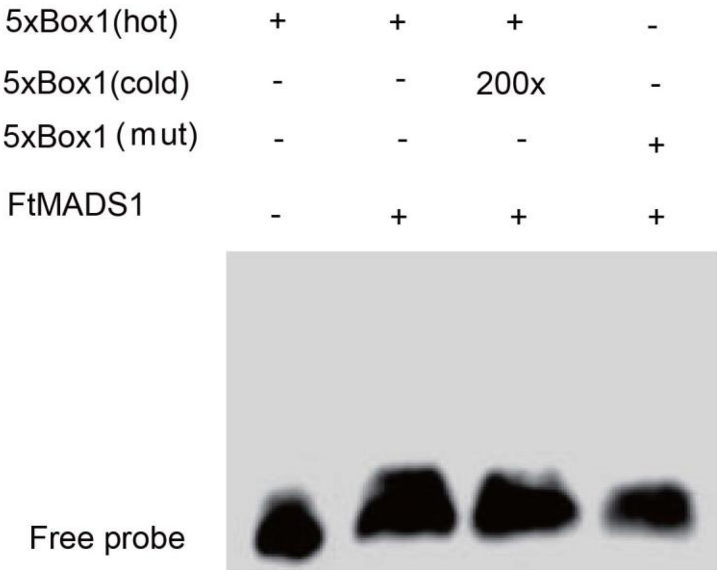

Supplemental Figure 18. Results of the EMSA for the interaction between FtMADS1 and 5×BOX1.

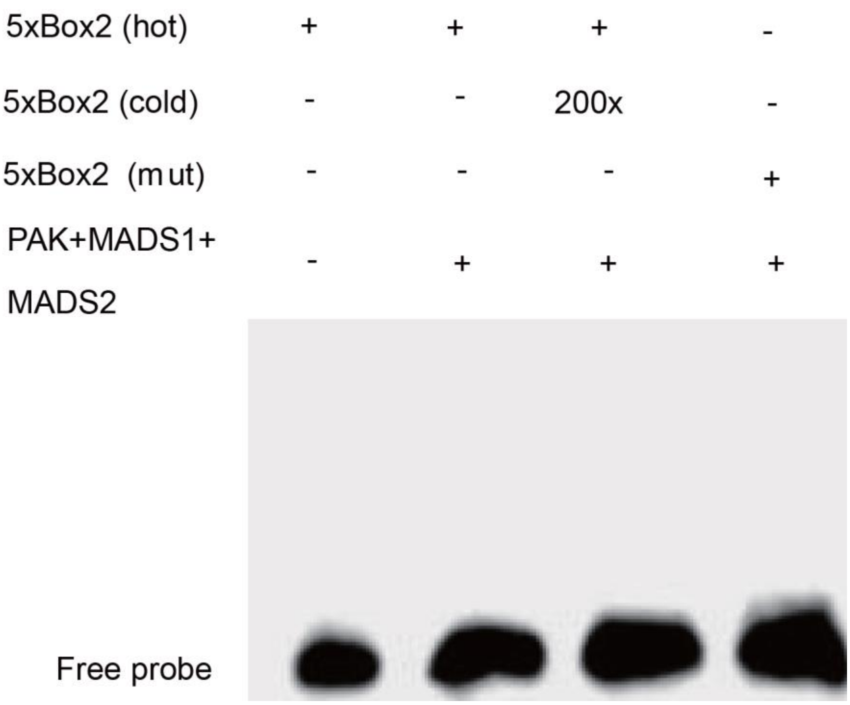

Supplemental Figure 19. Results of the EMSA for the interaction between FtPAK+FtMADS1+FtMADS2 and 5×BOX1.

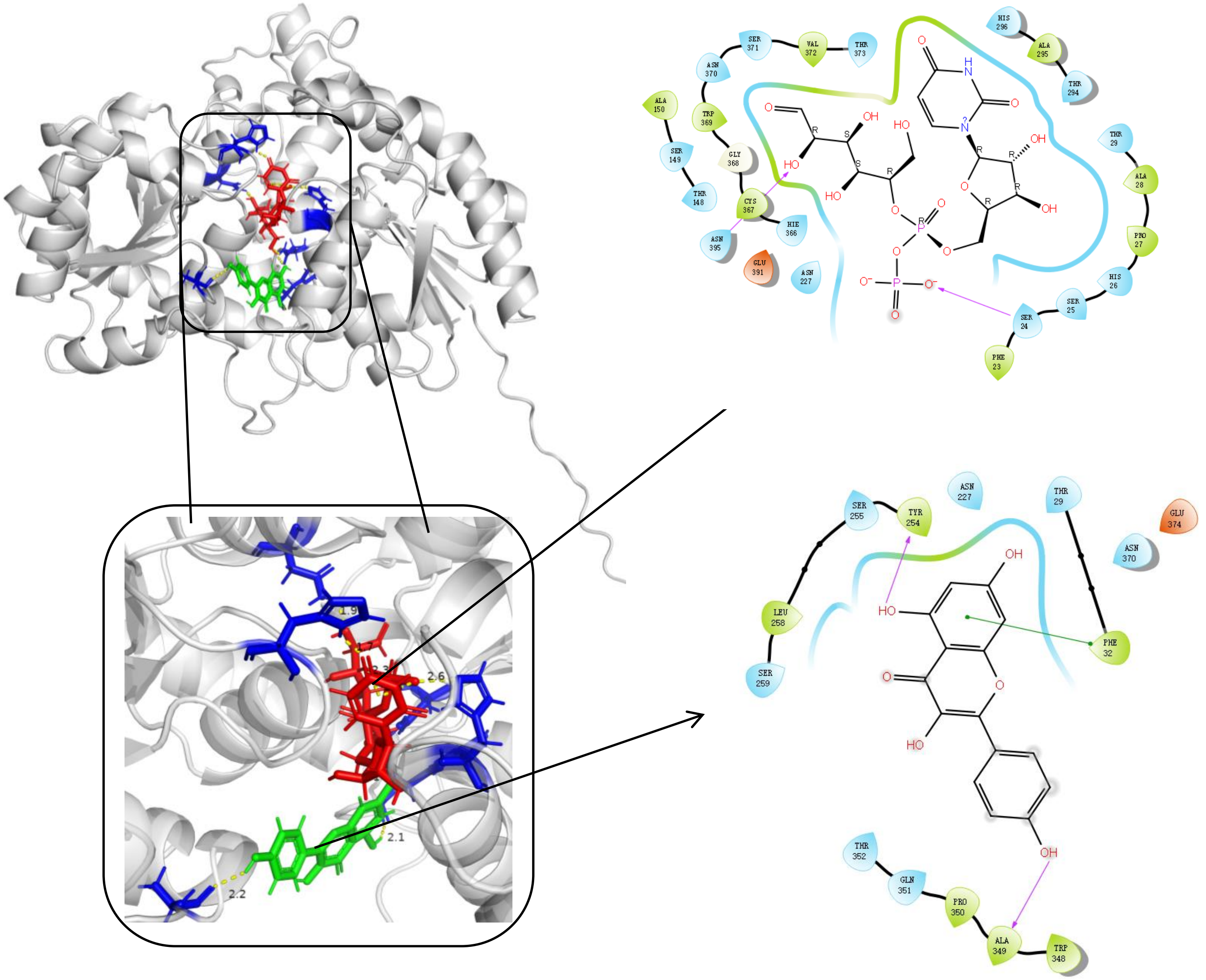

Supplemental Figure 20. Molecular docking simulation of FdUFGT3 with UDP-glucose and kaempferol.

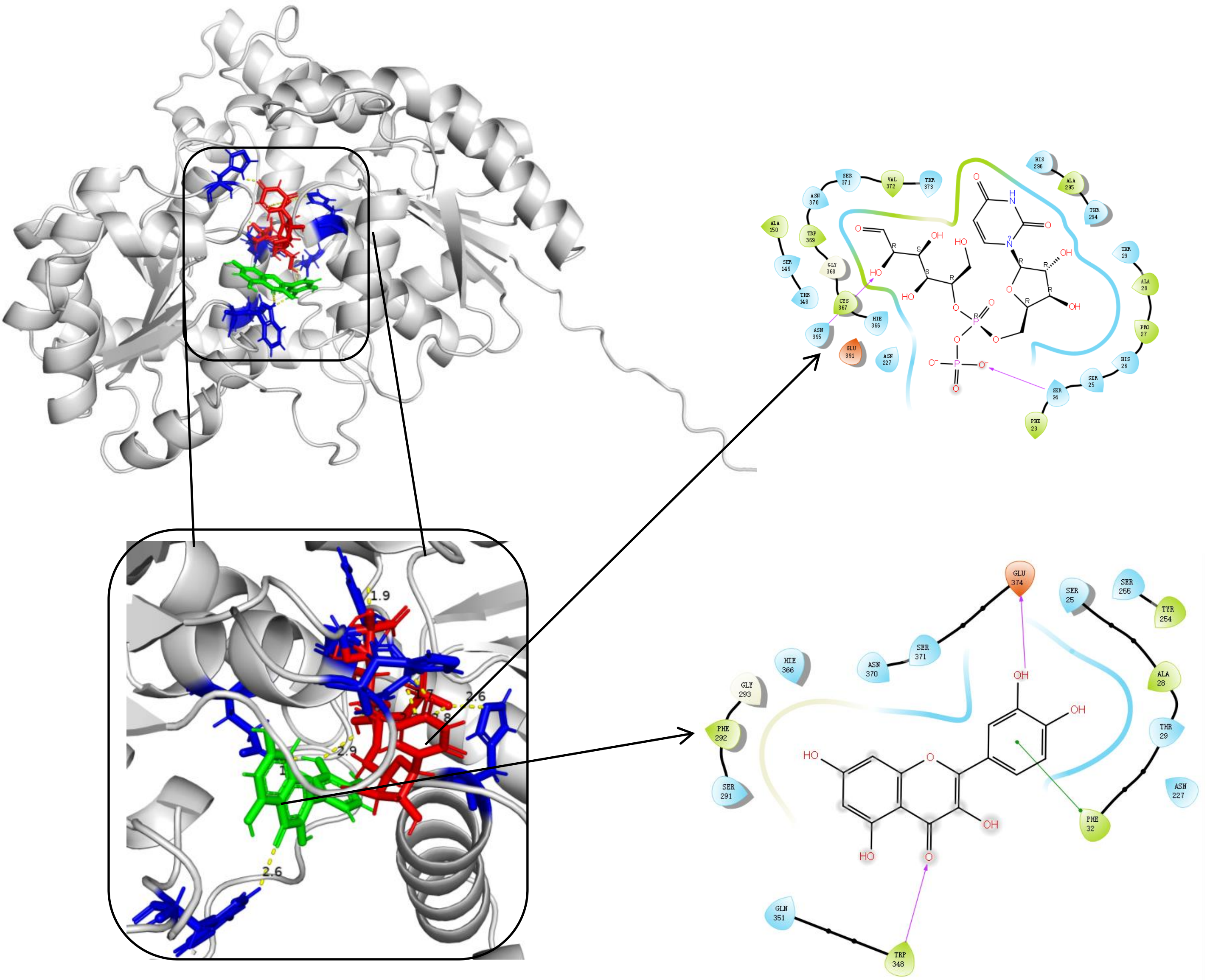

Supplemental Figure 21. Molecular docking simulation of FdUFGT3 with UDP-glucose and quercetin

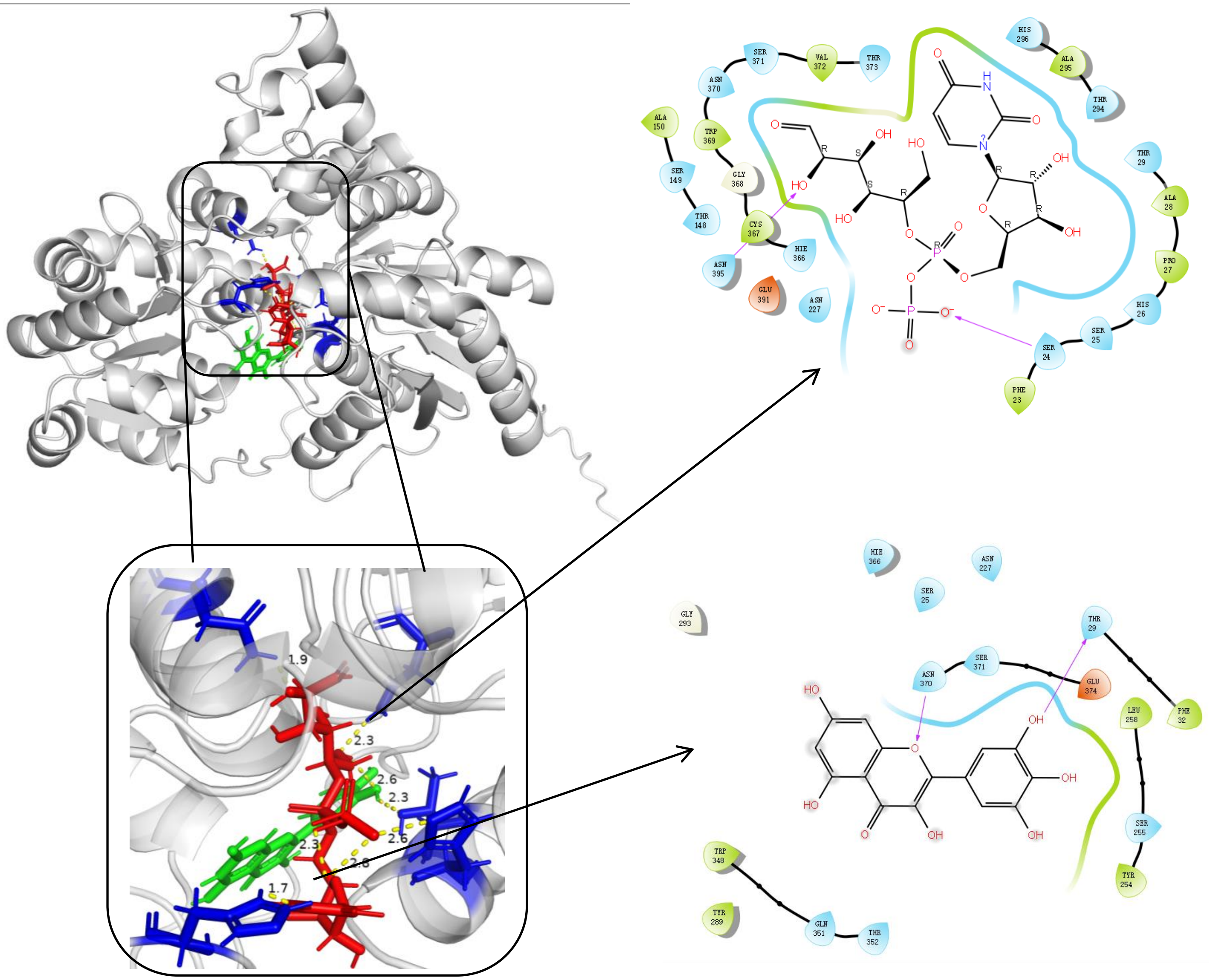

Supplemental Figure 22. Molecular docking simulation of FdUFGT3 with UDP-glucose and myricetin.

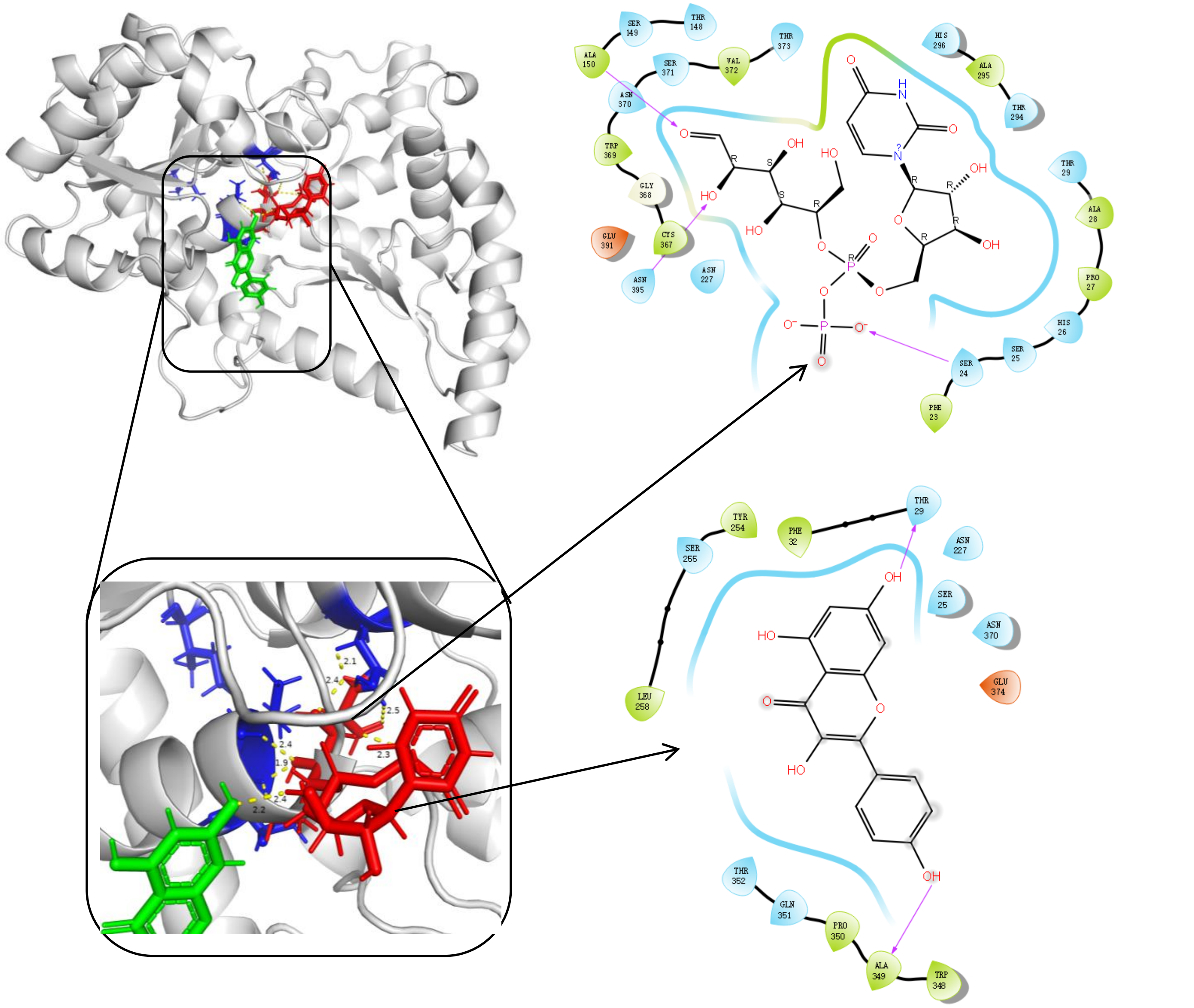

Supplemental Figure 23. Molecular docking simulation of FhUFGT3 with UDP-glucose and kaempferol.

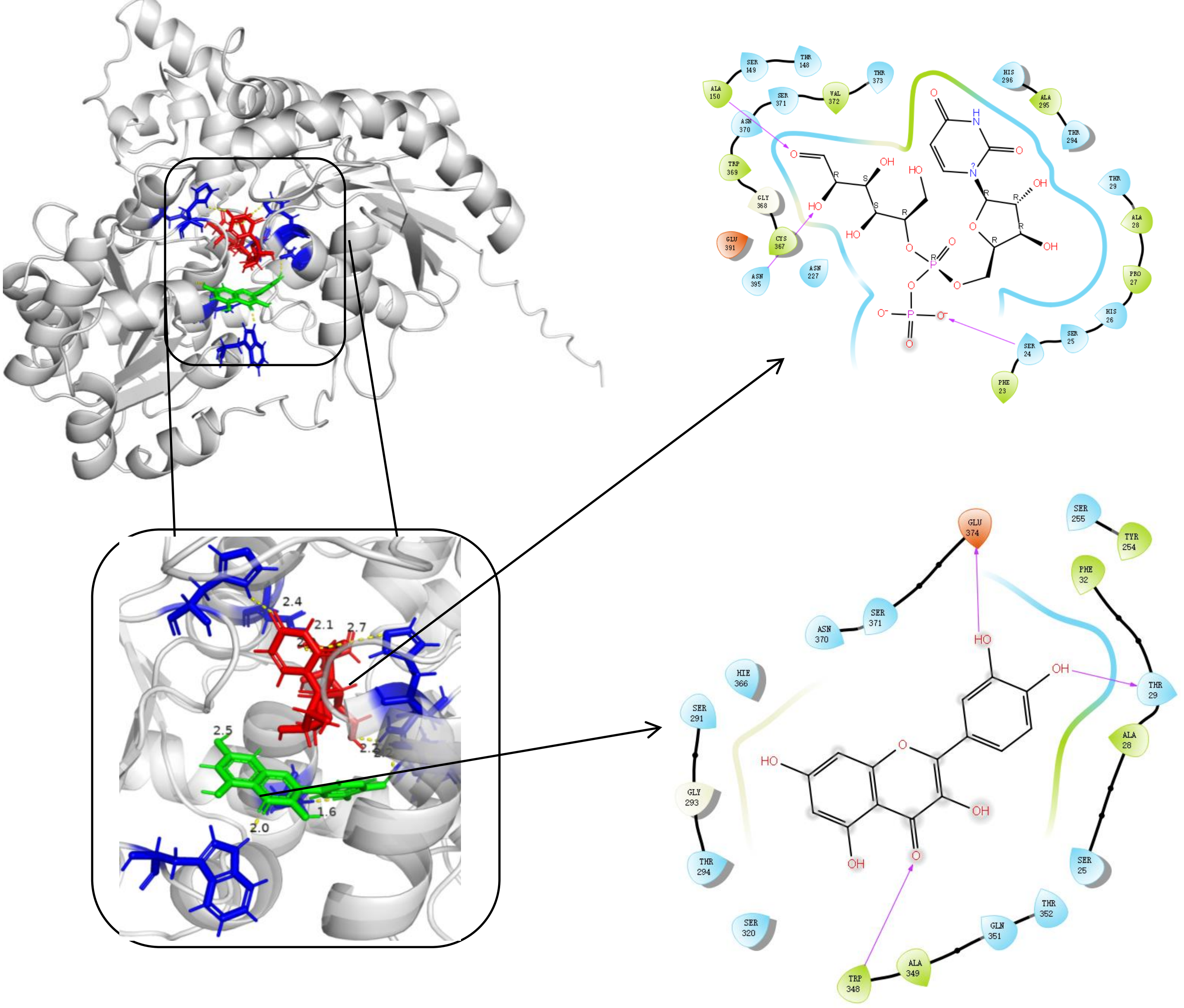

Supplemental Figure 24. Molecular docking simulation of FhUFGT3 with UDP-glucose and quercetin。

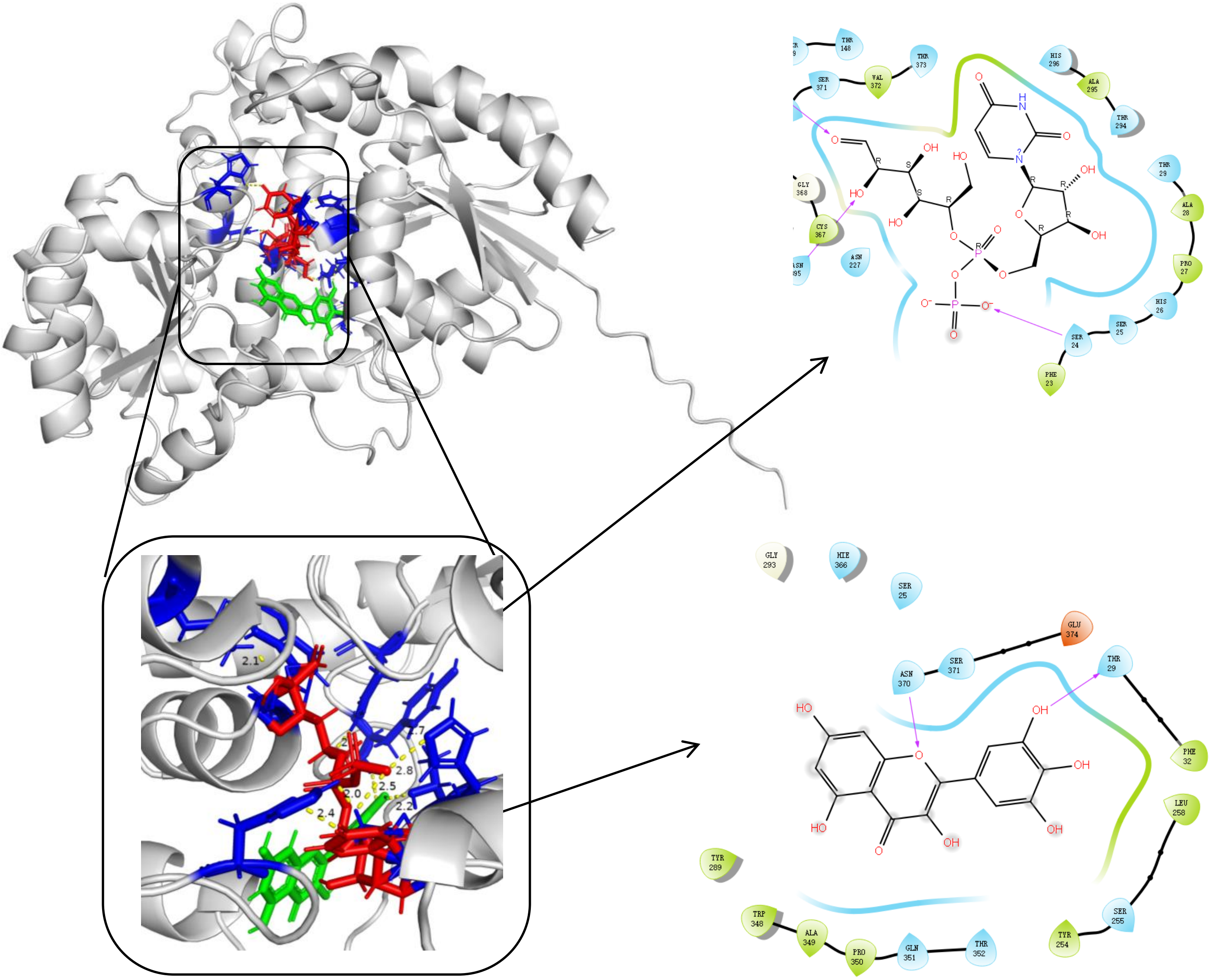

Supplemental Figure 25. Molecular docking simulation of FdUFGT3 with UDP-glucose and myricetin.

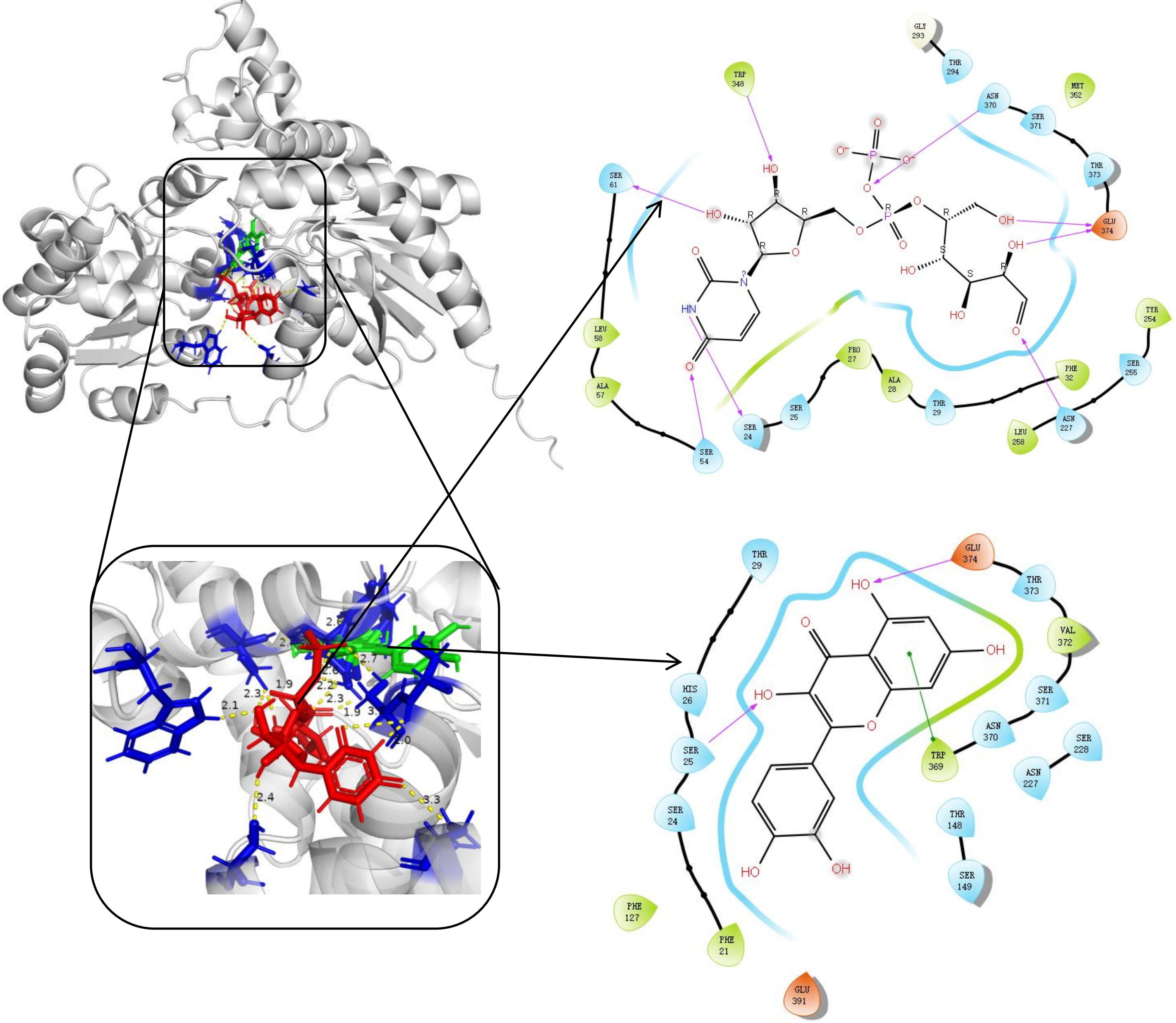

Supplemental Figure 26. Molecular docking simulation of FeUFGT3 with UDP-glucose and kaempferol.

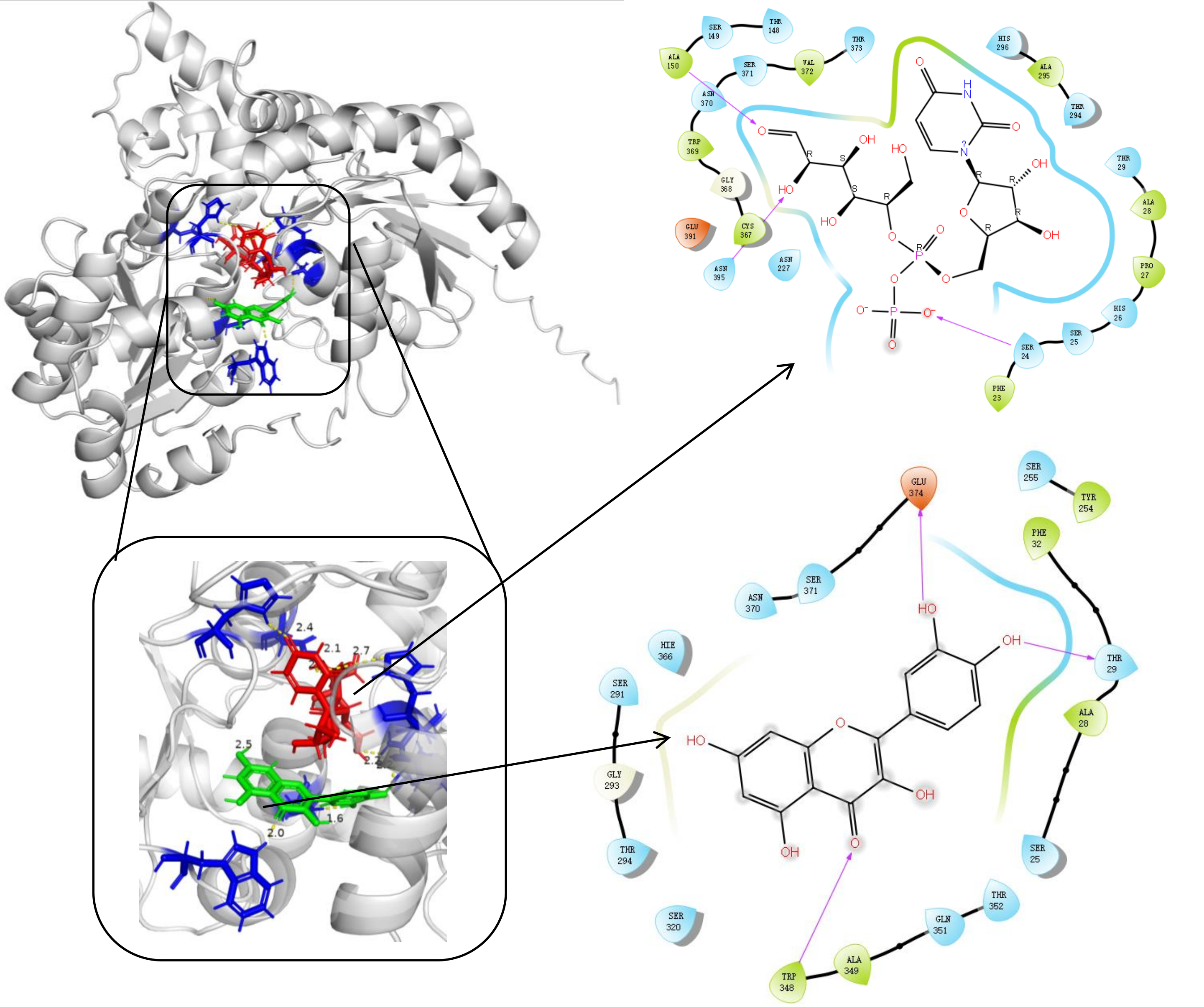

Supplemental Figure 27. Molecular docking simulation of FeUFGT3 with UDP-glucose and quercetin

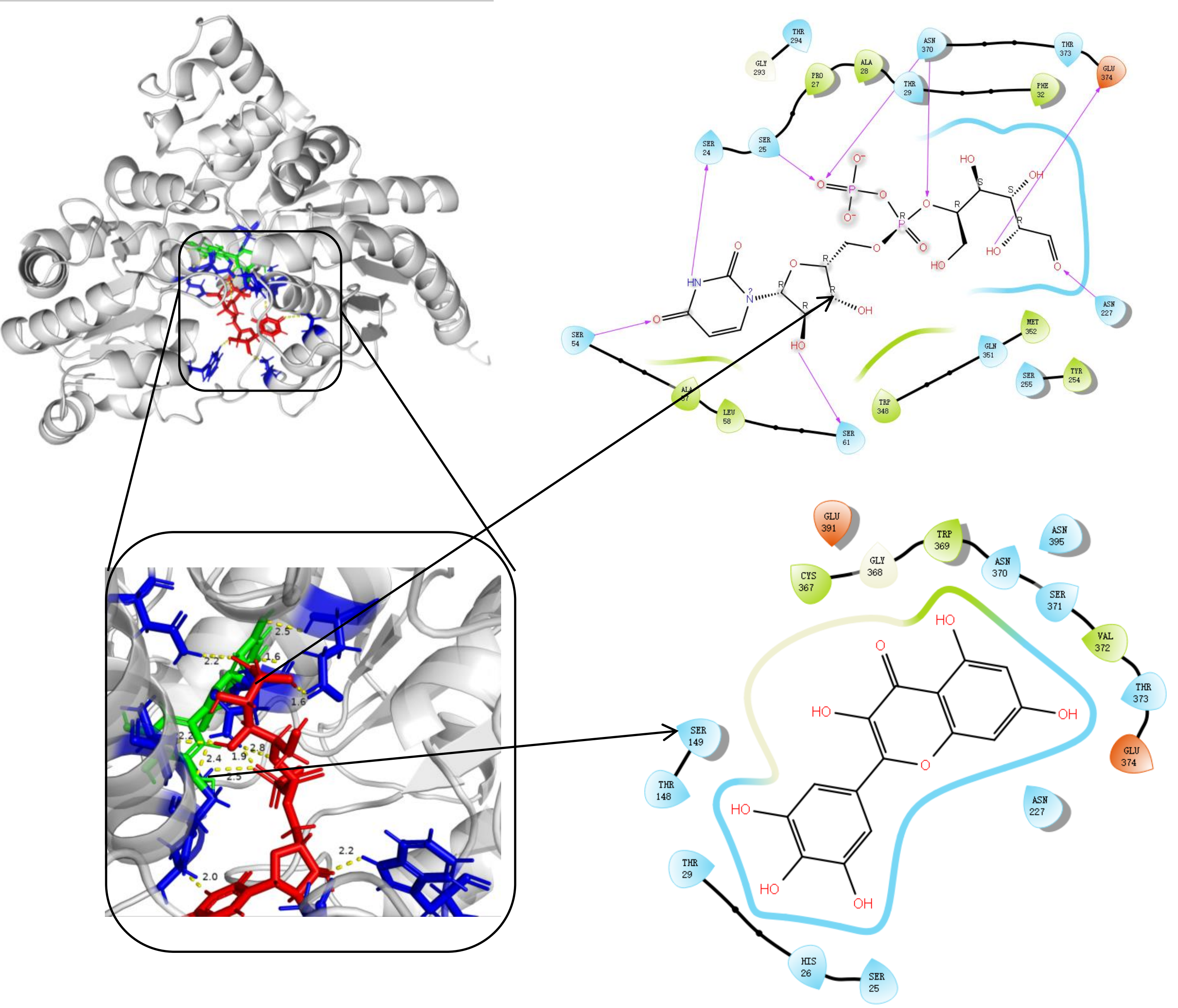

Supplemental Figure 28. Molecular docking simulation of FeUFGT3 with UDP-glucose and myricetin.

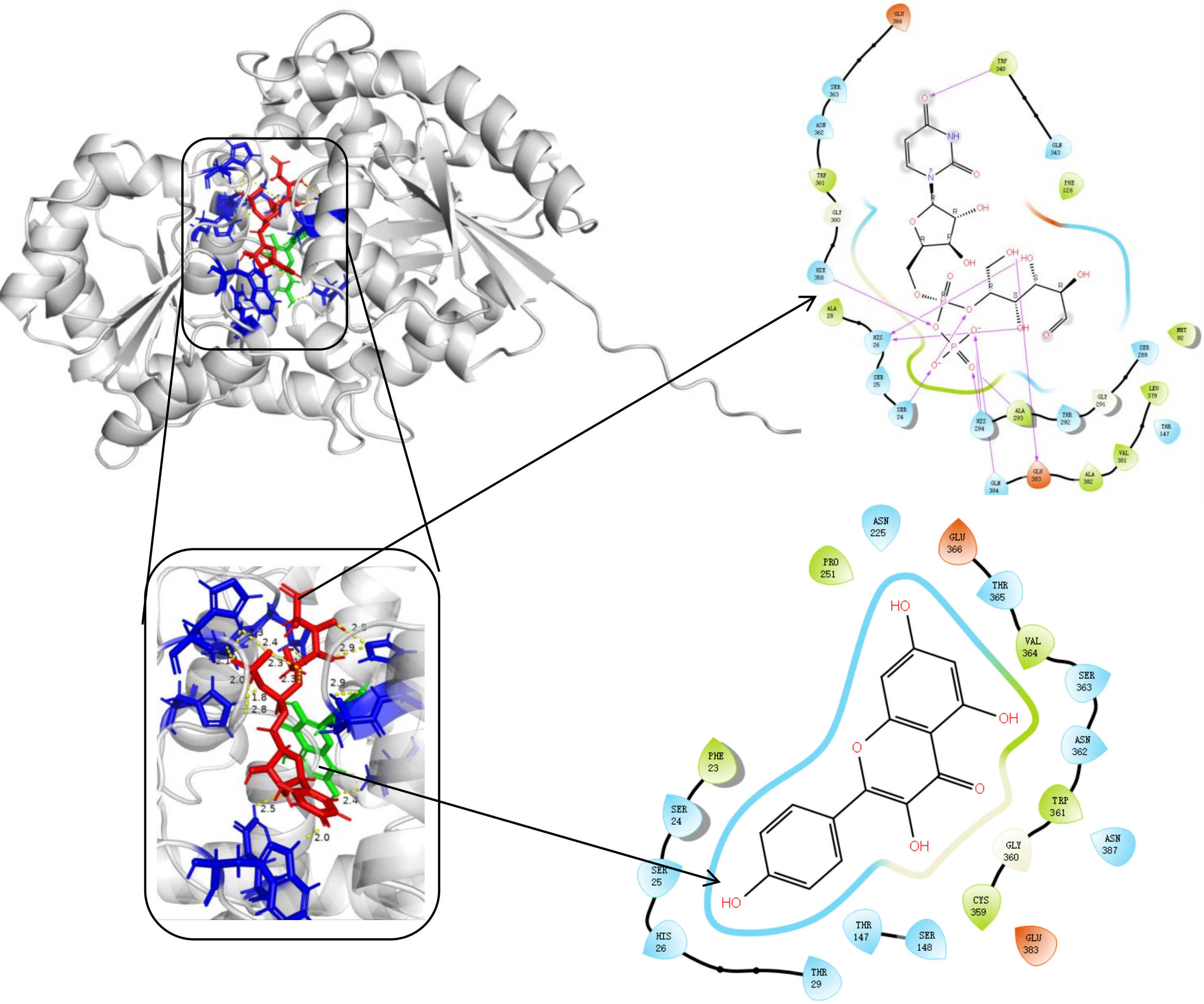

Supplemental Figure 29. Molecular docking simulation of FlUFGT3 with UDP-glucose and kaempferol.

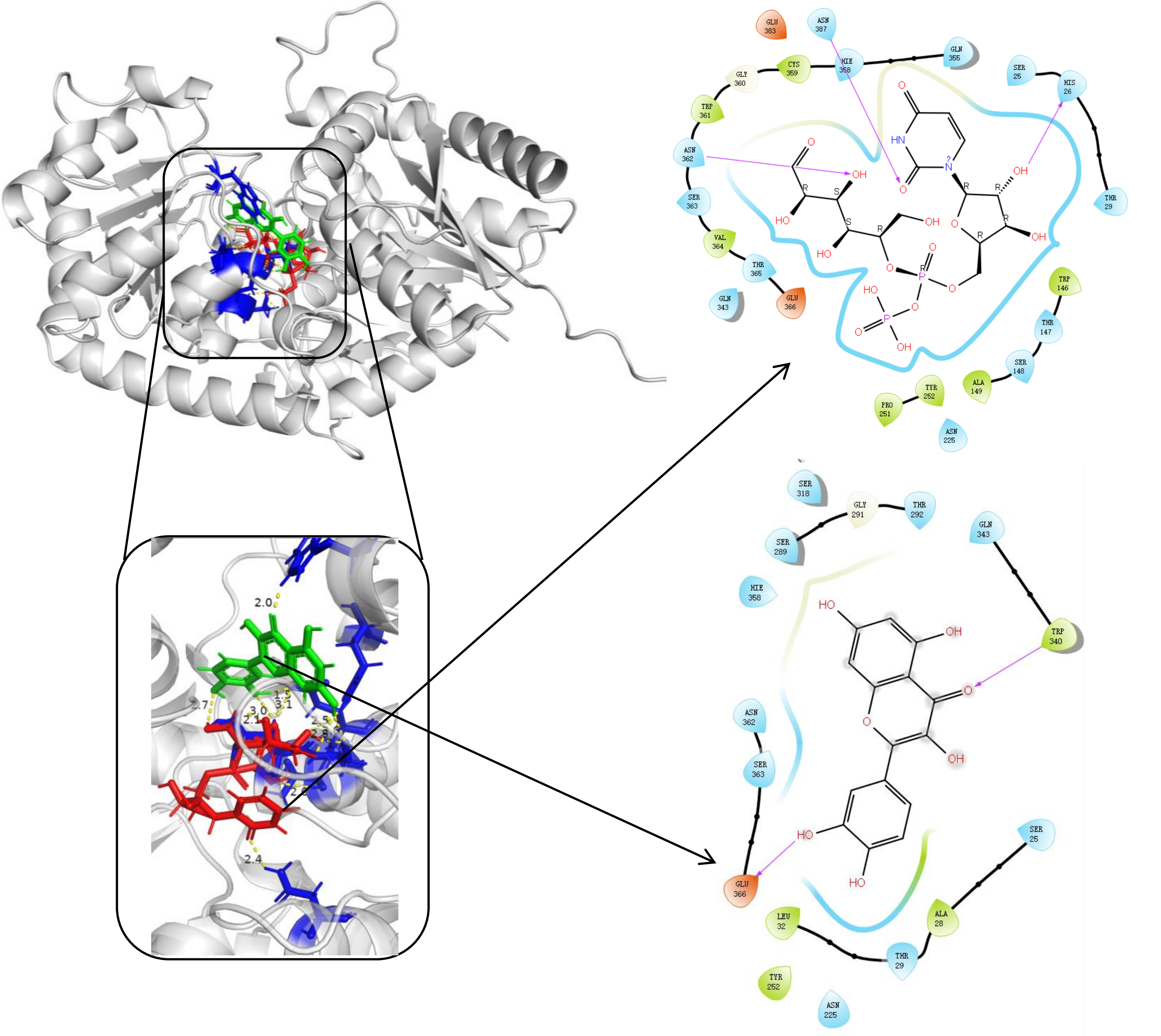

Supplemental Figure 30. Molecular docking simulation of FlUFGT3 with UDP-glucose and quercetin.

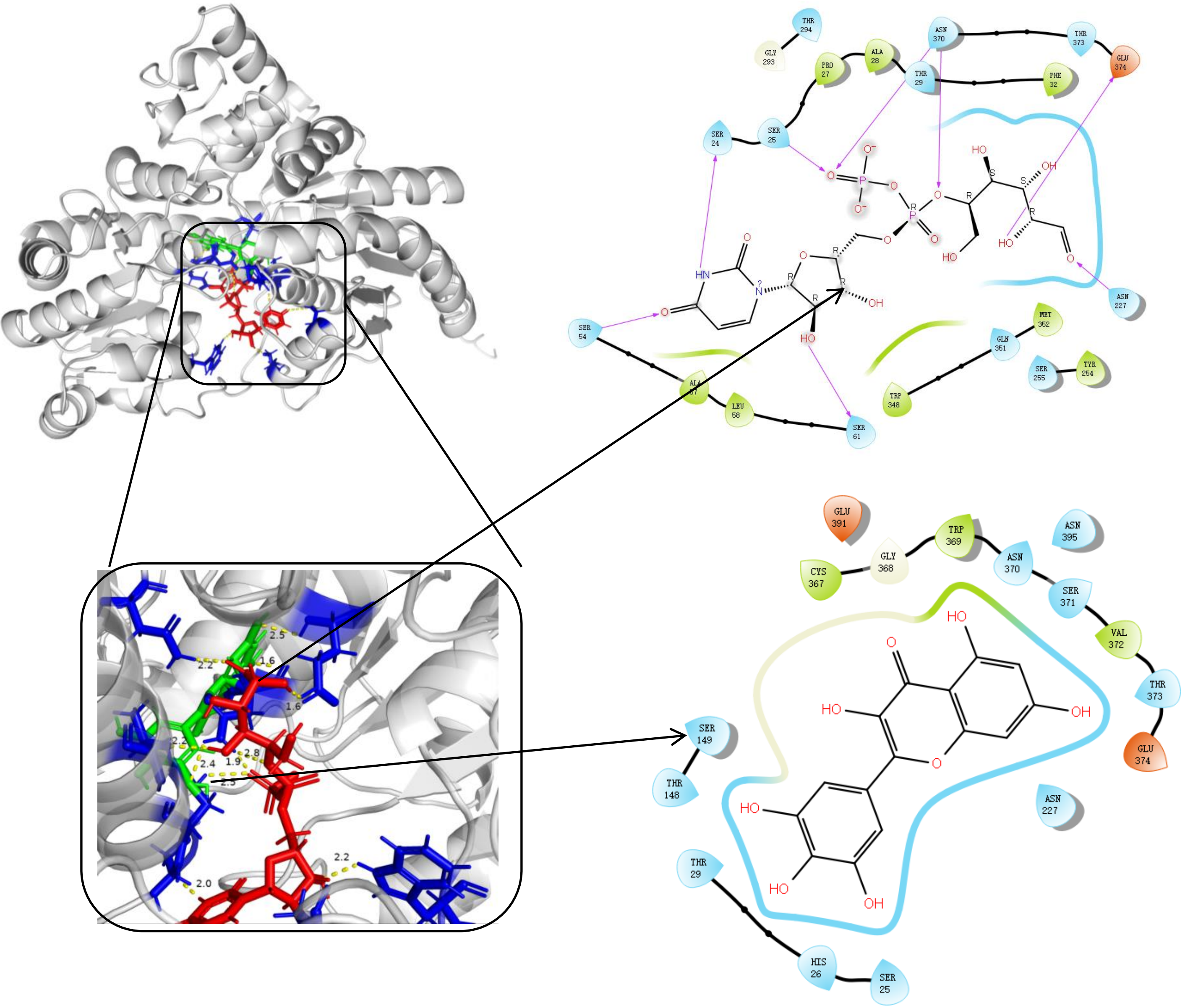

Supplemental Figure 31. Molecular docking simulation of FlUFGT3 with UDP-glucose and myricetin.

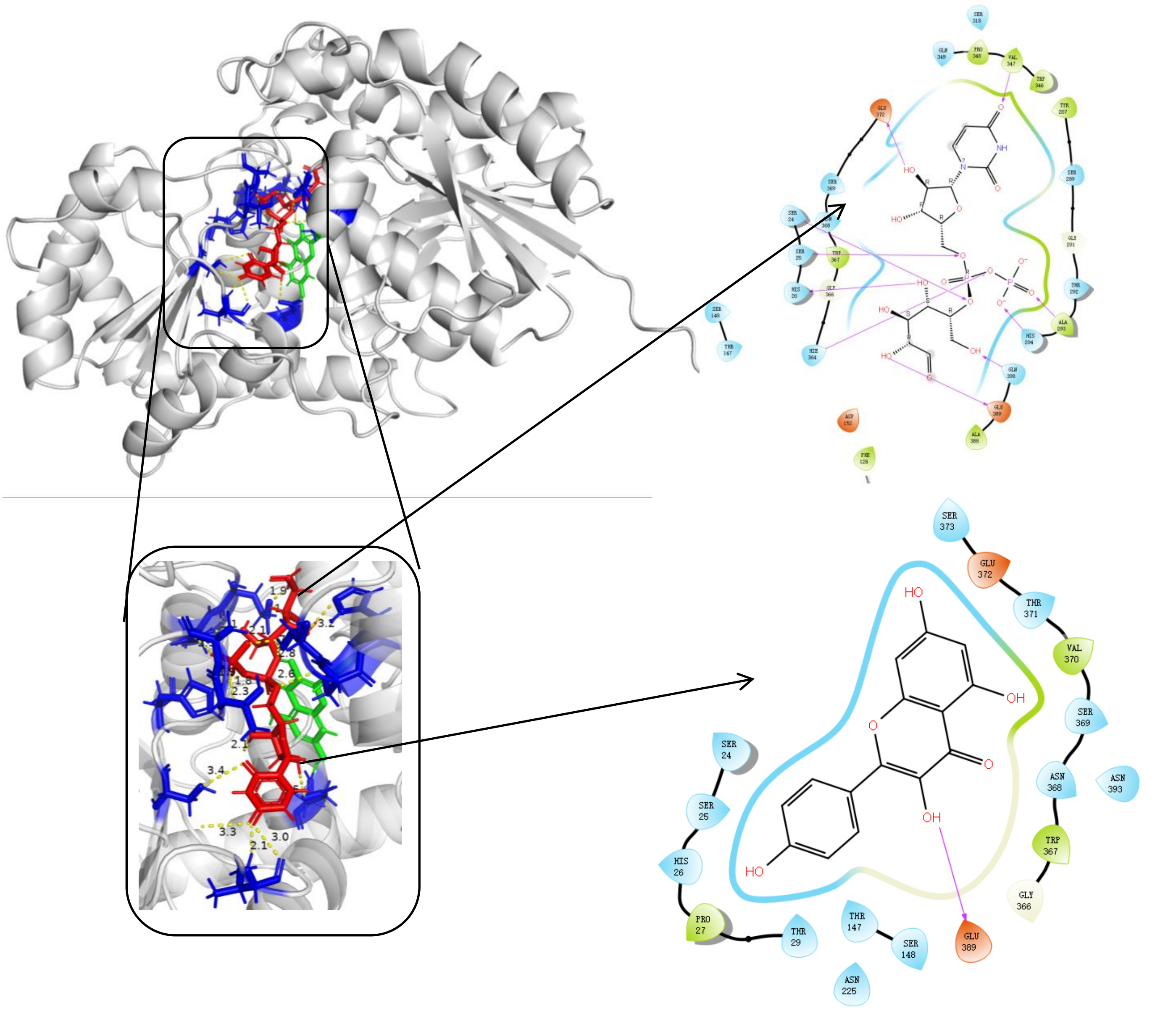

Supplemental Figure 32. Molecular docking simulation of FpUFGT3 with UDP-glucose and kaempferol.

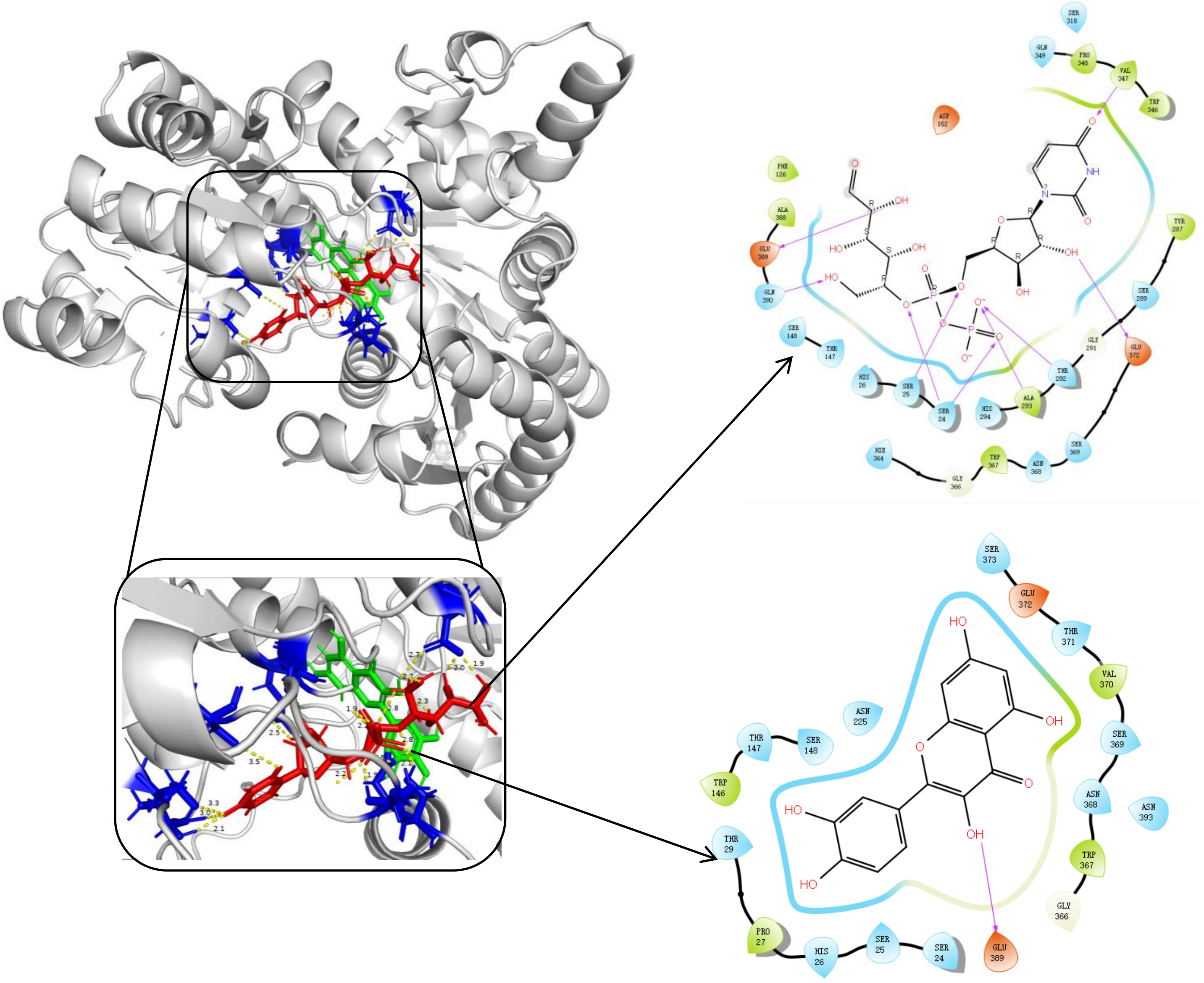

Supplemental Figure 33. Molecular docking Simulation of FpUFGT3 with UDP-glucose and quercetin

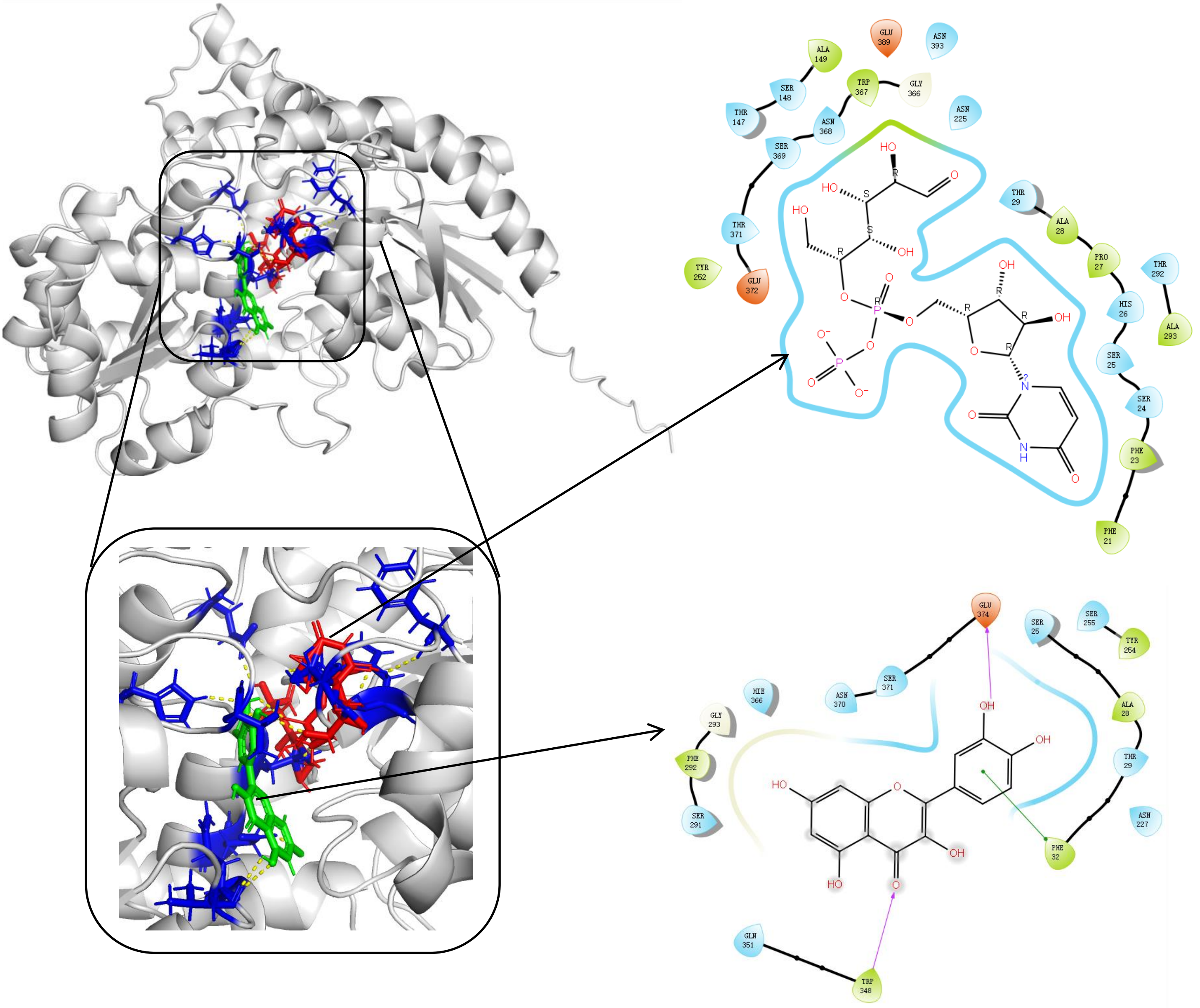

Supplemental Figure 34 Molecular docking simulation of FpUFGT3 with UDP-glucose and myricetin.

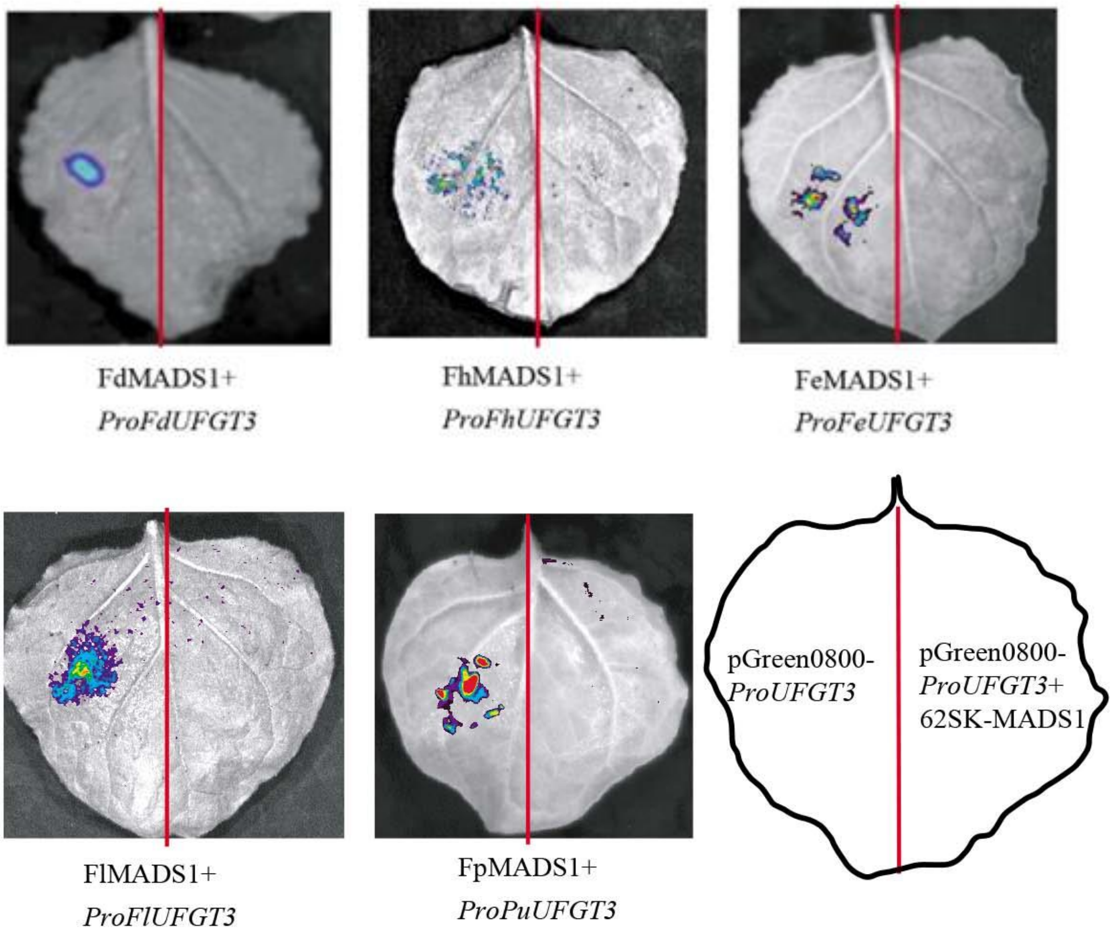

Supplemental Figure 35. Dual-luciferase assay results demonstrated the interaction between MADS1 and promoter of *UFGT3*. The fusion proteins were expressed in *N.benthamiana* using agroinfiltration. Chemiluminescence images were captured 36 hours after infiltration using 3mg/ml luciferin, and similar results were obtained in three biological replicates.

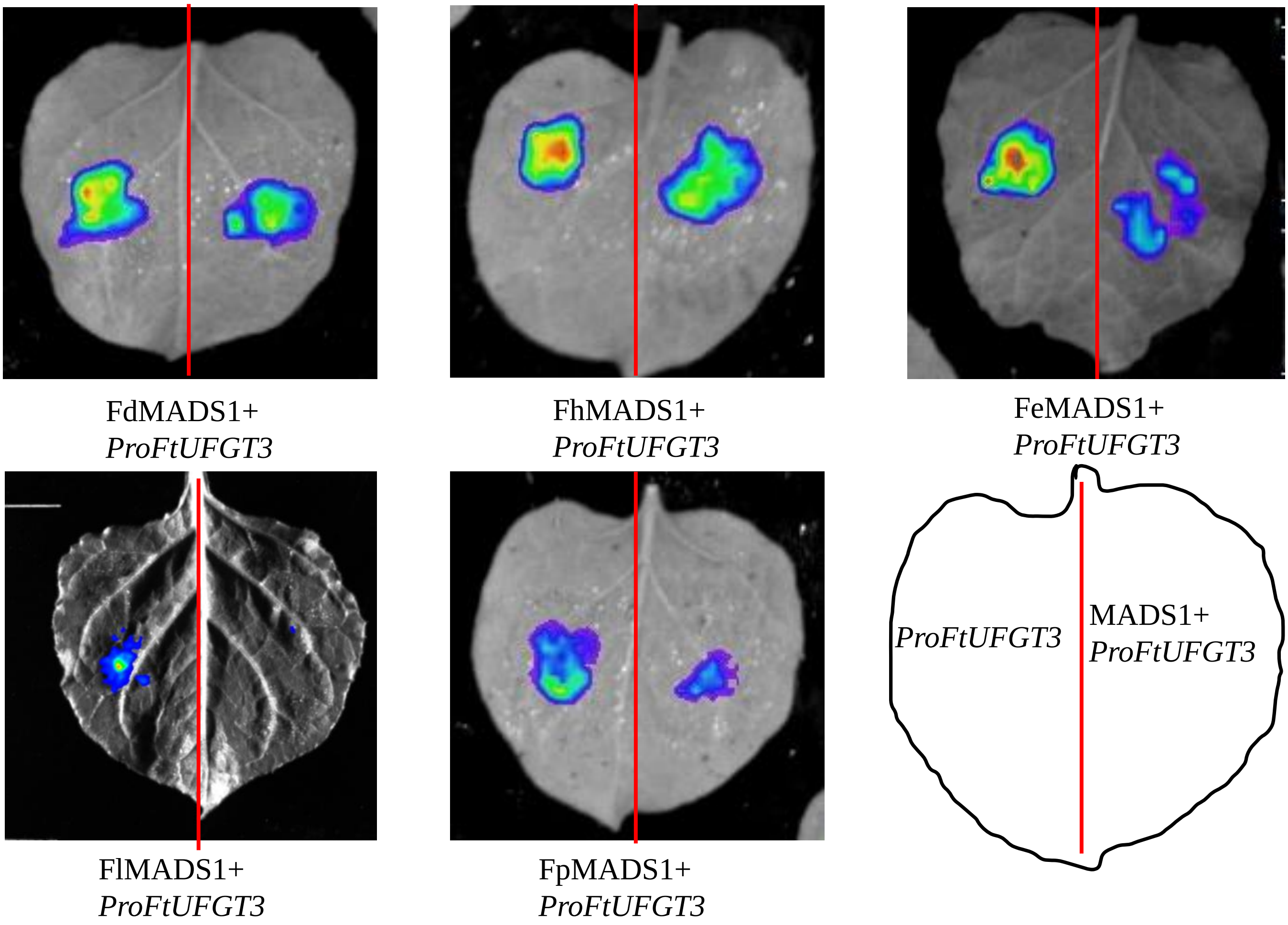

Supplemental Figure 36. Dual-luciferase reporter assays were used to detect the interaction between MADS1 and the *ProFtUFGT3* promoter of other species in the *Fagopyrum* genus.

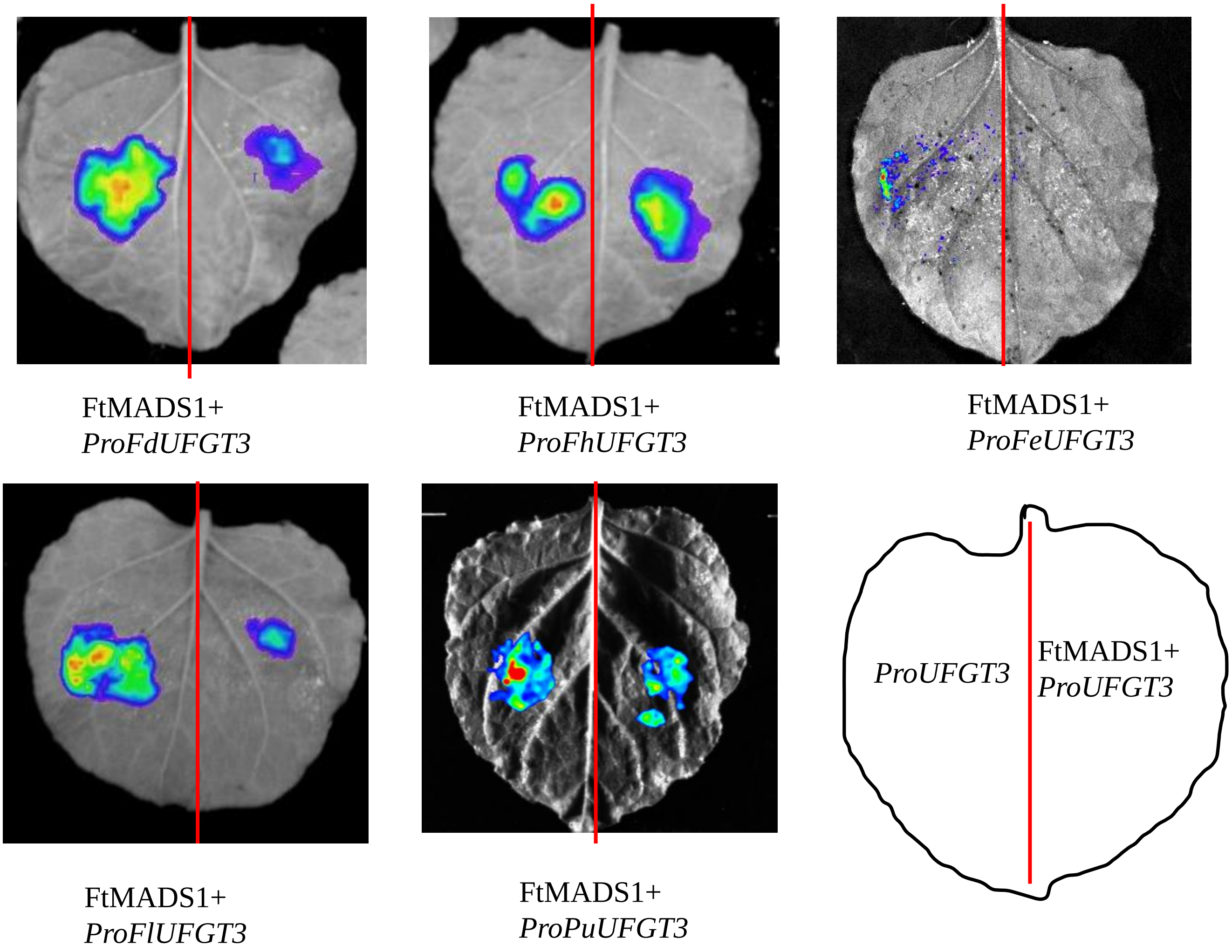

Supplemental Figure 37. Dual bioluminescence assay were used to detect the interaction between FtMADS1 and the *UFGT3* promoter of other species in the *Fagopyrum* genus.

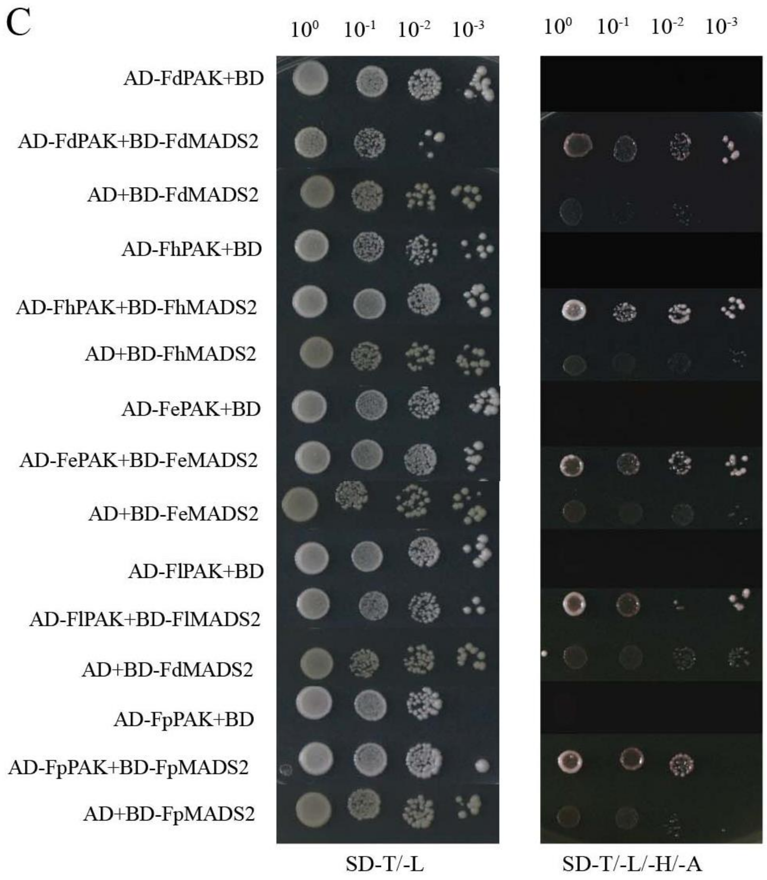

Supplemental Figure 38 A) The yeast two-hybrid (Y2H) results of the interaction between PAK and MADS2 in the Fagopyrum genus. SD-L/-T, SD basic medium lacking Leu and Trp; SD-L/-T/-H/-A, SD basal medium lacking Leu, Trp, His, and Ade.

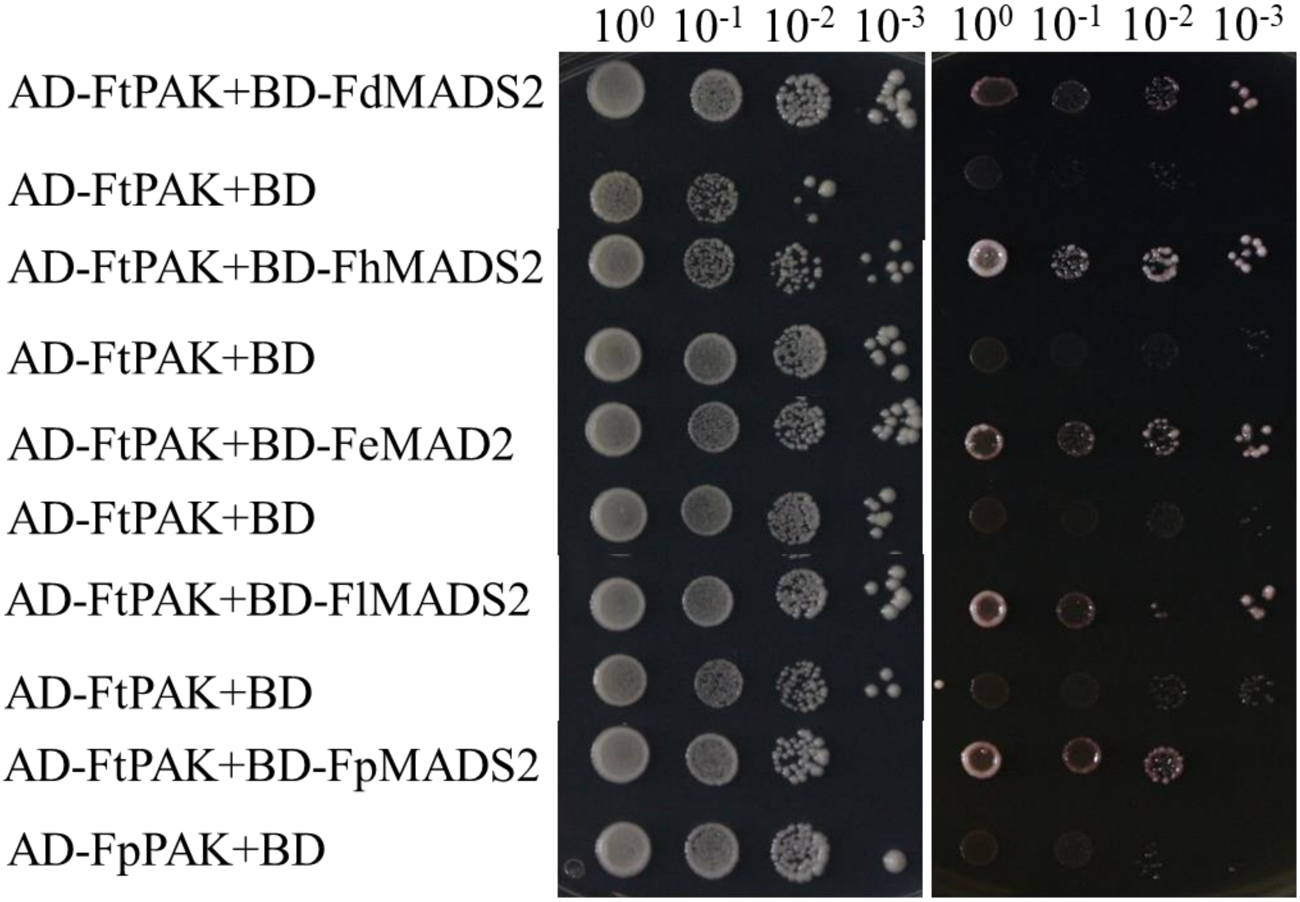

Supplemental Figure 39. Y2H assays were used to detect the interaction between FtPAK and the MADS2 of other species in the *Fagopyrum* genus.

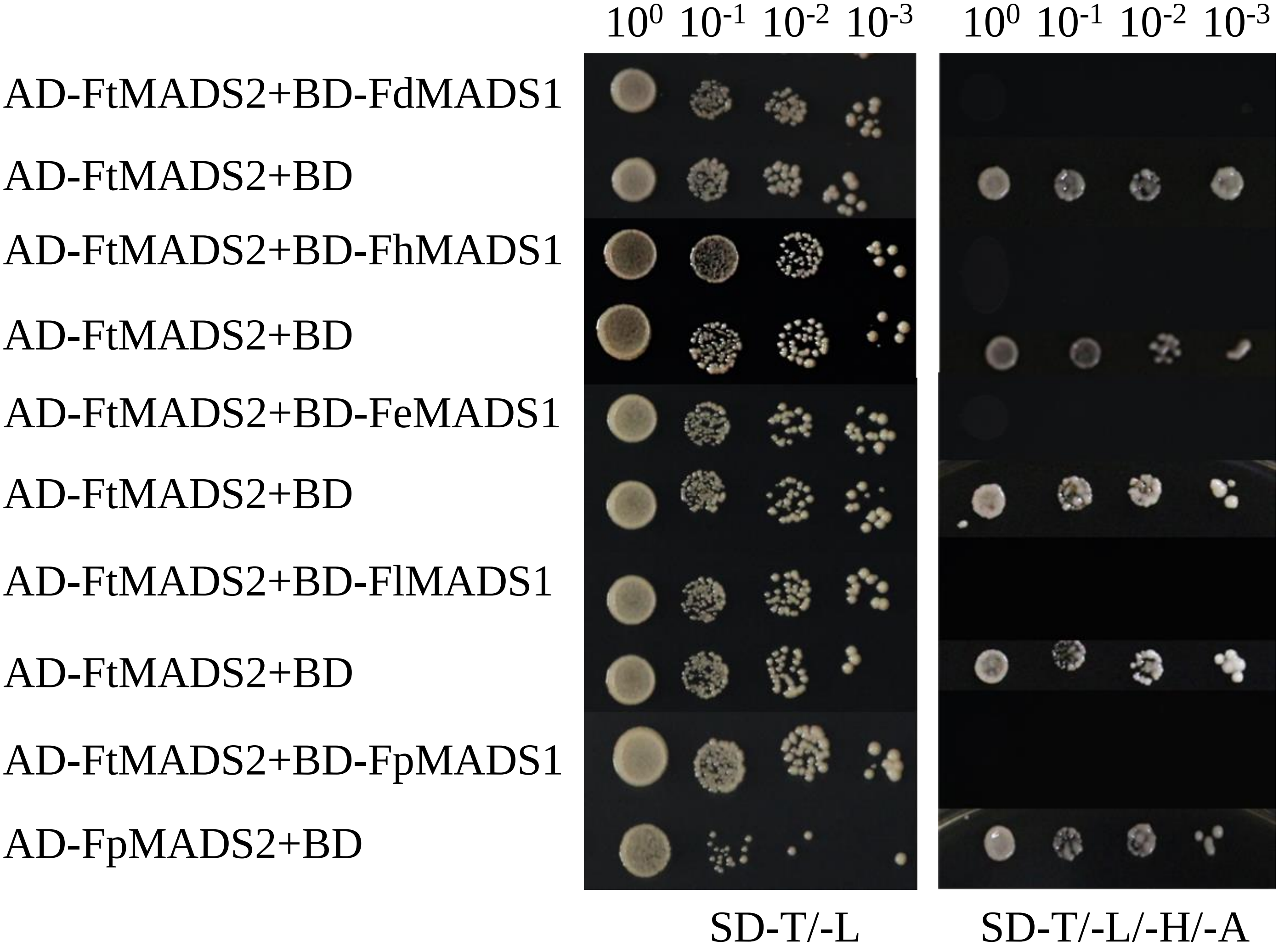

Supplemental Figure 40. Y2H assays were used to detect the interaction between FtPAK and the MADS2 of other species in the *Fagopyrum genus*.

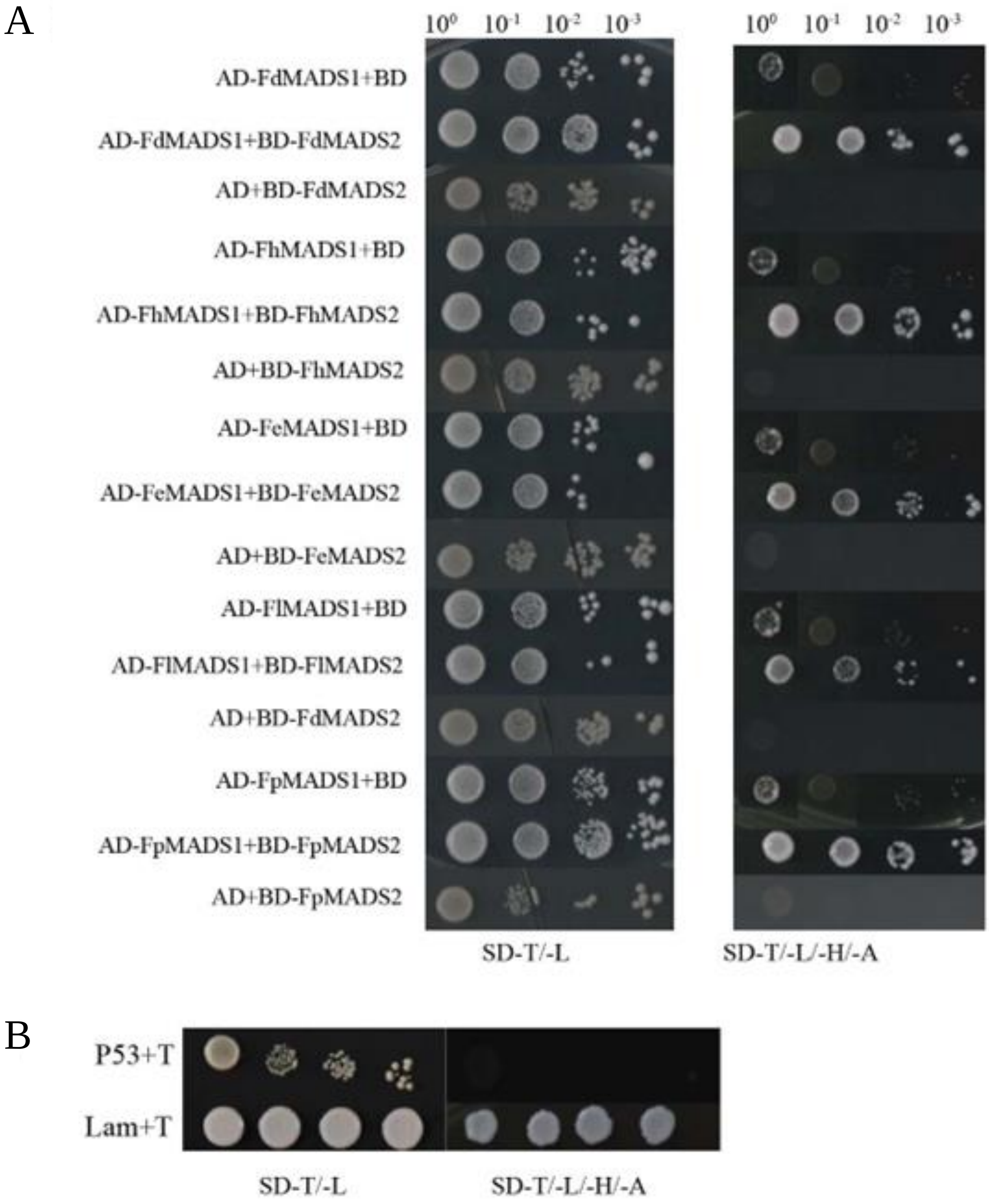

Supplemental Figure 41. A) The yeast two-hybrid (Y2H) results of the interaction between MADS2 and MADS1 in the Fagopyrum genus. SD-L/-T, SD basic medium lacking Leu and Trp; SD-L/-T/-H/-A, SD basal medium lacking Leu, Trp, His, and Ade. **B)** P53+T denotes the positive control for the Y2H, while Lam+L denotes the negative control.

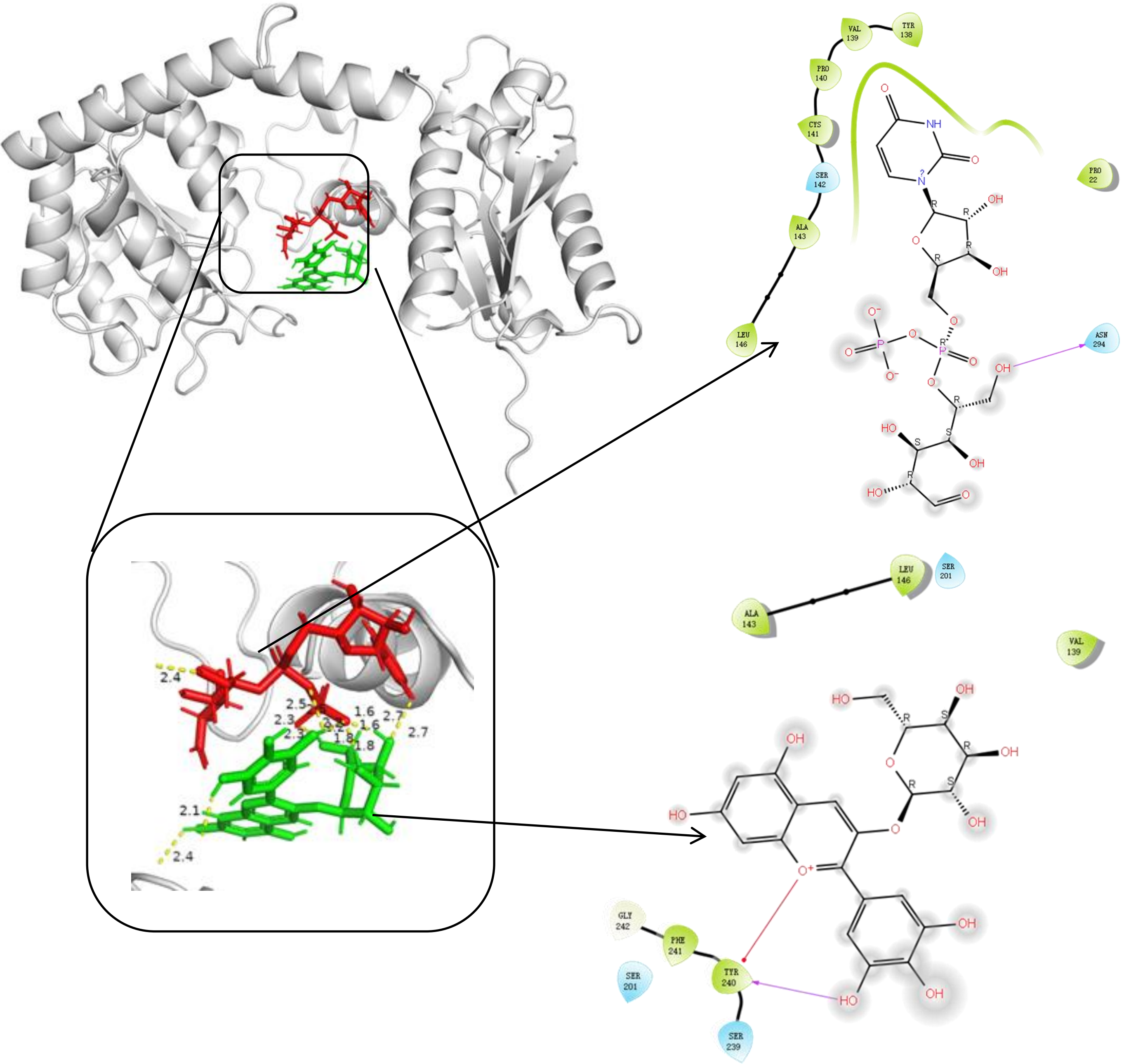

Supplemental Figure 42. Molecular docking simulation of FlAGT with UDP-glucose and cyanidin.

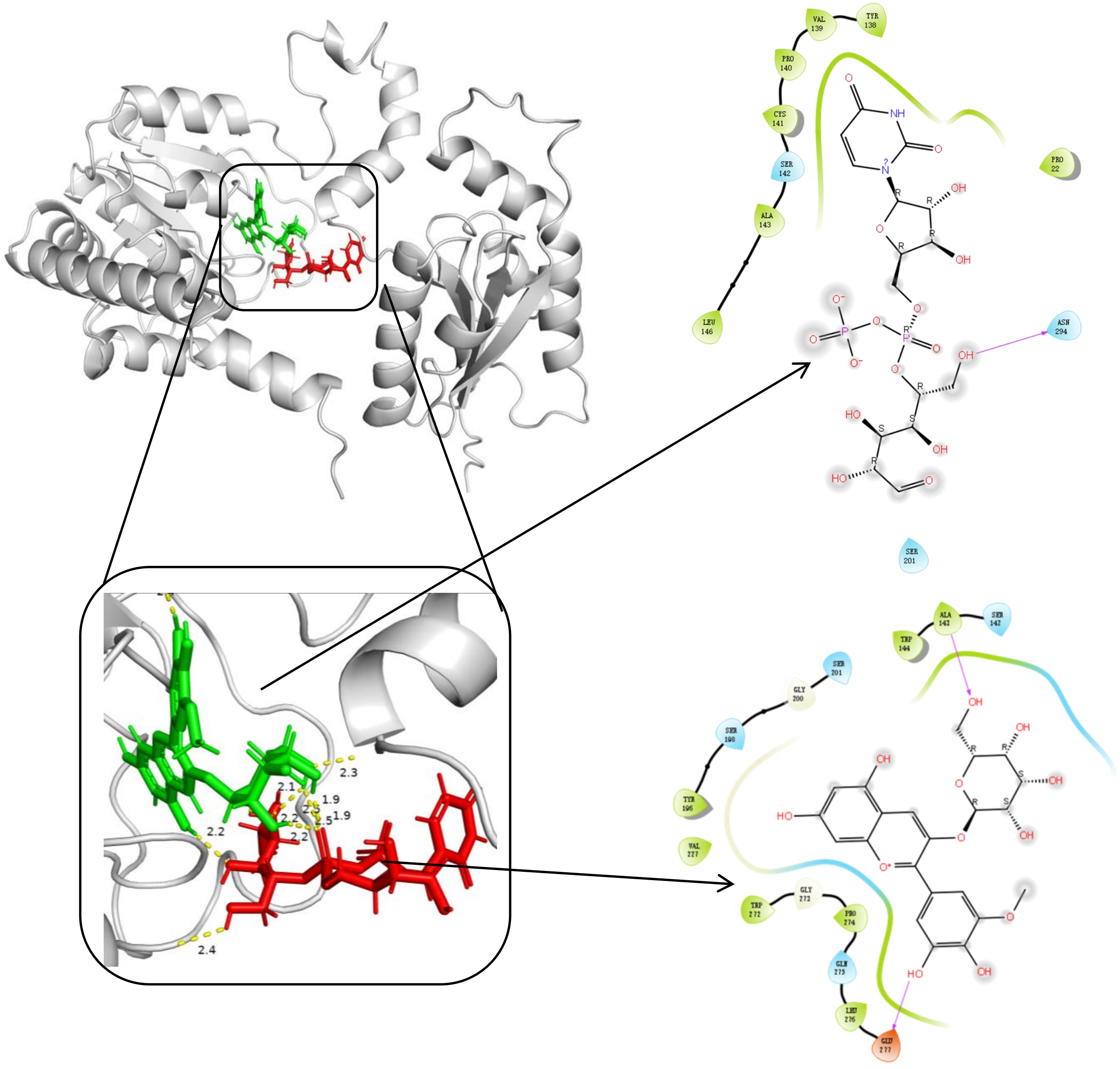

Supplemental Figure 43. Molecular docking simulation of FlAGT with UDP-glucose and petunidol.

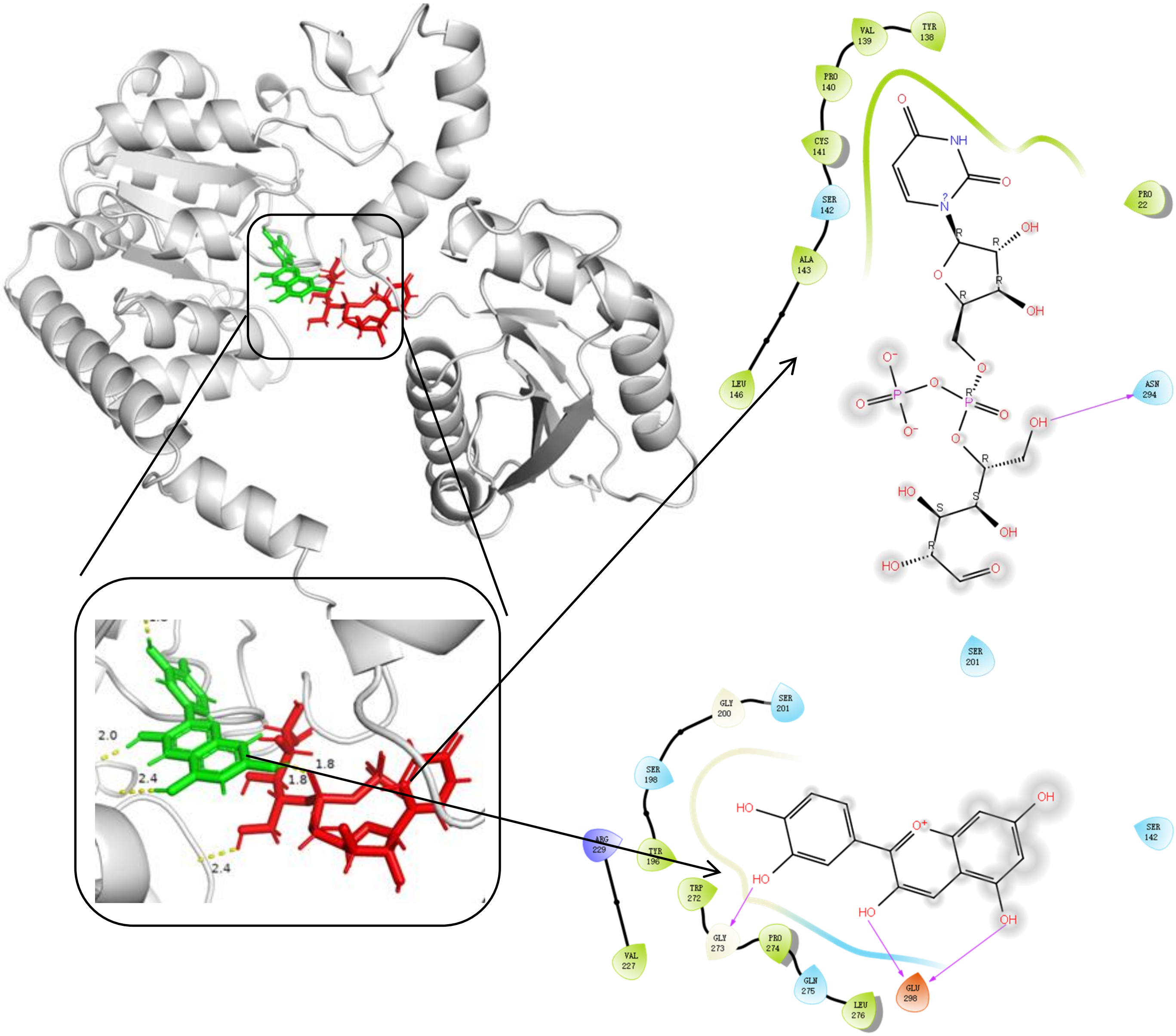

Supplemental Figure 44. Molecular docking simulation of FlAGT with UDP-glucose and delphinidin.

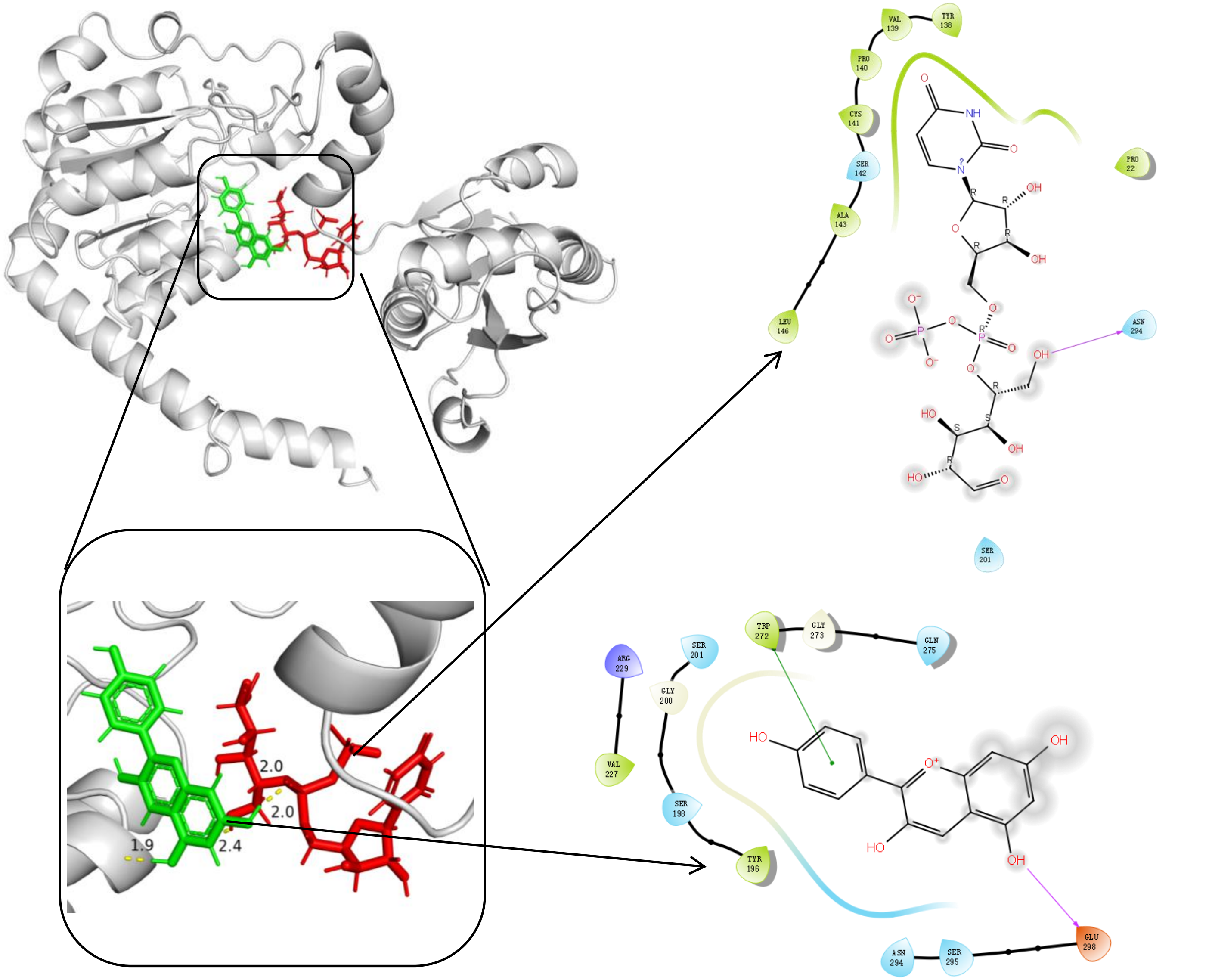

Supplemental Figure 45. Molecular docking simulation of FlAGT with UDP-glucose and pelargonidin.

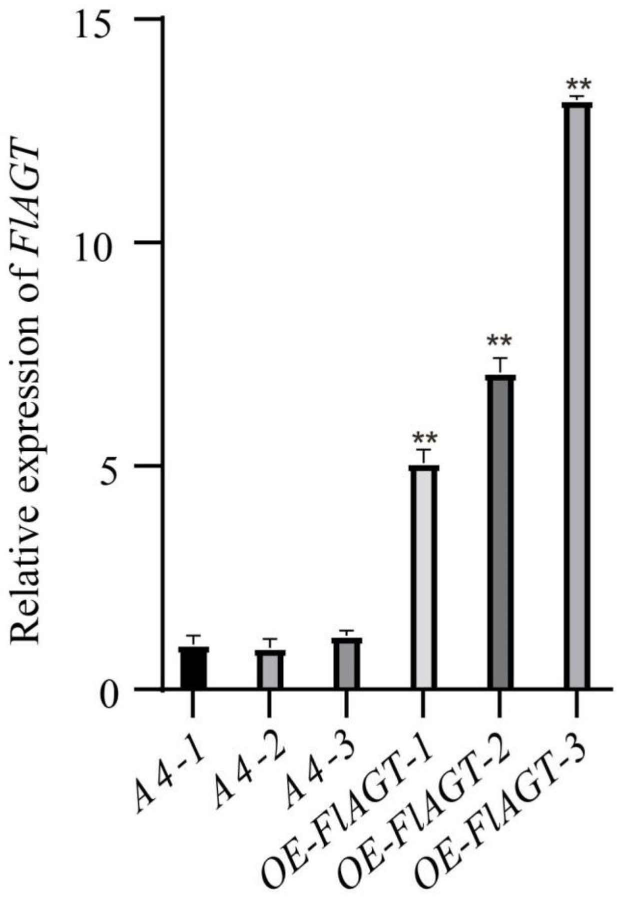

Supplemental Figure 46. Relative expression level of *FlAGT* in hairy roots with *FlAGT* overexpression

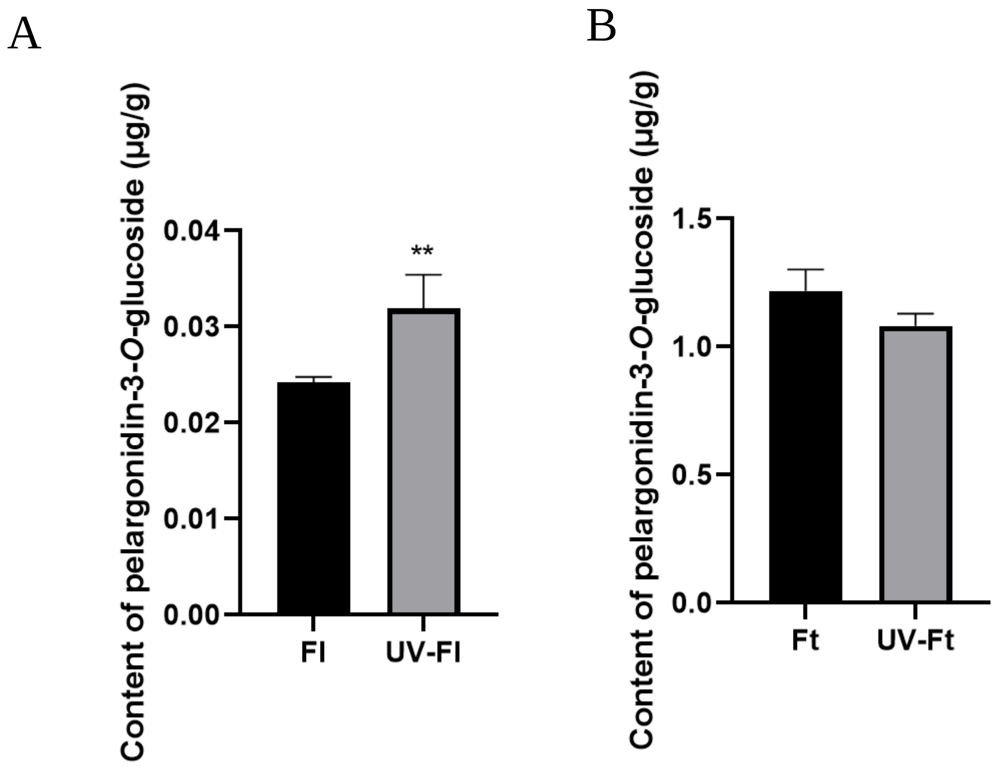

Supplemental Figure 47. Effect of UV-B on pelargonidin-3-*O*-glucoside content.

A) Effect of UV-B on pelargonidin-3-*O*-glucoside content in *Fagopyrum longistylum*.

B) Effect of UV-B on pelargonidin-3-*O*-glucoside content in Tartary buckwheat.

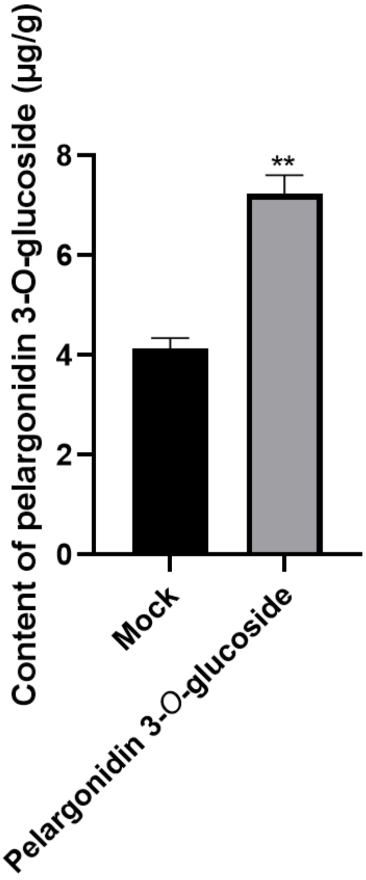

Supplemental Figure 48. Pelargonidin-3-*O*-glucoside can enter the plant through foliar spray. Spraying 2 mg/L pelargonidin 3-*O*-glucoside on the Tartary buckwheat leaves, water was sprayed in the mock group. Detect the content of pelargonidin 3-*O*-glucoside in plants after 1 h.

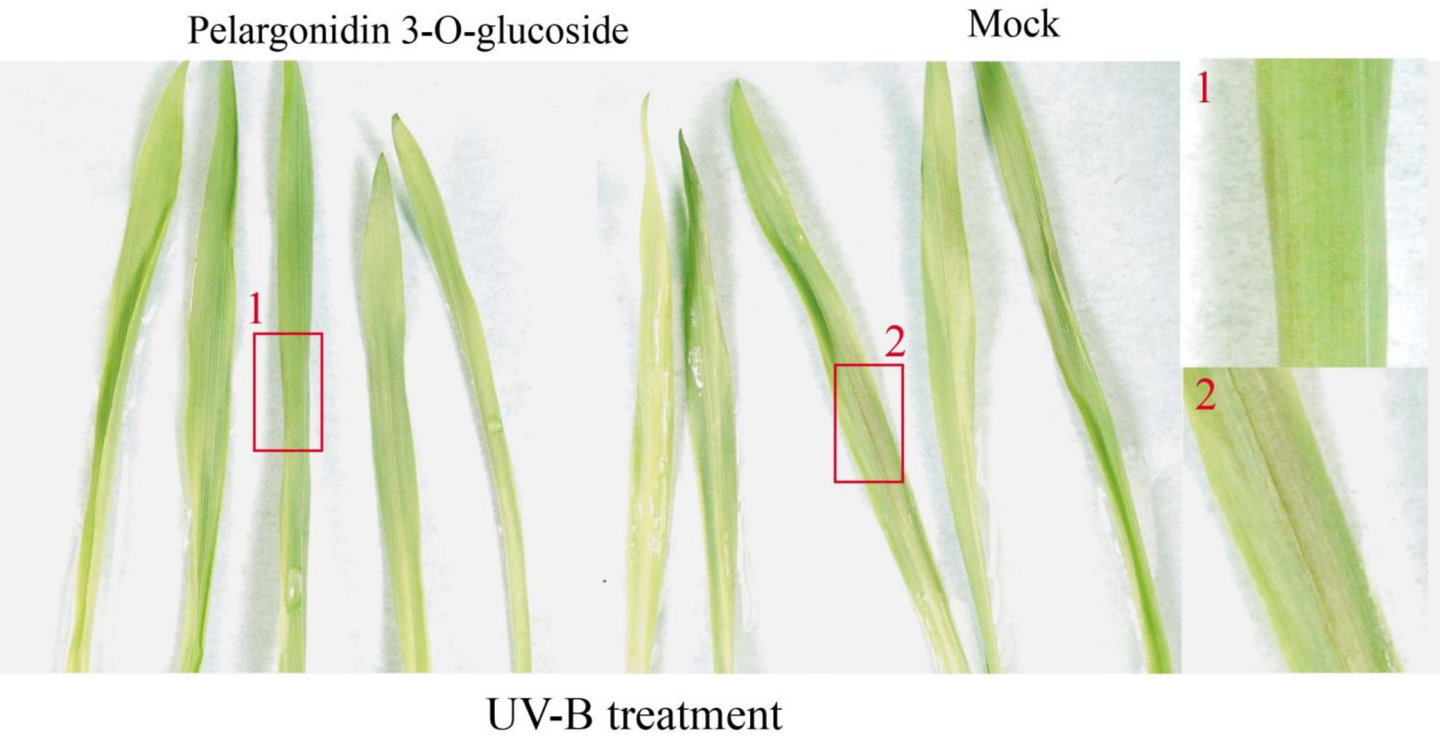

Supplemental Figure 49. Spraying 2 mg/L pelargonidin 3-*O*-glucoside on the *Hordeum vulgare* L. leaves, water was sprayed in the mock group. UV-B irradiation caused rust spots on the leaves, and the red boxes with numbers represent the enlarged areas corresponding to the numbers on the right.

A

Pelargonidin 3-*O*-glucoside Mock

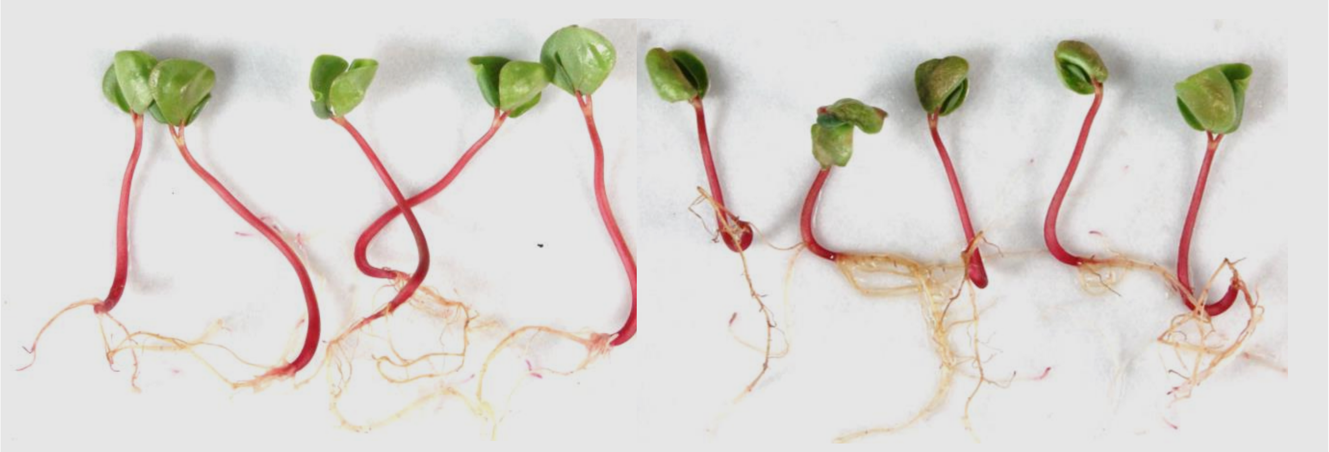

UV-B treatment

B

Pelargonidin 3-*O*-glucoside

Mock

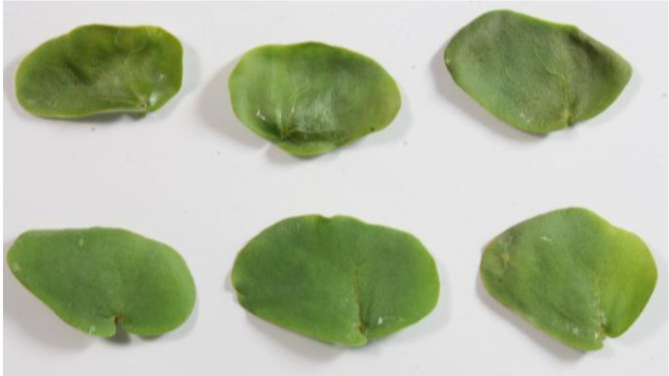

UV-B treatment

Supplemental Figure 50. Spraying 2 mg/L pelargonidin 3-*O*-glucoside on the *F.homotropicum*, water was sprayed in the mock group. UV-B irradiation caused rust spots on the leaves. A) Seedlings. B) Fully expanded mature cotyledons.

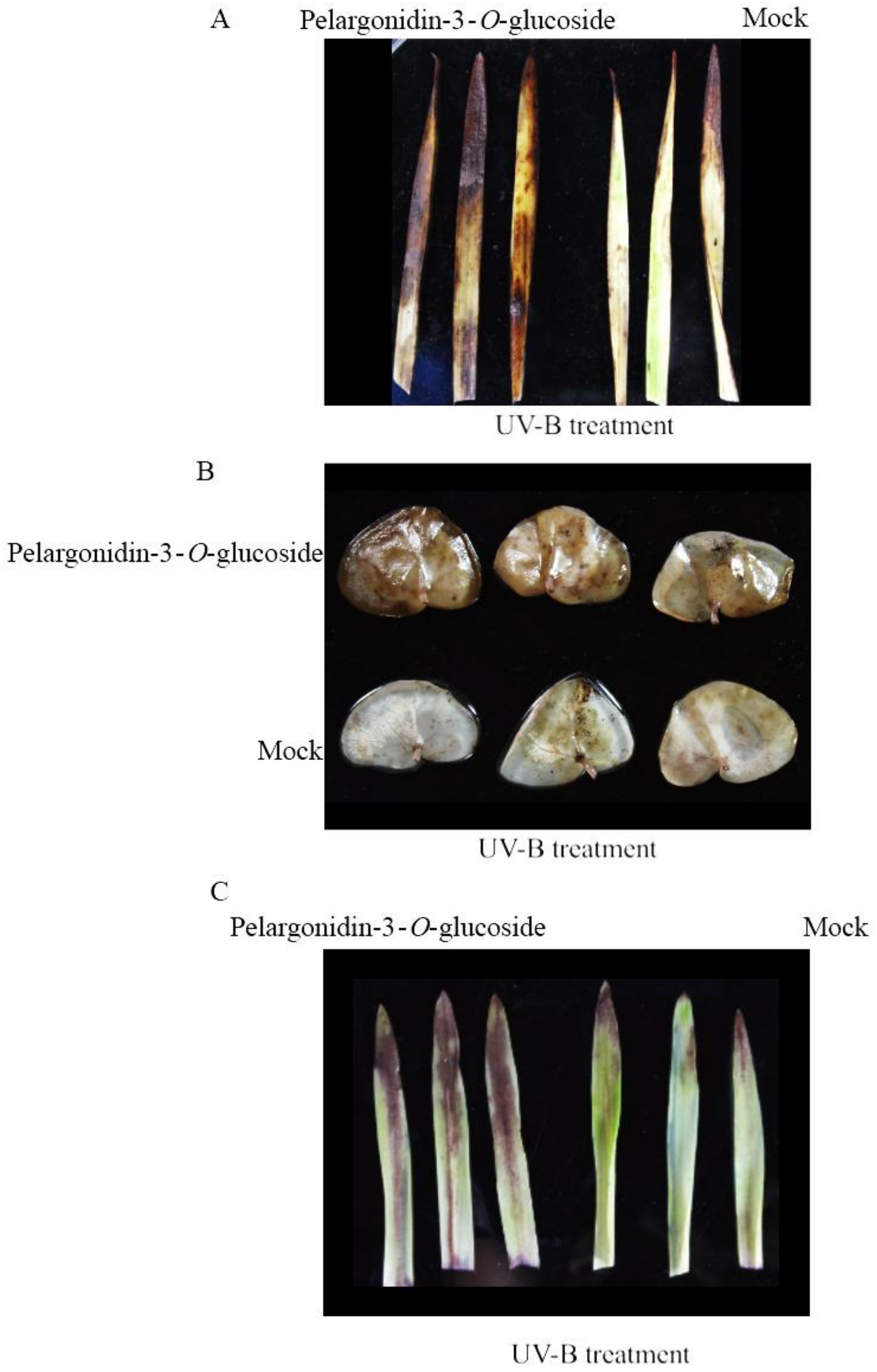

Supplemental Figure 51. After UV-B treatment, DAB was used for visual staining to assess plant damage before and after treatment with pelargonidin-3-*O*-glucoside. A) Staining of wheat tender leaves with DAB. B) Staining of *Hordeum vulgare L.* leaves with DAB. C) Staining of *F. homotropicum* leaves with DAB. All experiments were conducted with water as the mock.

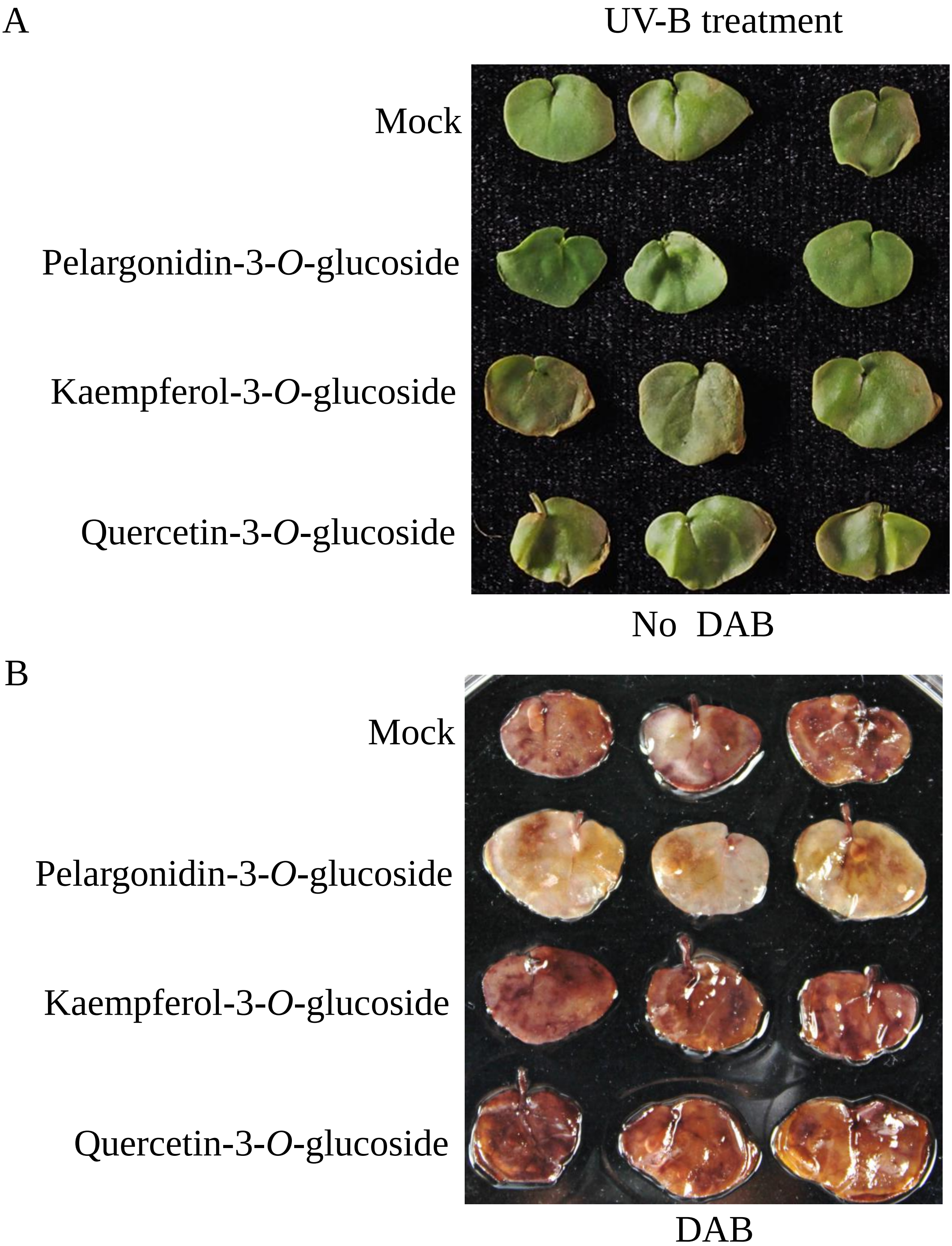

Supplemental Figure 52. After UV-B treatment, DAB was used for visual staining to assess plant damage before and after treatment with pelargonidin-3-*O*-glucoside,kaempferol-3-*O*-glucoside and quercetin-3-*O*-glucoside on buckwheat A) Before DAB staining. B) After DAB staining. All experiments were conducted with water as the mock.

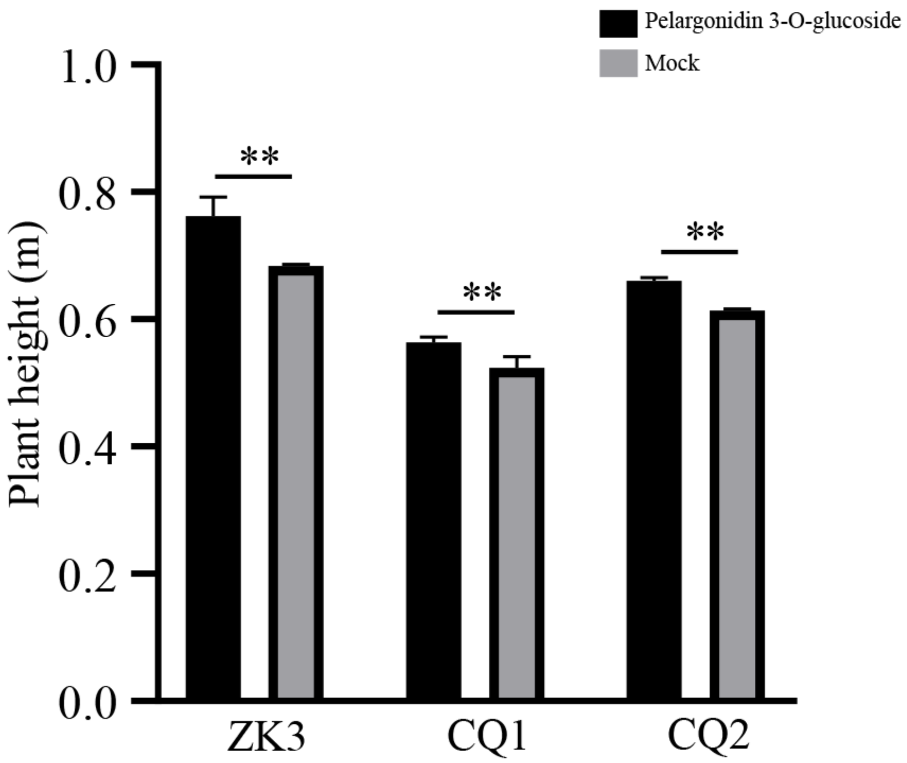

Supplemental Figure 53. In the area situated at an altitude of 3500 meters, three varieties of Tartary Buckwheat (ZK3, CQ1, CQ2) were subjected to treatment with 2 mg/L of Pelargonidin 3-O-glucoside, and the heights of the plants were measured. The treatment began at the emergence of seedlings, with spraying conducted every 15 days. Each group contained 100 plants, with three replications for each treatment.

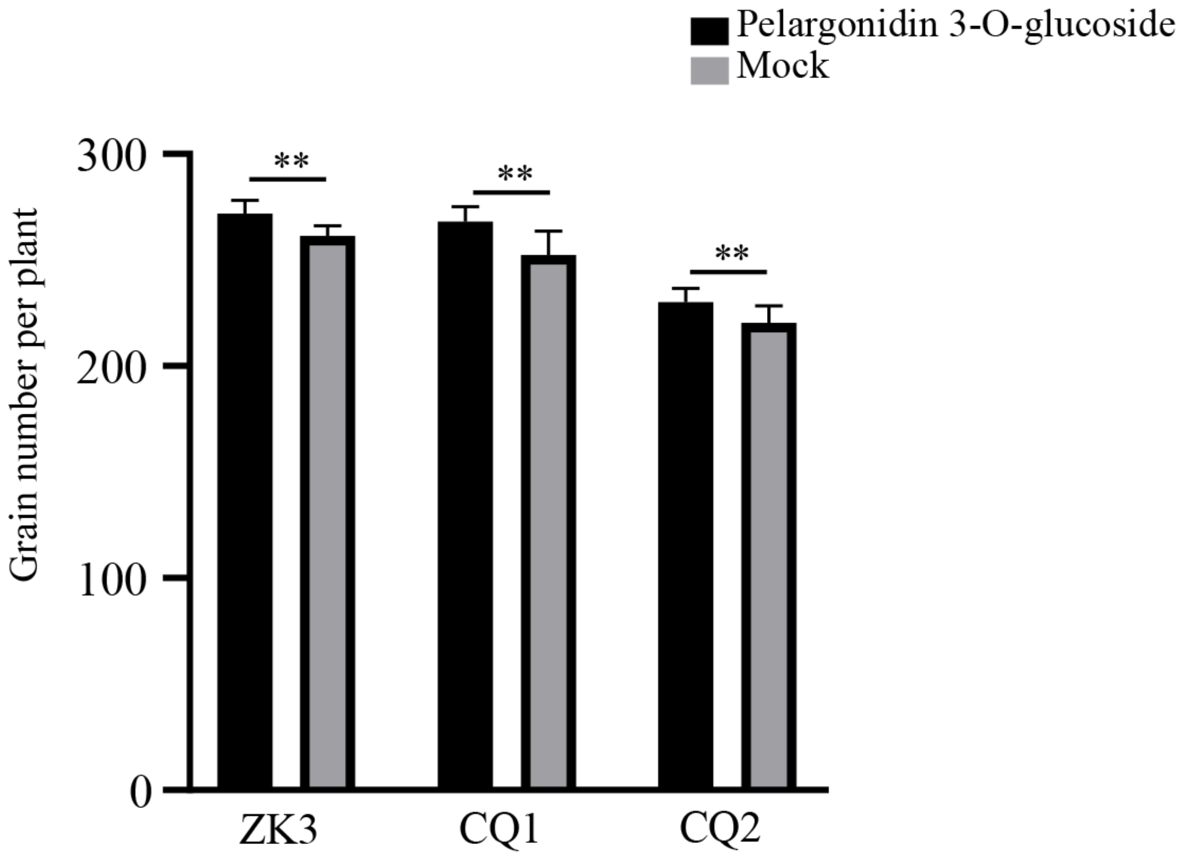

Supplemental Figure 54. In the area situated at an altitude of 3500 meters, three cultivar of Tartary buckwheat (ZK3, CQ1, CQ2) were subjected to treatment with 2 mg/L of Pelargonidin 3-*O*-glucoside, and the grain count per plant was documented. The treatment commenced at the emergence of seedlings, with spraying conducted at 15-day intervals. Each group comprised 100 plants, and each treatment was replicated 3 times.

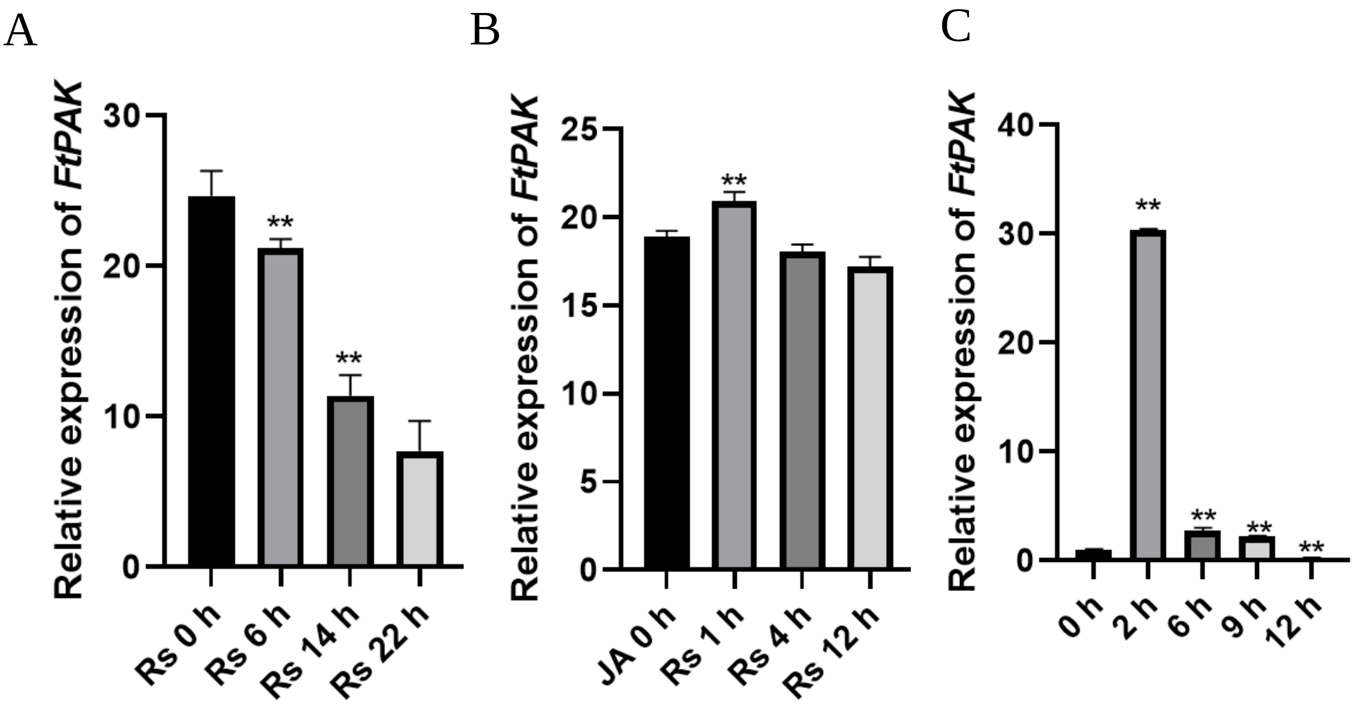

Supplemental Figure 55. Expression levels of *FtPAK* under different conditions. A) Expression levels of *FtPAK* at different time points after *Rhizoctonia solani* infection in the transcriptome. B) Expression levels of *FtPAK* at different time points after JA treatment in the transcriptome. C) Relative expression levels of *FtPAK* at different time points after UV-B treatment.

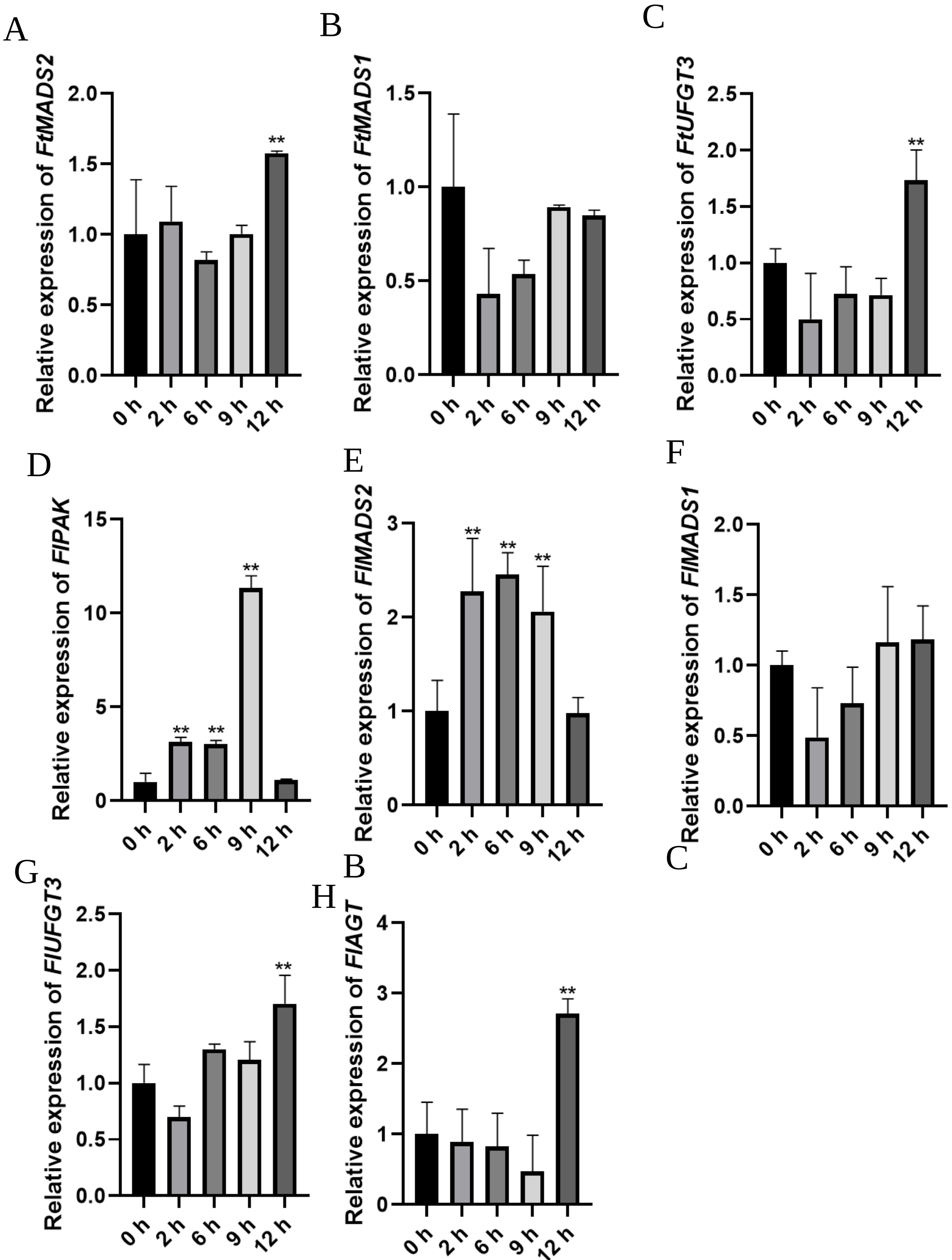

Supplemental Figure 56. Under UV-B stress, changes in the expression levels of each member of the *UFGT3* gene cluster. A) Relative expression level of *FtMADS2*. B) Relative expression level of *FtMADS1*. C) Relative expression level of *FtUFGT3*. D) Relative expression level of *FlPAK*. E) Relative expression level of *FlMADS2*. F) Relative expression level of *FlMADS1*. G) Relative expression level of *FlUFGT3*. H) Relative expression level of *FlAGT*.

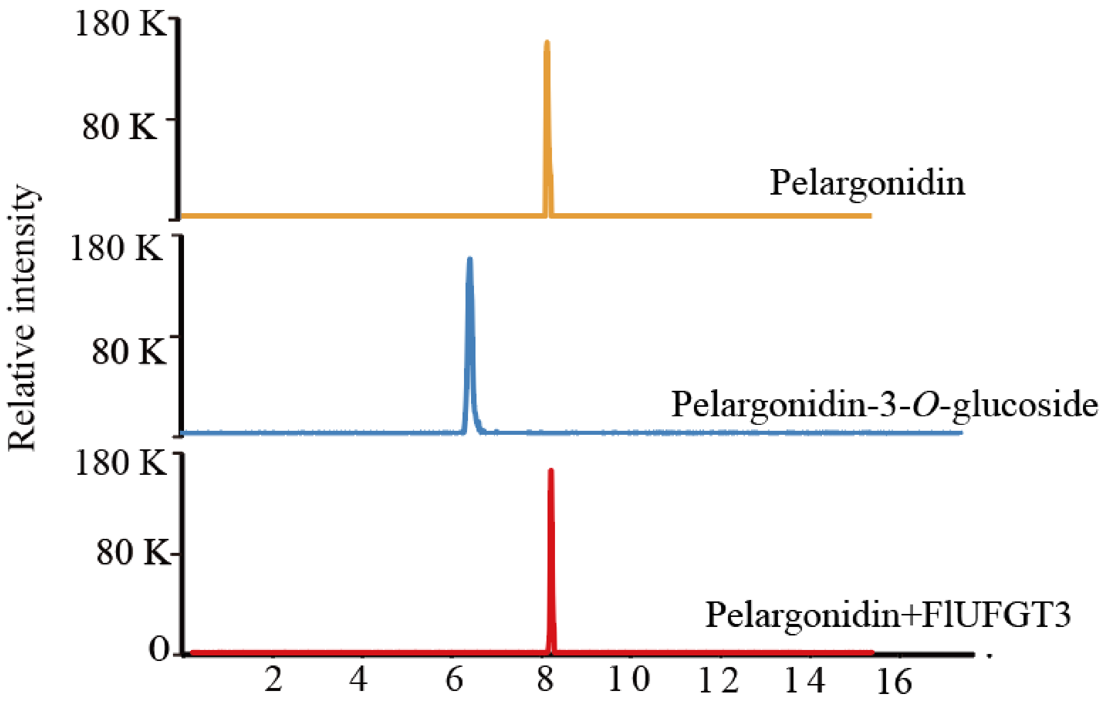

Supplemental Figure 57. Enzymatic assay for pelargonidin of FtUFGT3 *in vitro*.
